# Supplementary figures and images for: Molecular insights into the effect of 1,6-hexanediol on FUS phase separation
Source: EMBO J. 2025 Apr 25;44(10):2725–40. doi: 10.1038/s44318-025-00431-2 (PMC12084347; doi:10.1038/s44318-025-00431-2)

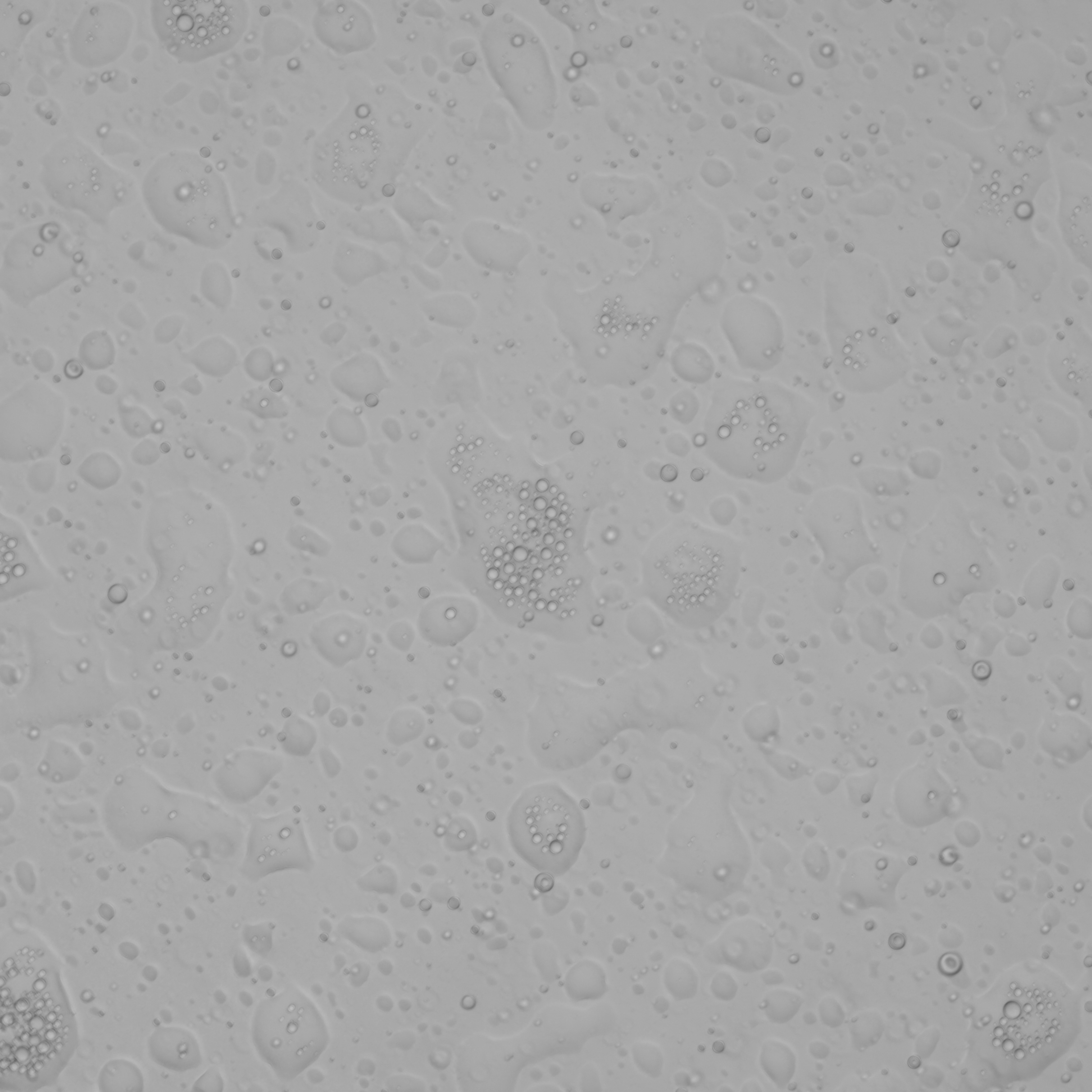

Supplement: Supplementary file 3 — Source data Fig. 1 [file 44318_2025_431_MOESM3_ESM.zip › Figure 1 copy/1A/Figure1A_No_additive.tif]

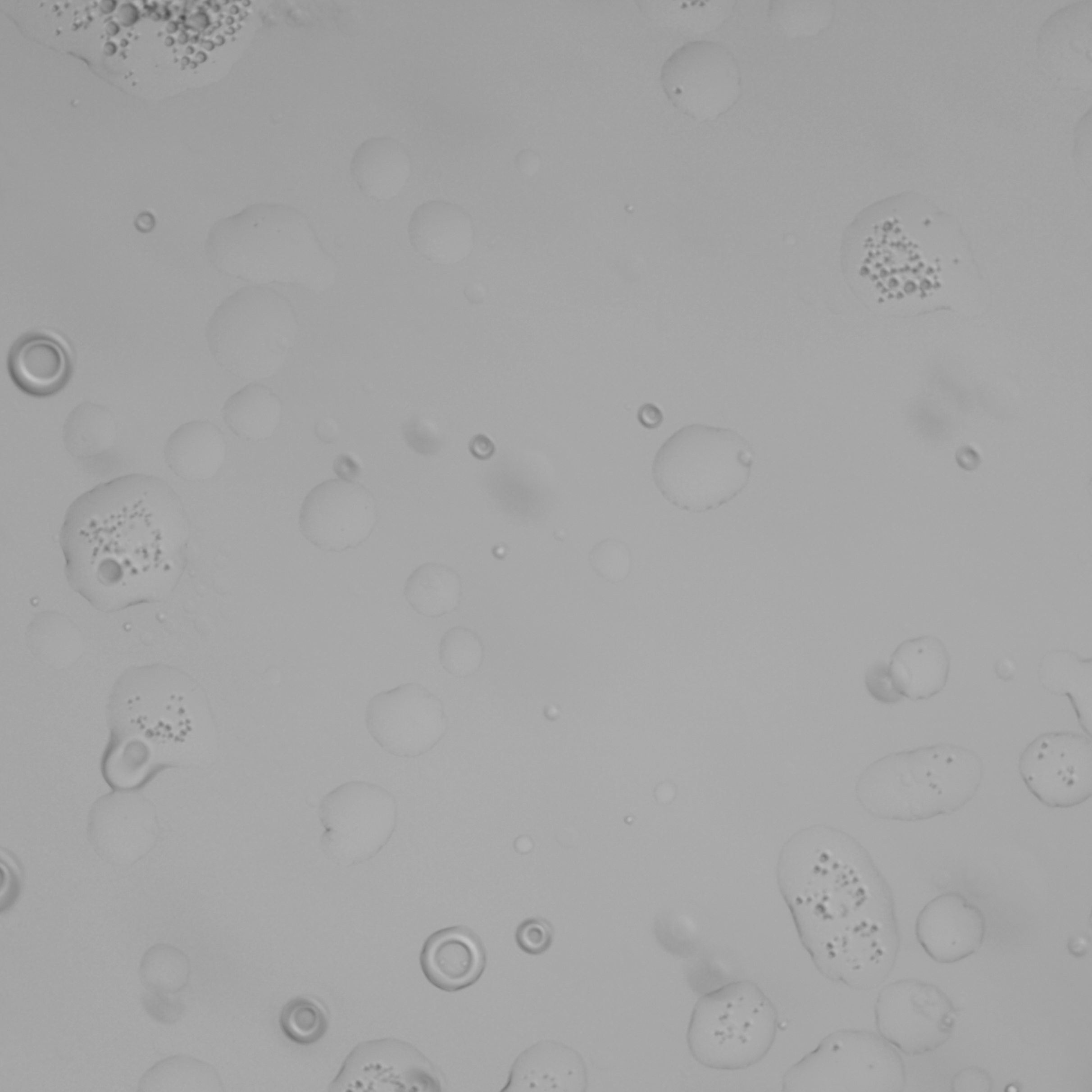

Supplement: Supplementary file 3 — Source data Fig. 1 [file 44318_2025_431_MOESM3_ESM.zip › Figure 1 copy/1A/Figure1A_2.0_percent.tif]

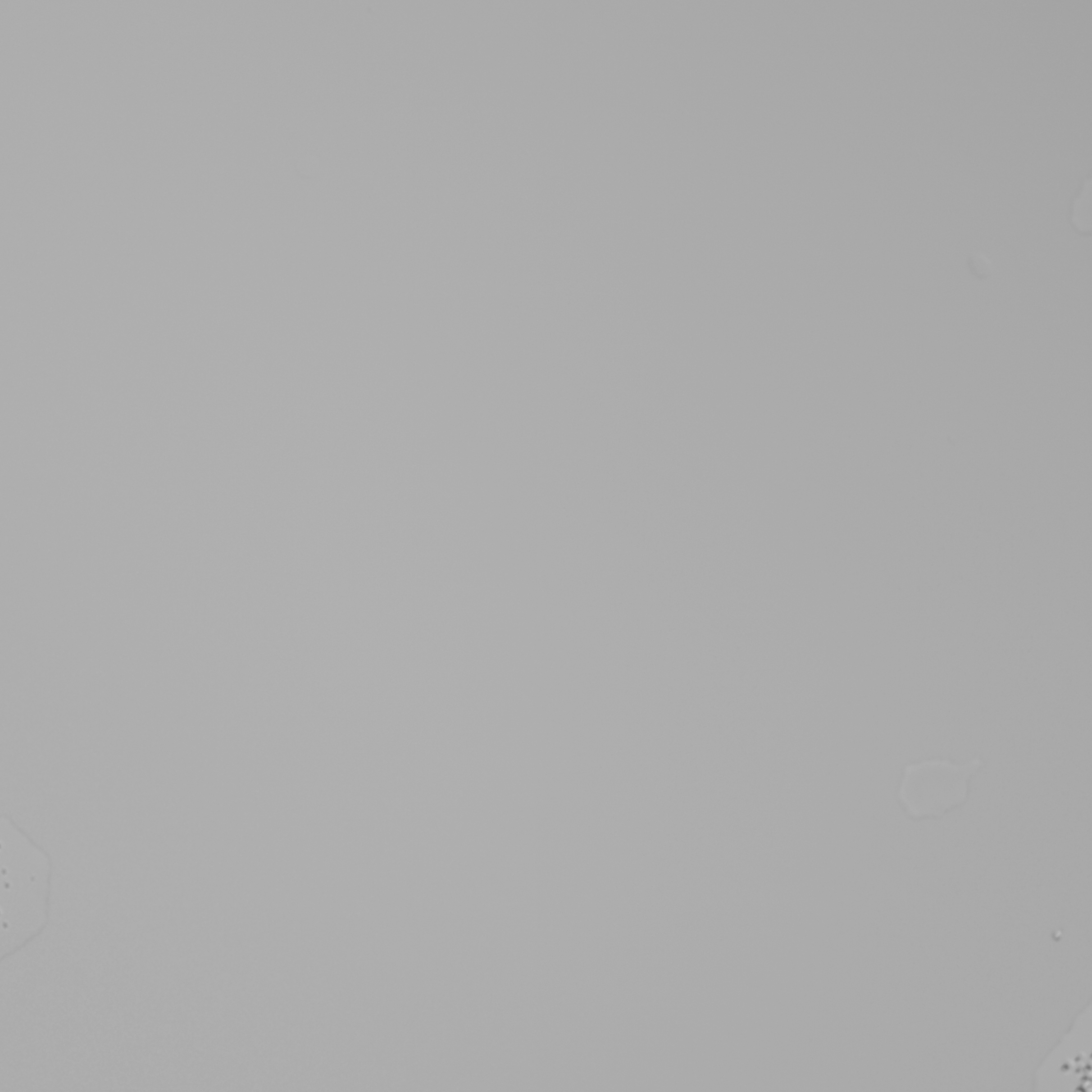

Supplement: Supplementary file 3 — Source data Fig. 1 [file 44318_2025_431_MOESM3_ESM.zip › Figure 1 copy/1A/Figure1A_5.0_percent.tif]

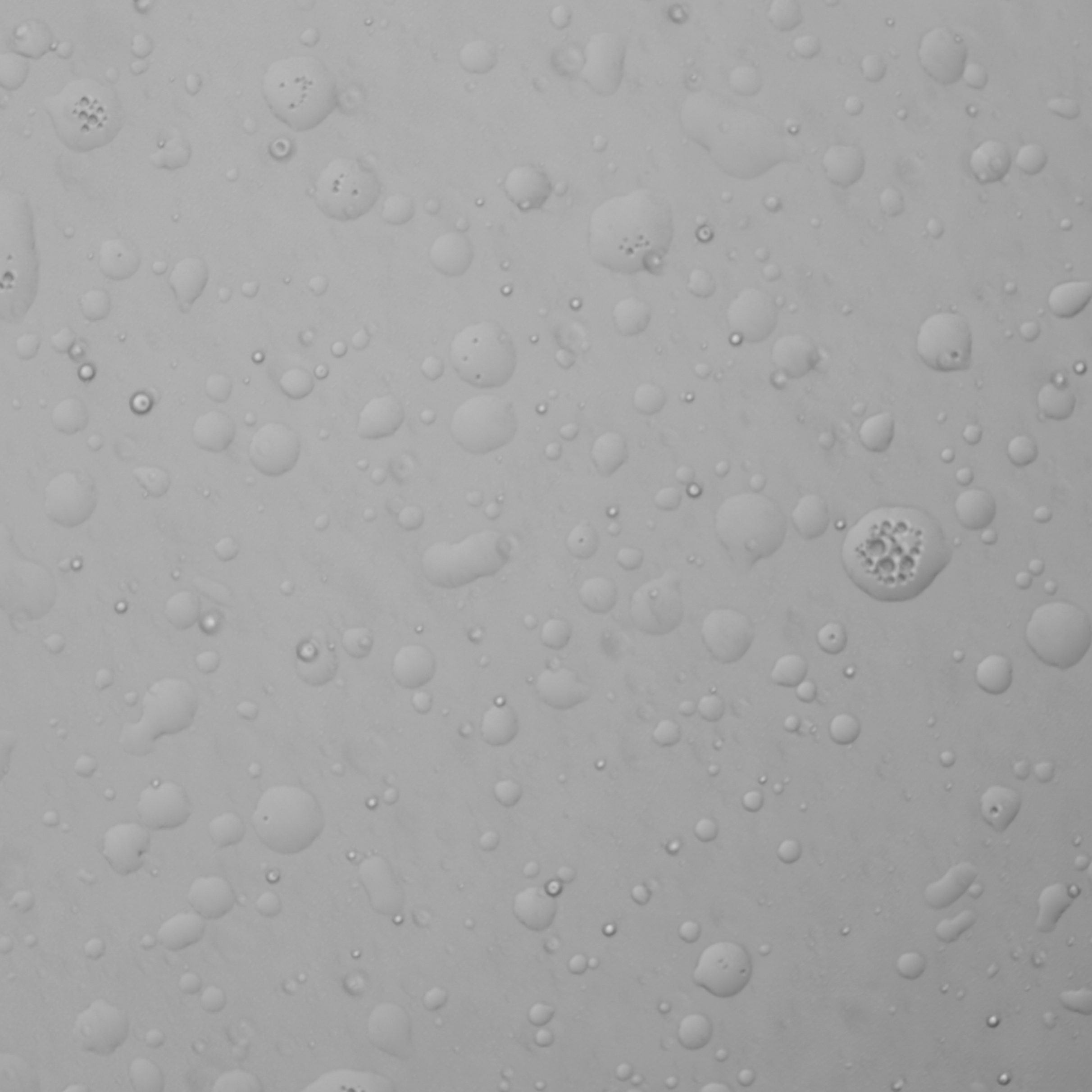

Supplement: Supplementary file 3 — Source data Fig. 1 [file 44318_2025_431_MOESM3_ESM.zip › Figure 1 copy/1A/Figure1A_1.0_percent.tif]

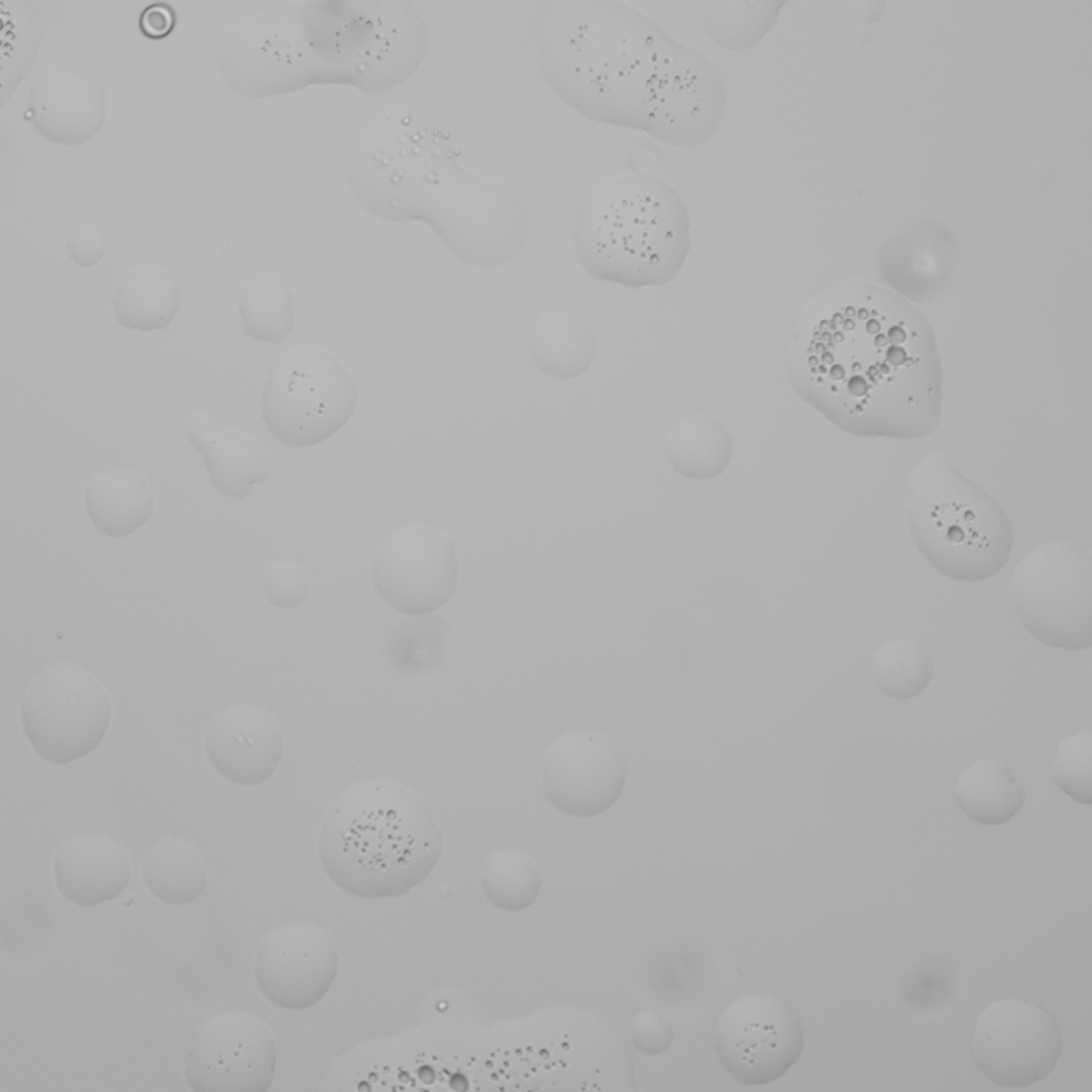

Supplement: Supplementary file 3 — Source data Fig. 1 [file 44318_2025_431_MOESM3_ESM.zip › Figure 1 copy/1A/Figure1A_2.5_percent.tif]

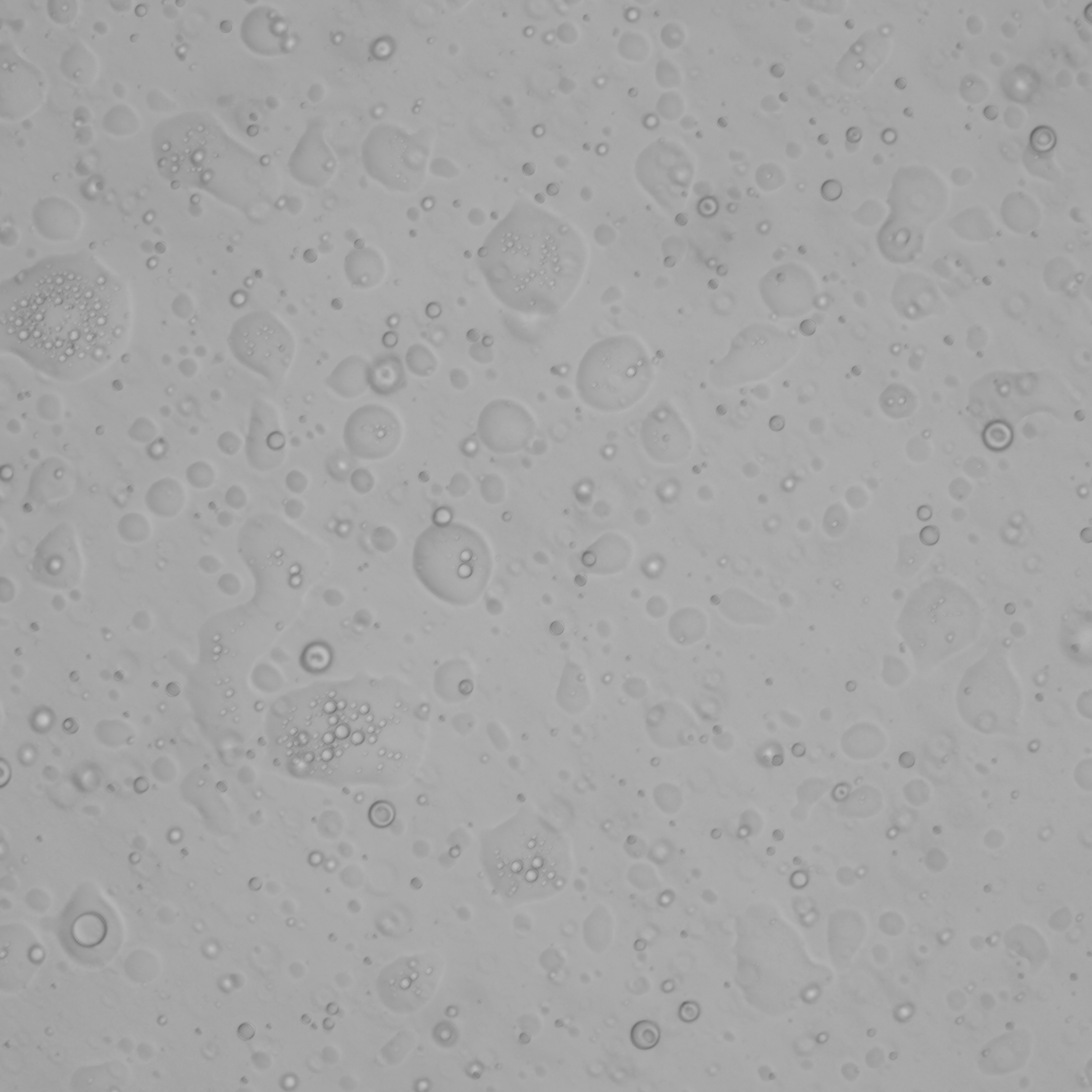

Supplement: Supplementary file 3 — Source data Fig. 1 [file 44318_2025_431_MOESM3_ESM.zip › Figure 1 copy/1A/Figure1A_0.5_percent.tif]

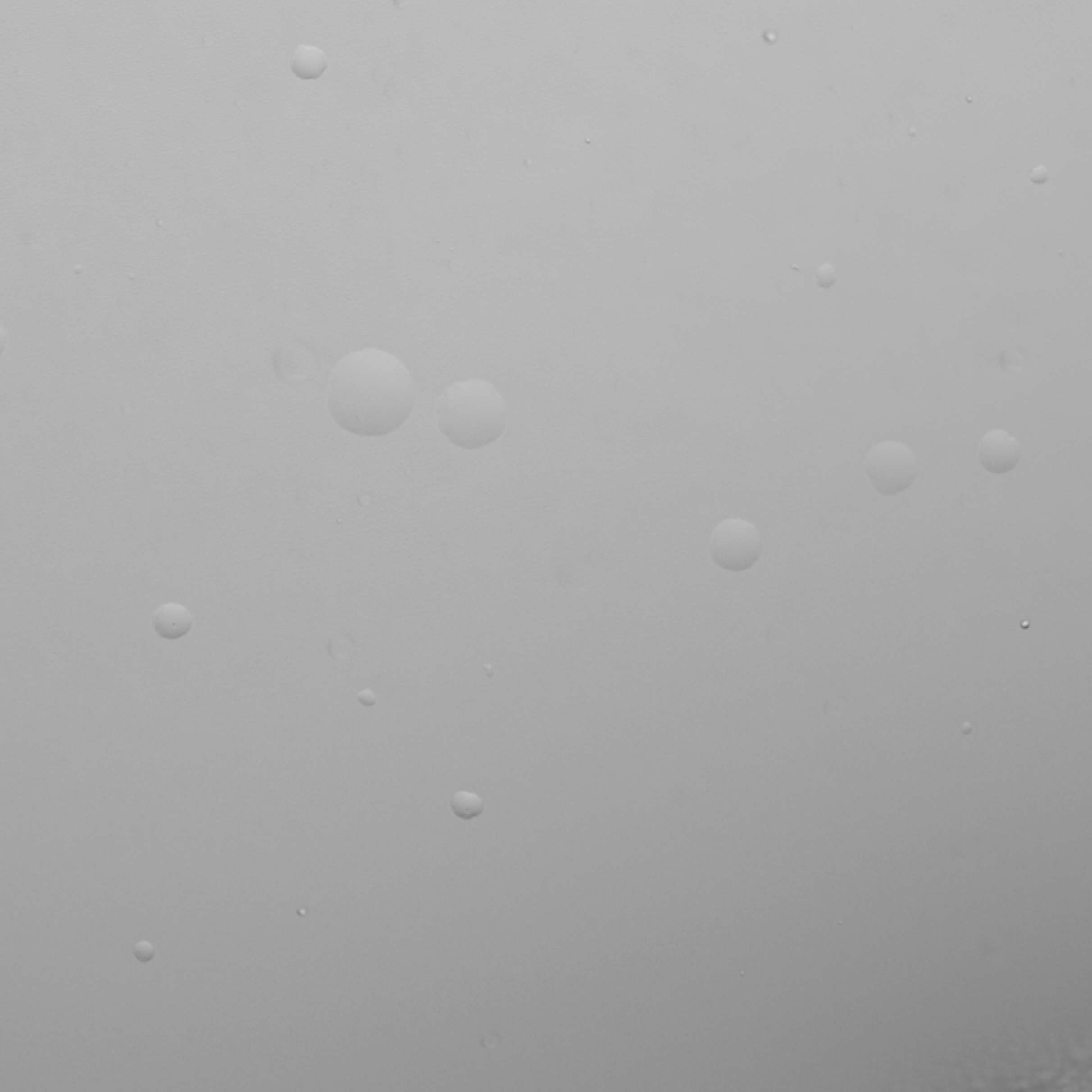

Supplement: Supplementary file 3 — Source data Fig. 1 [file 44318_2025_431_MOESM3_ESM.zip › Figure 1 copy/1D/Figure1D_16HD.tif]

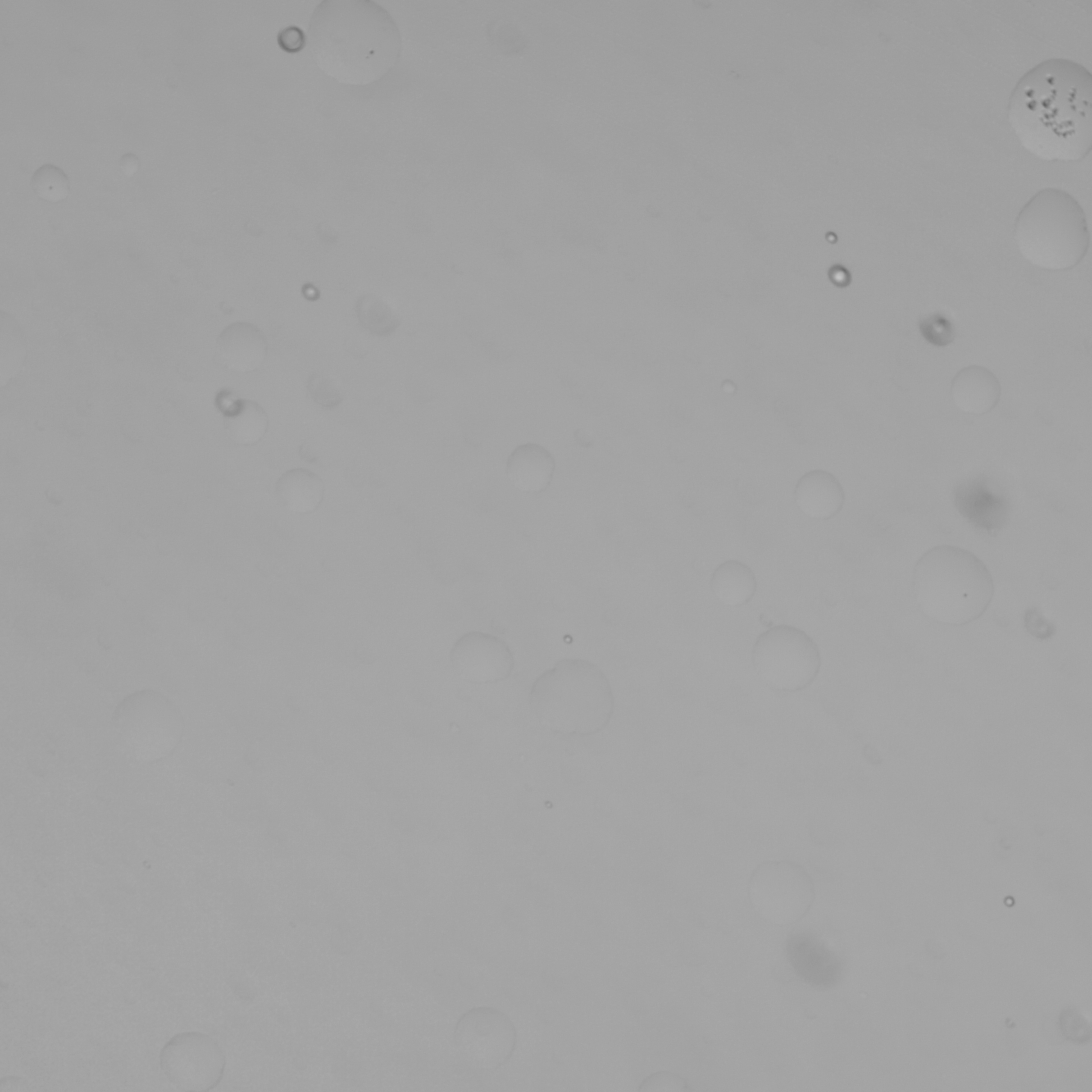

Supplement: Supplementary file 3 — Source data Fig. 1 [file 44318_2025_431_MOESM3_ESM.zip › Figure 1 copy/1D/Figure1D_12HD.tif]

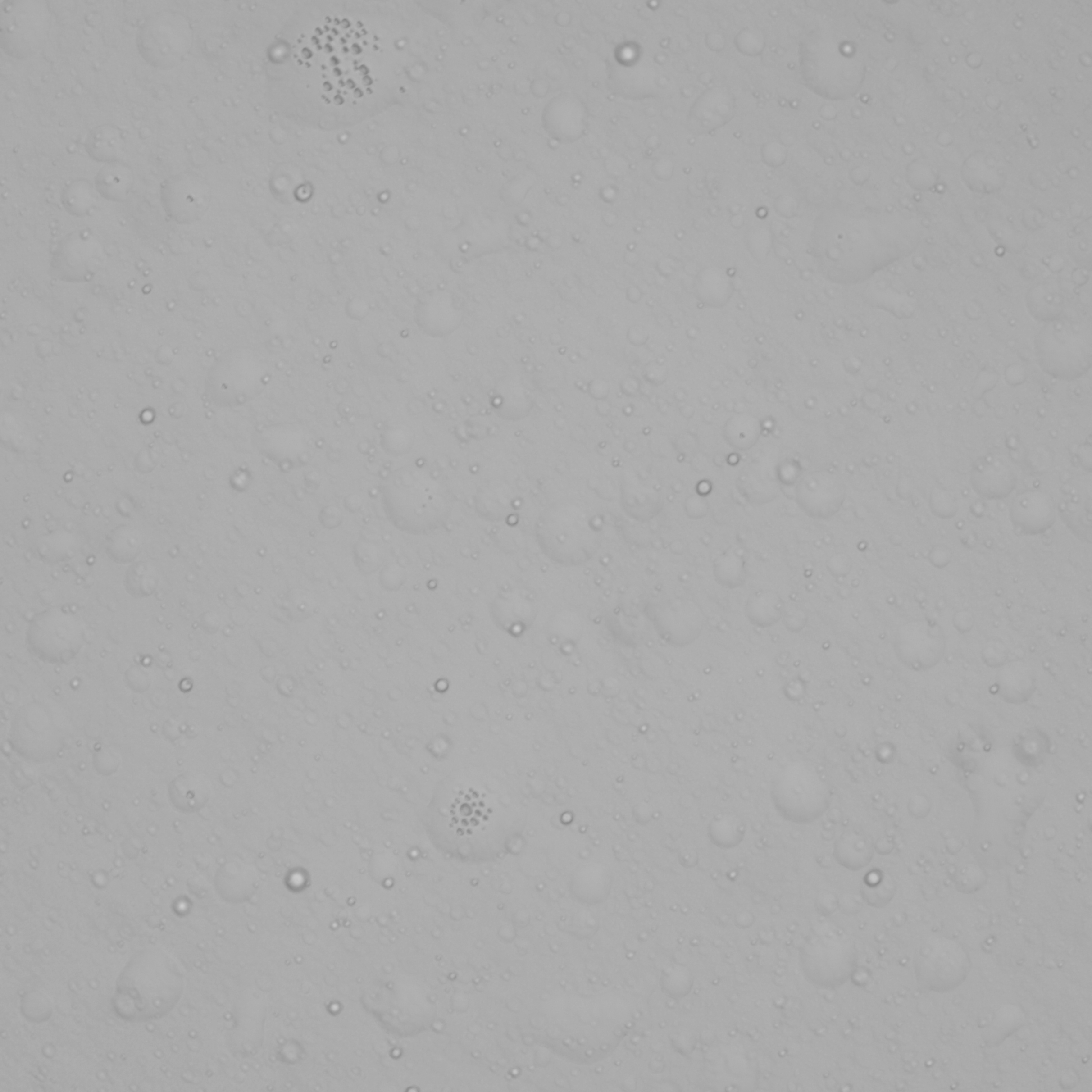

Supplement: Supplementary file 3 — Source data Fig. 1 [file 44318_2025_431_MOESM3_ESM.zip › Figure 1 copy/1D/Figure1D_14BD.tif]

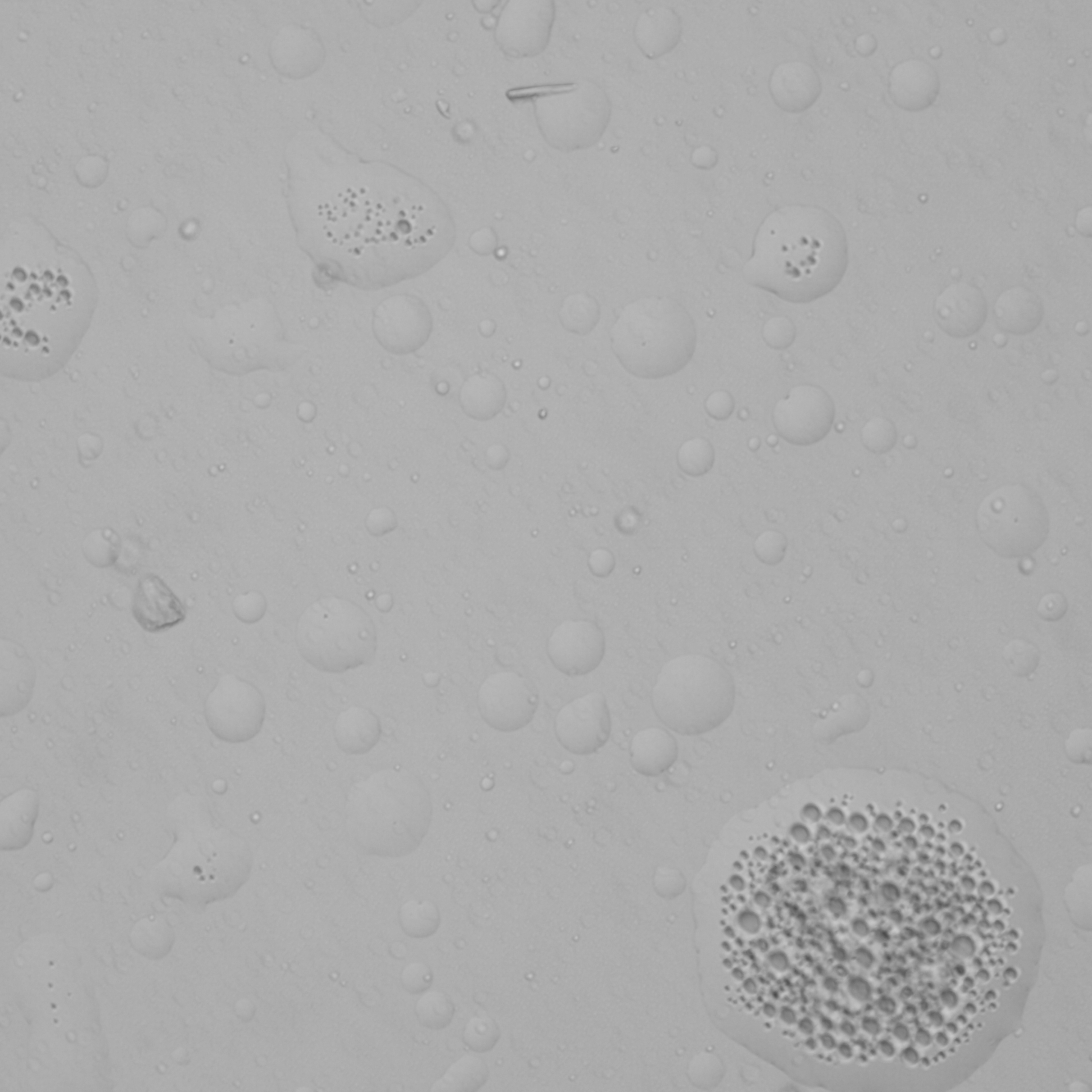

Supplement: Supplementary file 3 — Source data Fig. 1 [file 44318_2025_431_MOESM3_ESM.zip › Figure 1 copy/1D/Figure1D_15PD.tif]

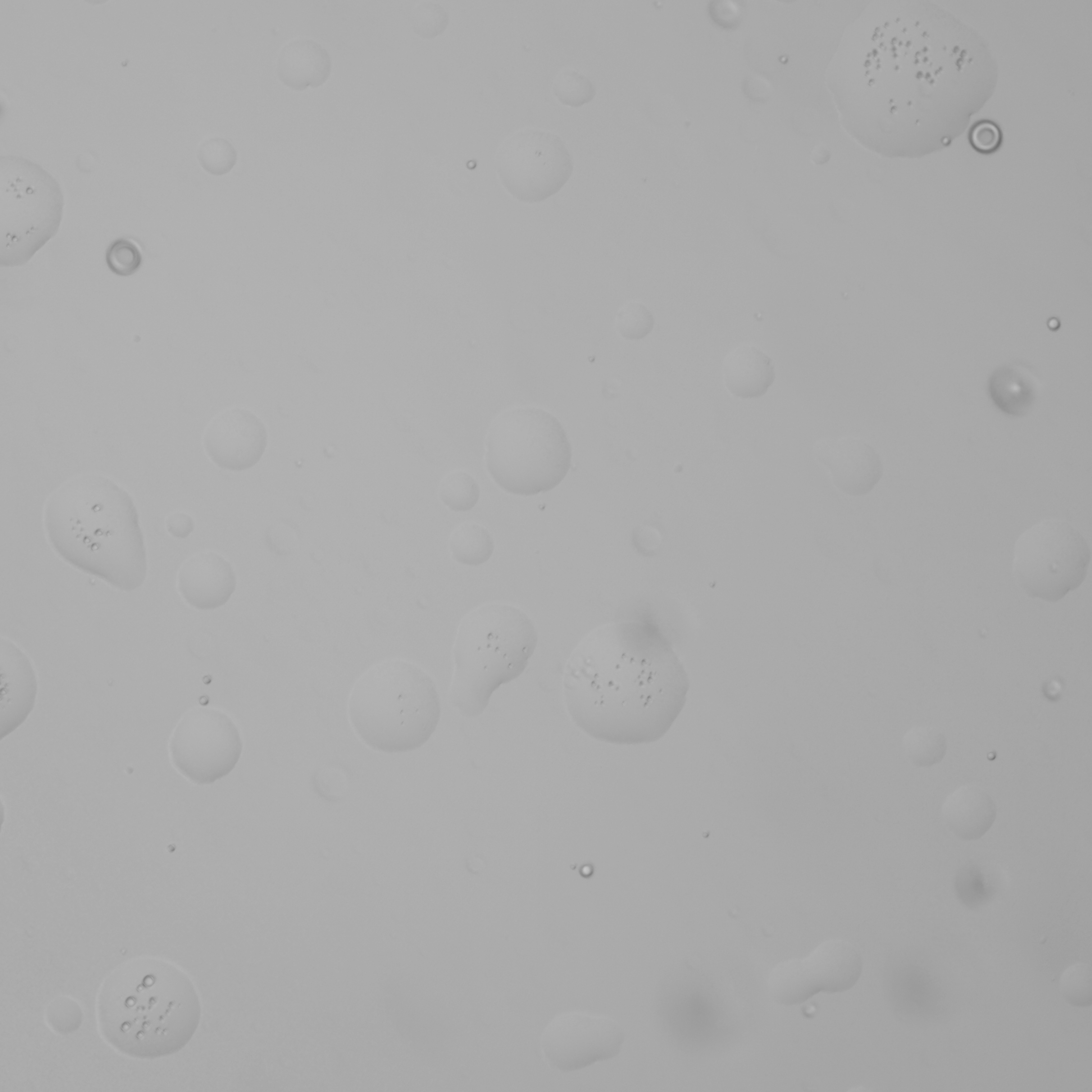

Supplement: Supplementary file 3 — Source data Fig. 1 [file 44318_2025_431_MOESM3_ESM.zip › Figure 1 copy/1D/Figure1D_12CHD.tif]

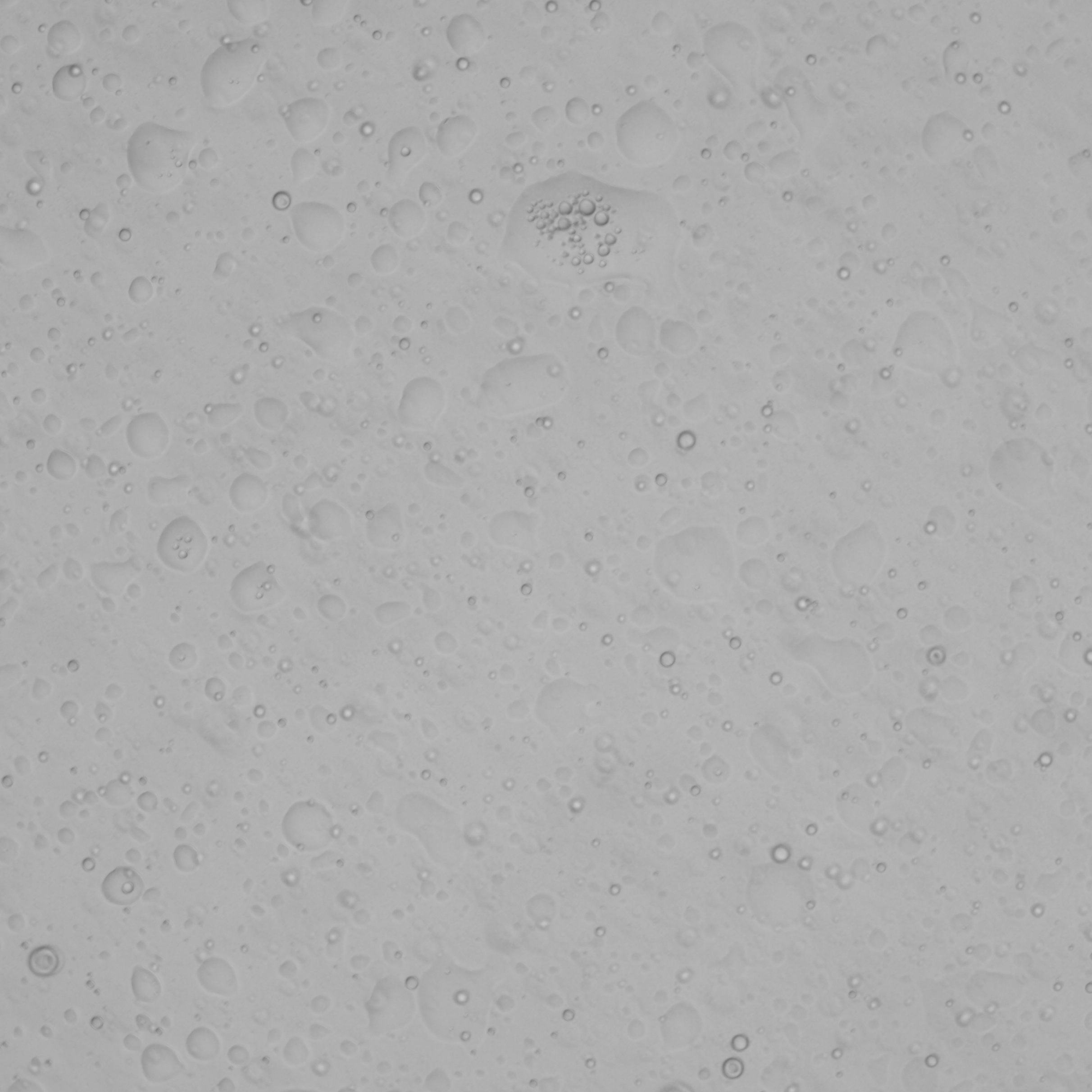

Supplement: Supplementary file 3 — Source data Fig. 1 [file 44318_2025_431_MOESM3_ESM.zip › Figure 1 copy/1D/Figure1D_No additive.tif]

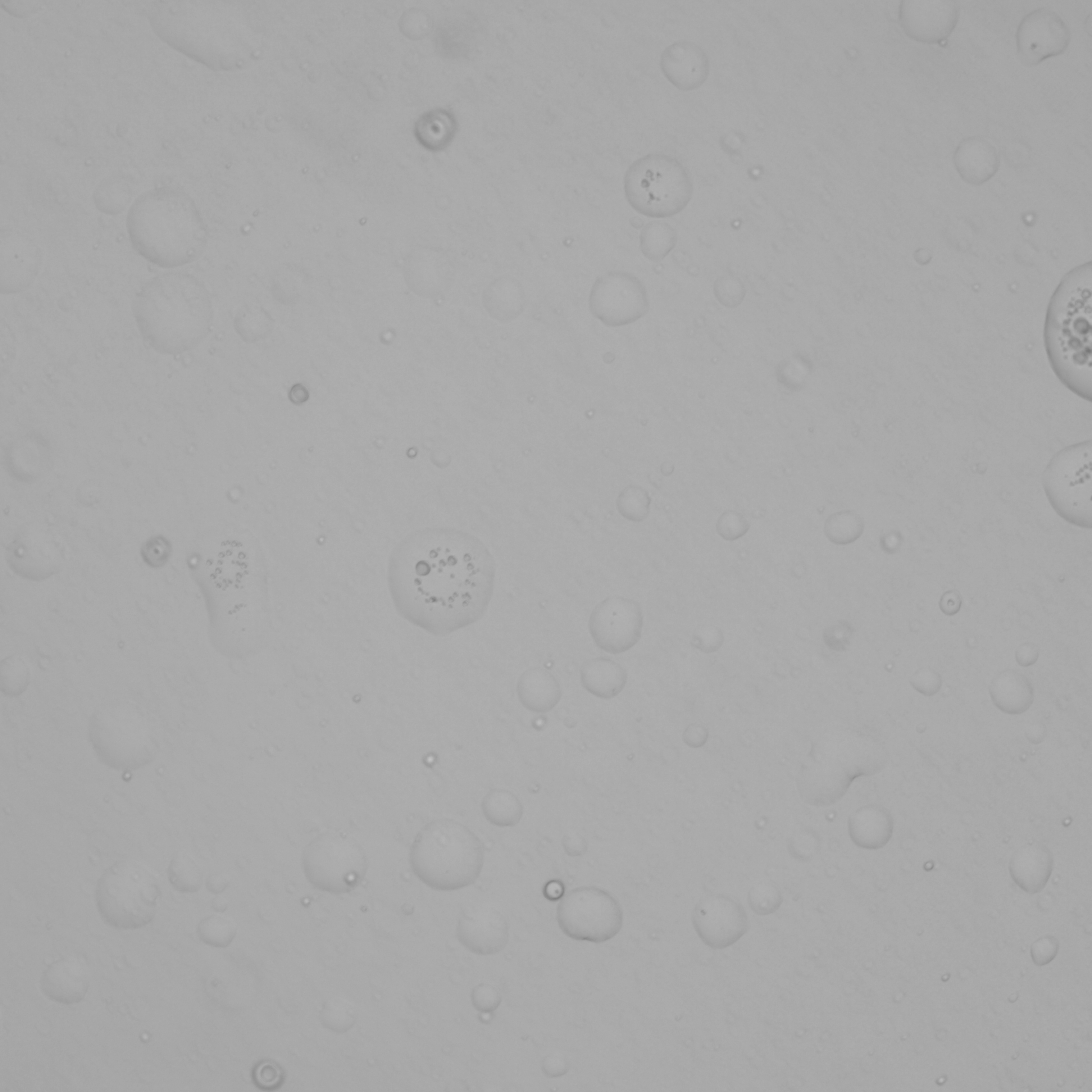

Supplement: Supplementary file 3 — Source data Fig. 1 [file 44318_2025_431_MOESM3_ESM.zip › Figure 1 copy/1D/Figure1D_25HD.tif]

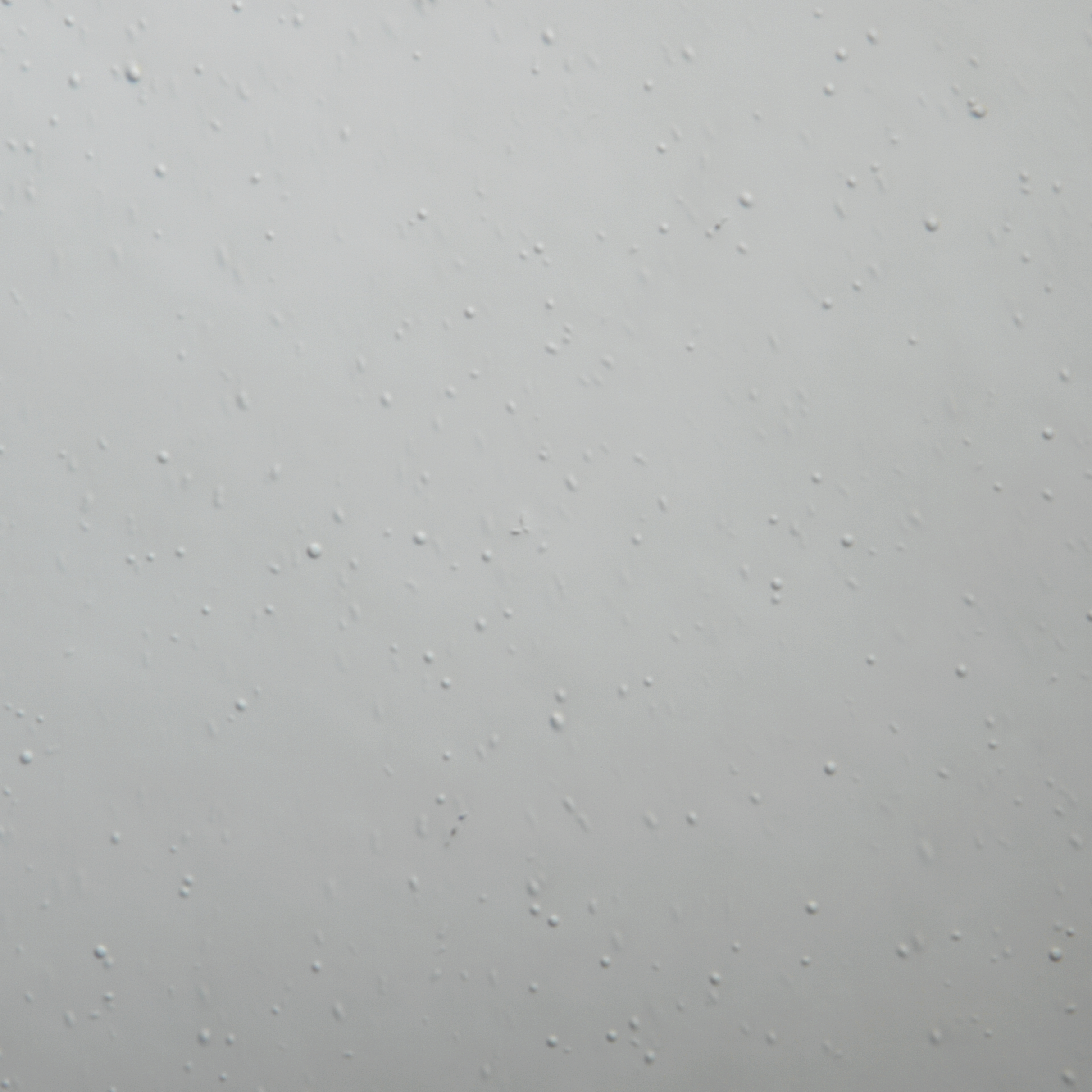

Supplement: Supplementary file 4 — Source data Fig. 2 [file 44318_2025_431_MOESM4_ESM.zip › Figure 2 copy/2E/2mM polyK+polyD 10mM NaCl no HD enhanced.tif]

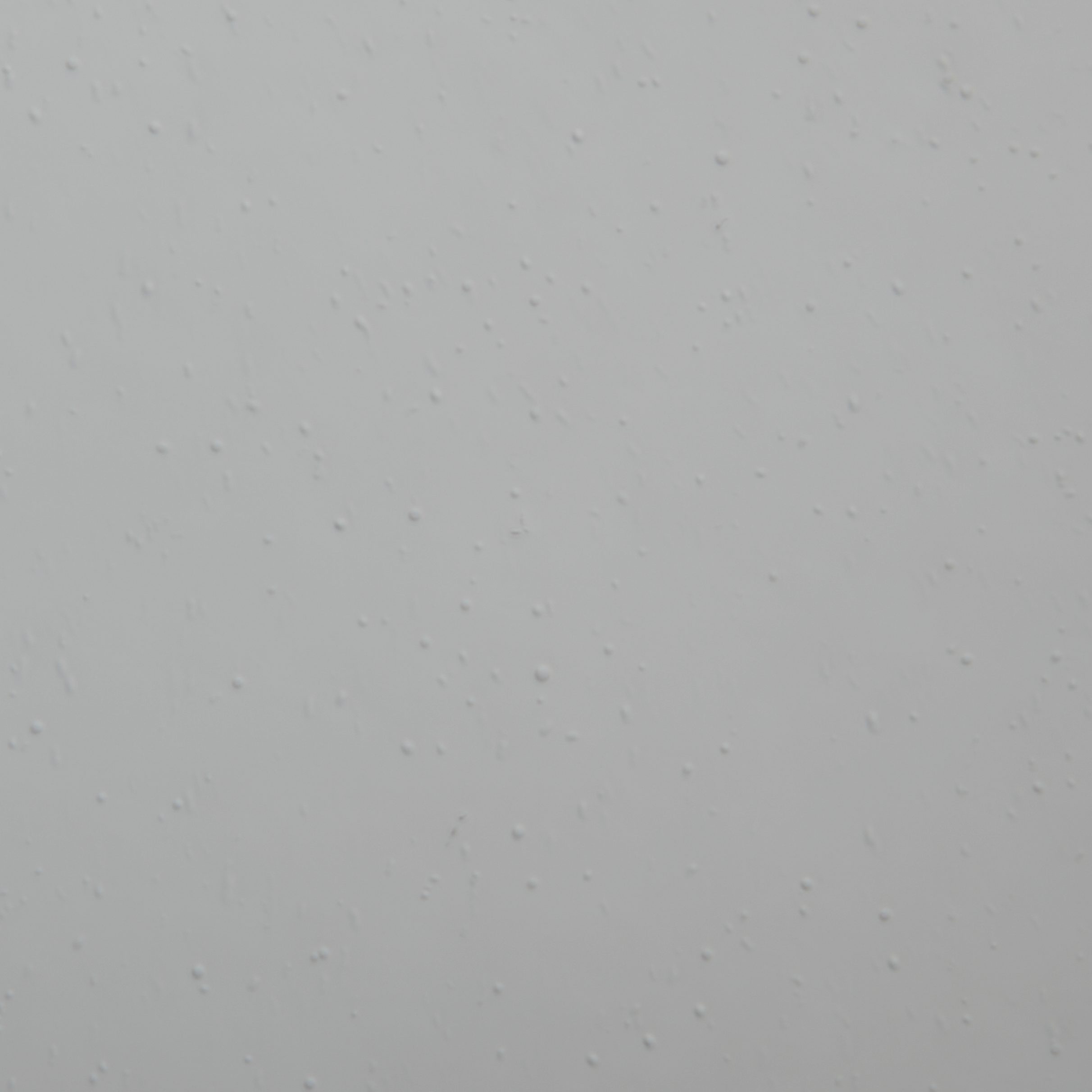

Supplement: Supplementary file 4 — Source data Fig. 2 [file 44318_2025_431_MOESM4_ESM.zip › Figure 2 copy/2E/2mM polyK+polyD 10mM NaCl no HD -1.tif]

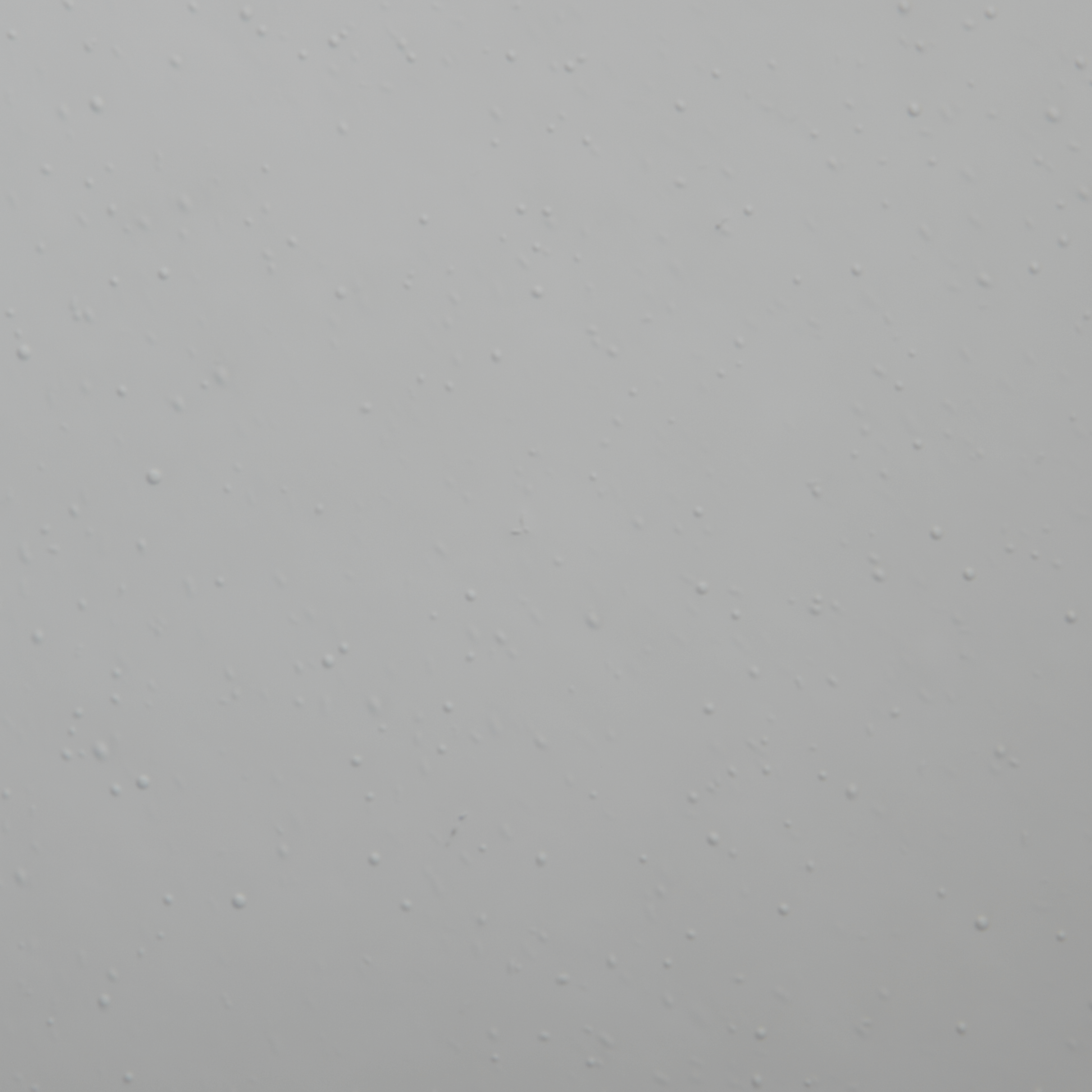

Supplement: Supplementary file 4 — Source data Fig. 2 [file 44318_2025_431_MOESM4_ESM.zip › Figure 2 copy/2E/2mM polyK+polyD 10mM NaCl no HD -3.tif]

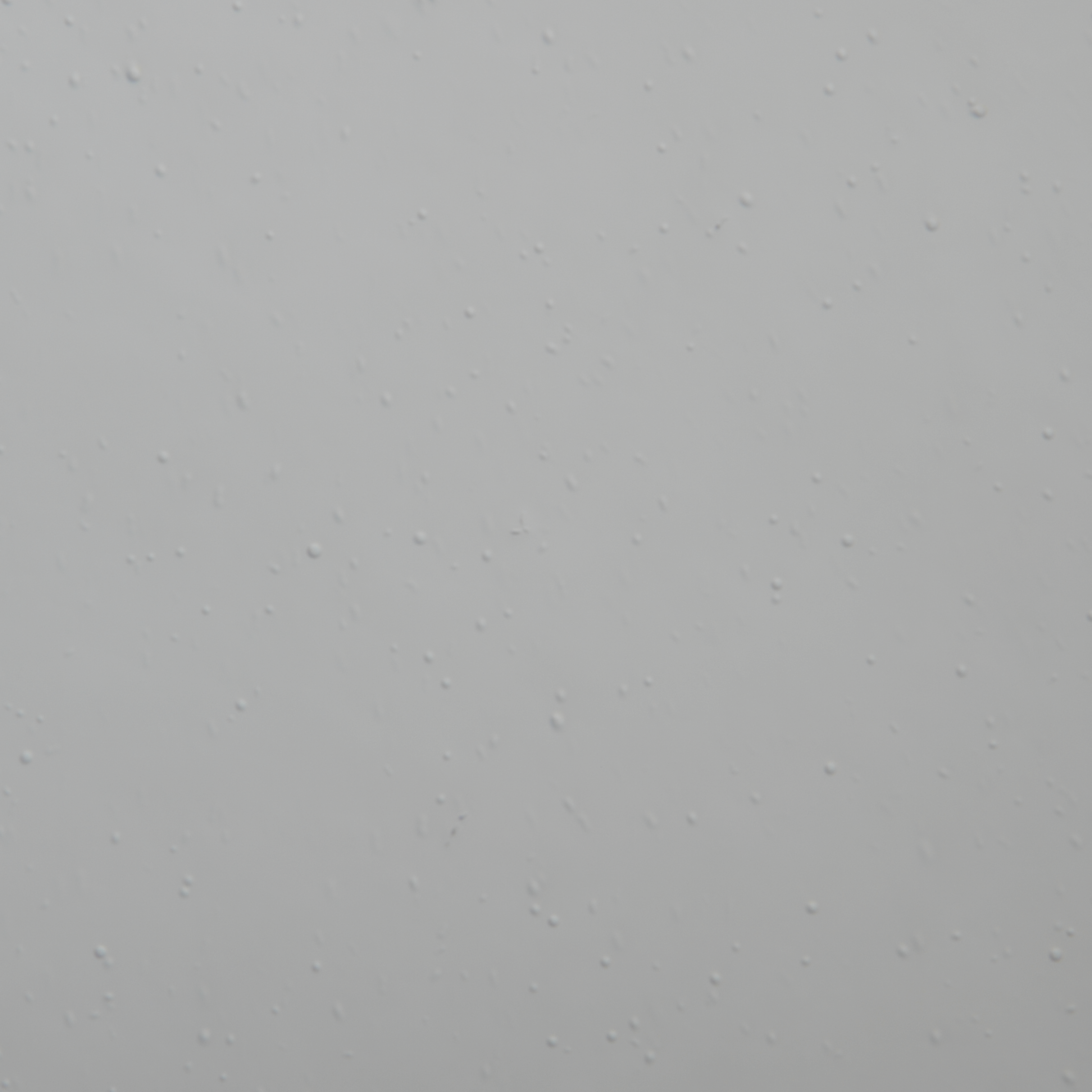

Supplement: Supplementary file 4 — Source data Fig. 2 [file 44318_2025_431_MOESM4_ESM.zip › Figure 2 copy/2E/2mM polyK+polyD 10mM NaCl no HD -2.tif]

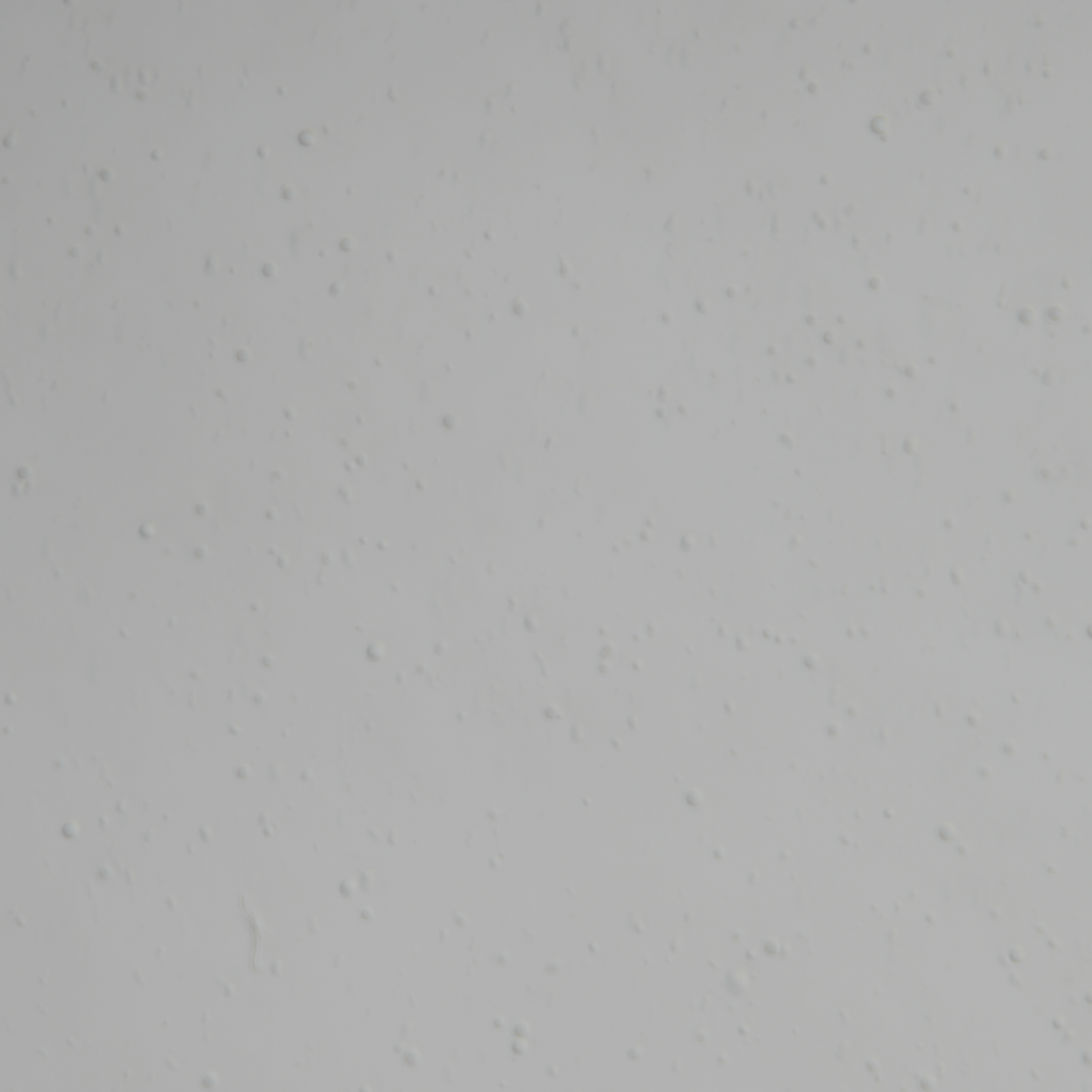

Supplement: Supplementary file 4 — Source data Fig. 2 [file 44318_2025_431_MOESM4_ESM.zip › Figure 2 copy/2E/2mM polyK+polyD 10mM NaCl 2% HD -1.tif]

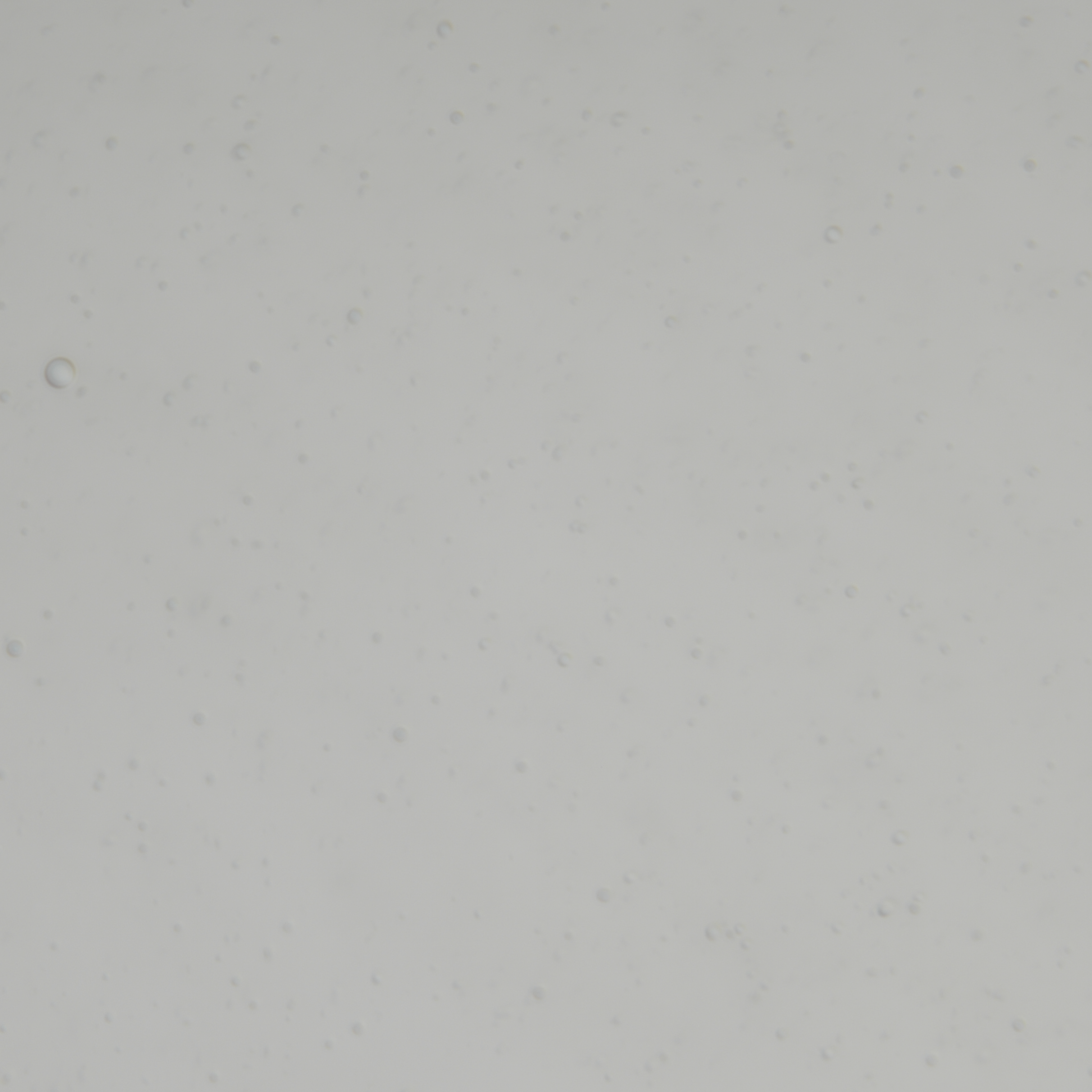

Supplement: Supplementary file 4 — Source data Fig. 2 [file 44318_2025_431_MOESM4_ESM.zip › Figure 2 copy/2E/2mM polyK+polyD 10mM NaCl 2% HD -3.tif]

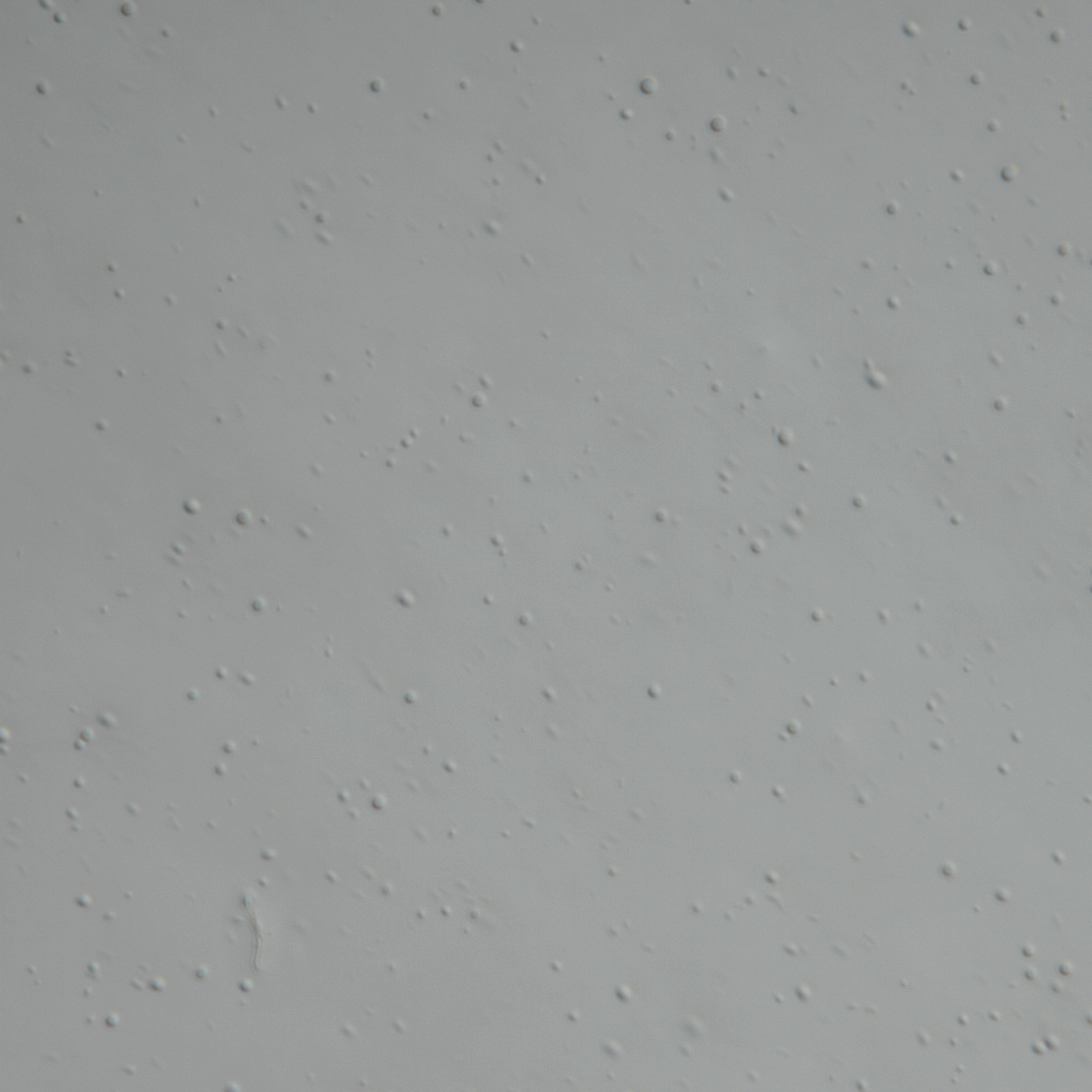

Supplement: Supplementary file 4 — Source data Fig. 2 [file 44318_2025_431_MOESM4_ESM.zip › Figure 2 copy/2E/2mM polyK+polyD 10mM NaCl 2% HD -4 enhanced.tif]

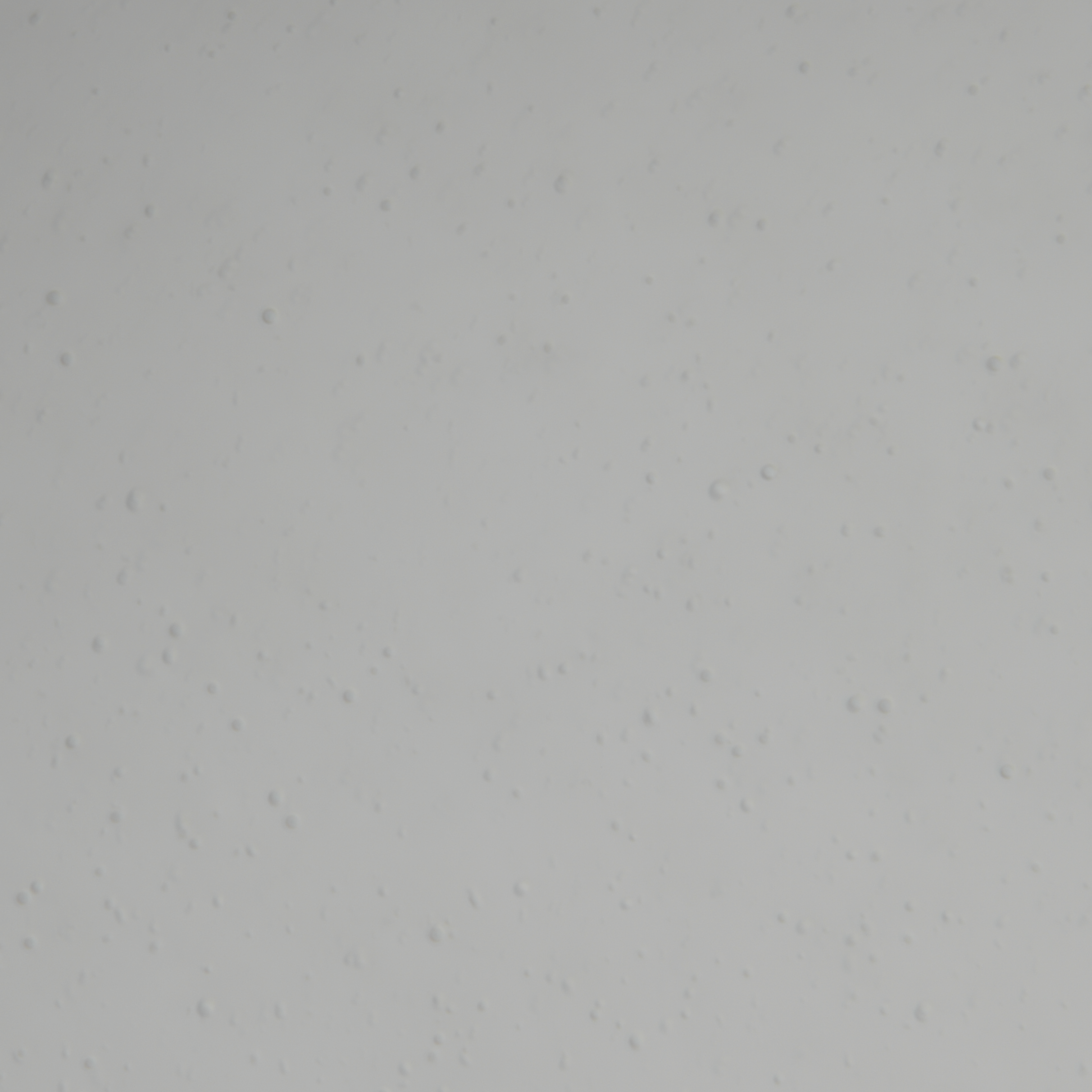

Supplement: Supplementary file 4 — Source data Fig. 2 [file 44318_2025_431_MOESM4_ESM.zip › Figure 2 copy/2E/2mM polyK+polyD 10mM NaCl 2% HD -2.tif]

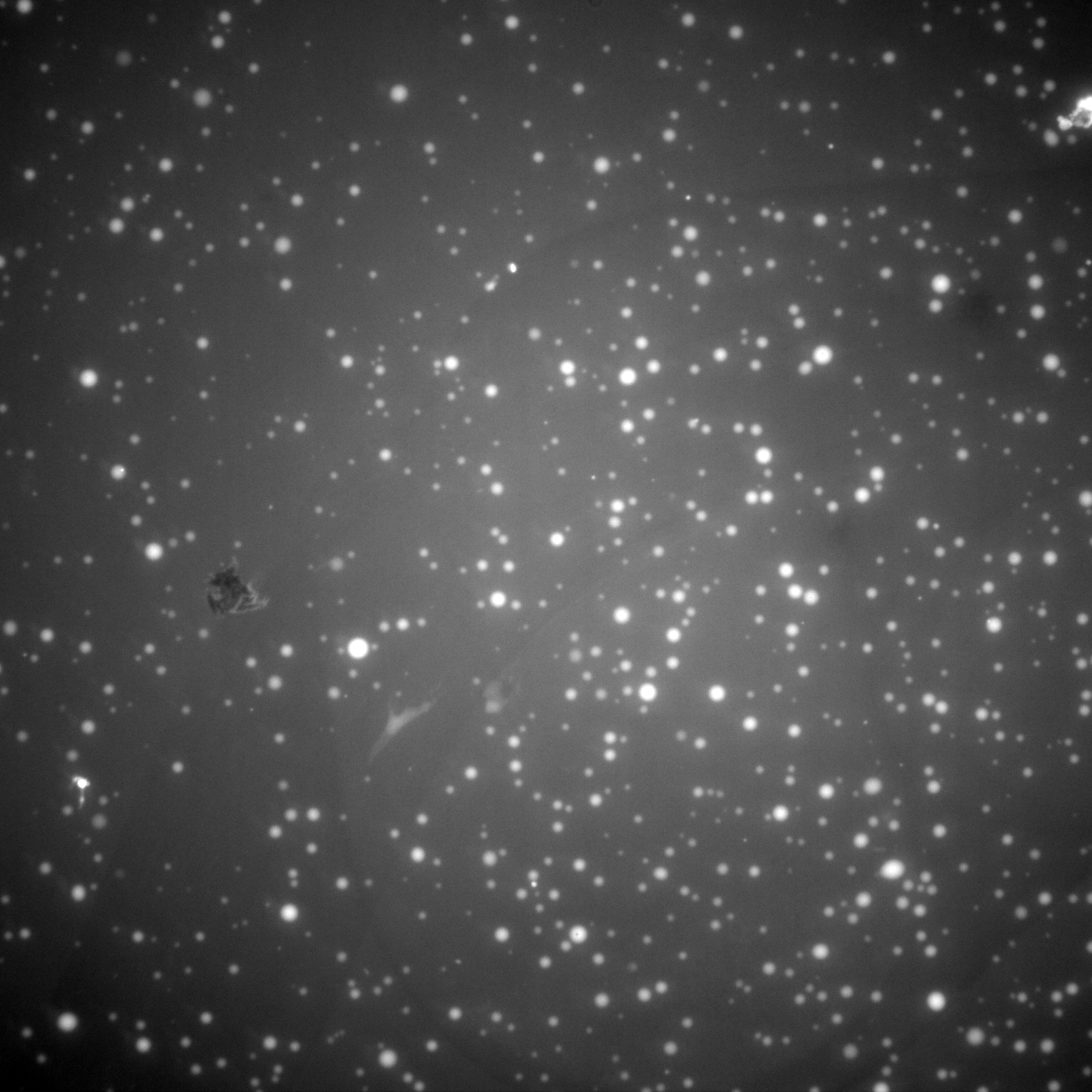

Supplement: Supplementary file 4 — Source data Fig. 2 [file 44318_2025_431_MOESM4_ESM.zip › Figure 2 copy/2A/NoRNA/INPUTDATA/2,5-HD 4.tif]

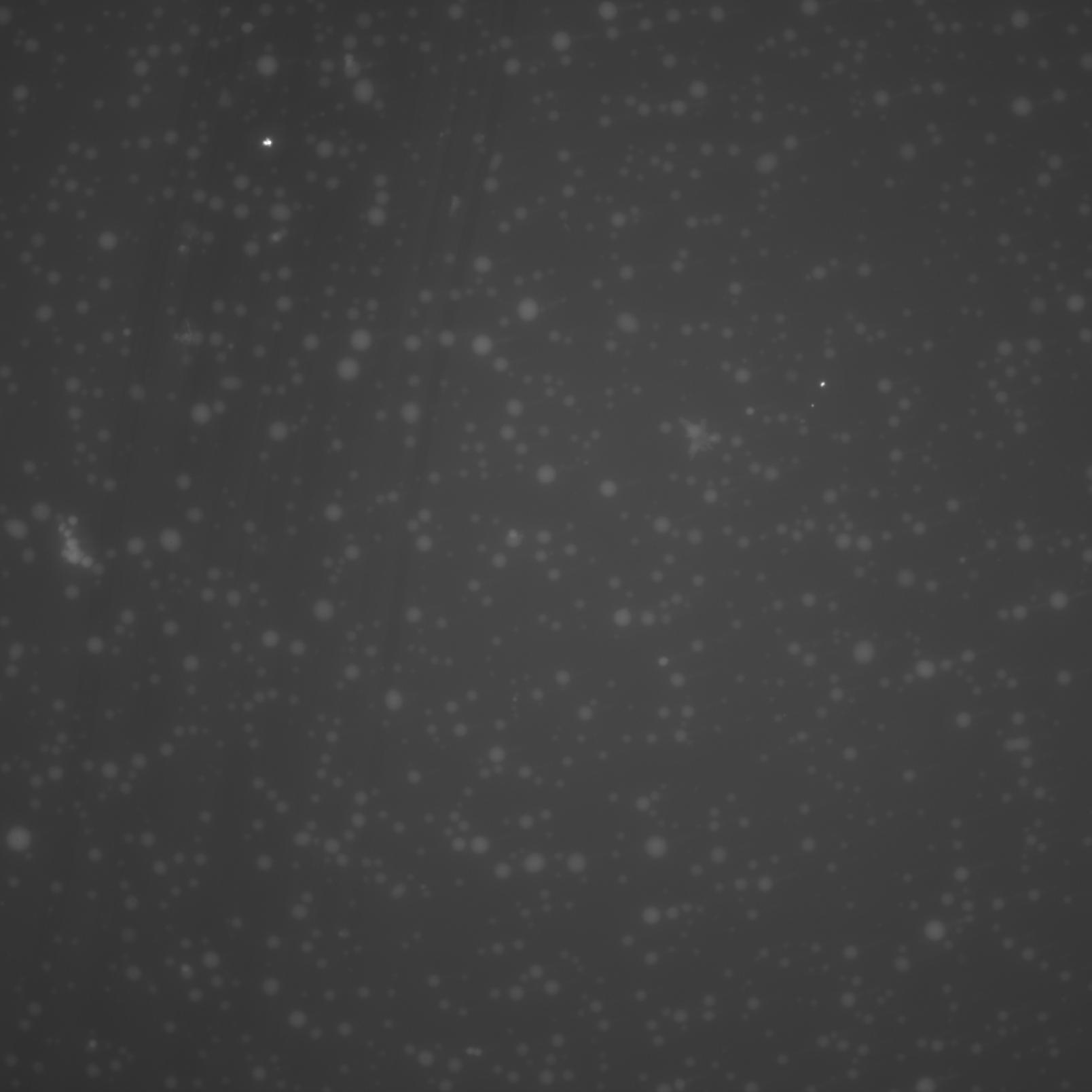

Supplement: Supplementary file 4 — Source data Fig. 2 [file 44318_2025_431_MOESM4_ESM.zip › Figure 2 copy/2A/NoRNA/INPUTDATA/2,5-HD 3.tif]

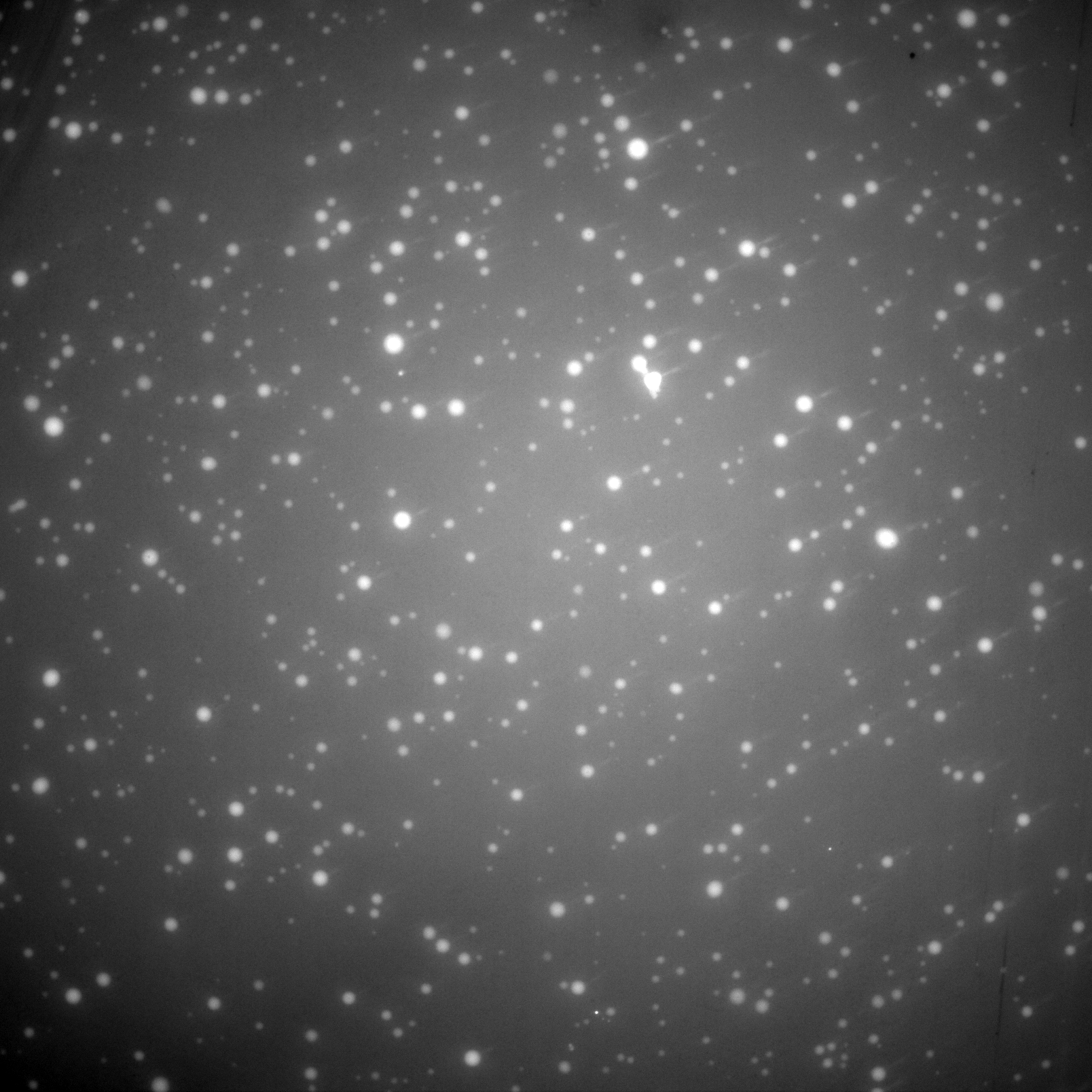

Supplement: Supplementary file 4 — Source data Fig. 2 [file 44318_2025_431_MOESM4_ESM.zip › Figure 2 copy/2A/NoRNA/INPUTDATA/2,5-HD 2.tif]

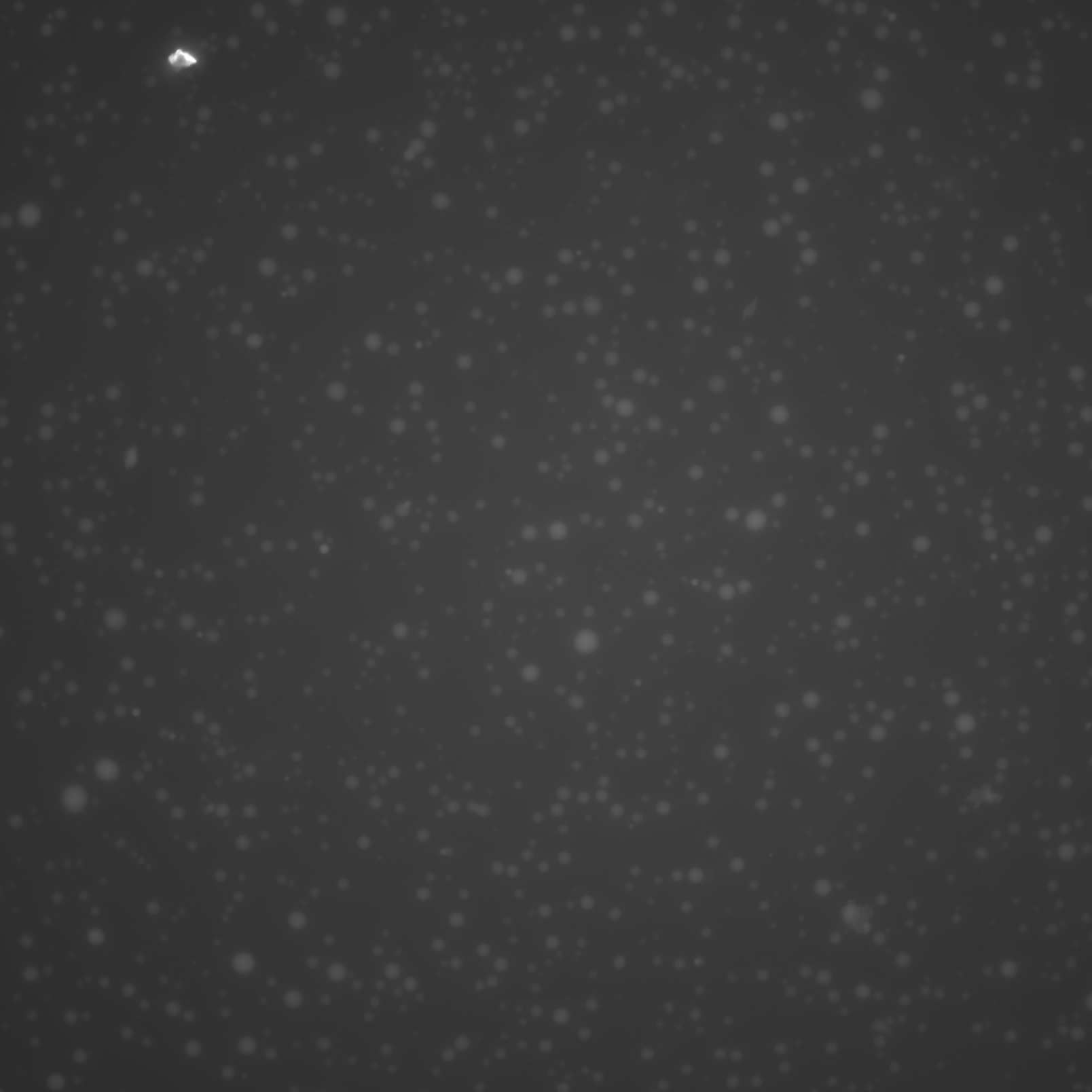

Supplement: Supplementary file 4 — Source data Fig. 2 [file 44318_2025_431_MOESM4_ESM.zip › Figure 2 copy/2A/NoRNA/INPUTDATA/2,5-HD 1.tif]

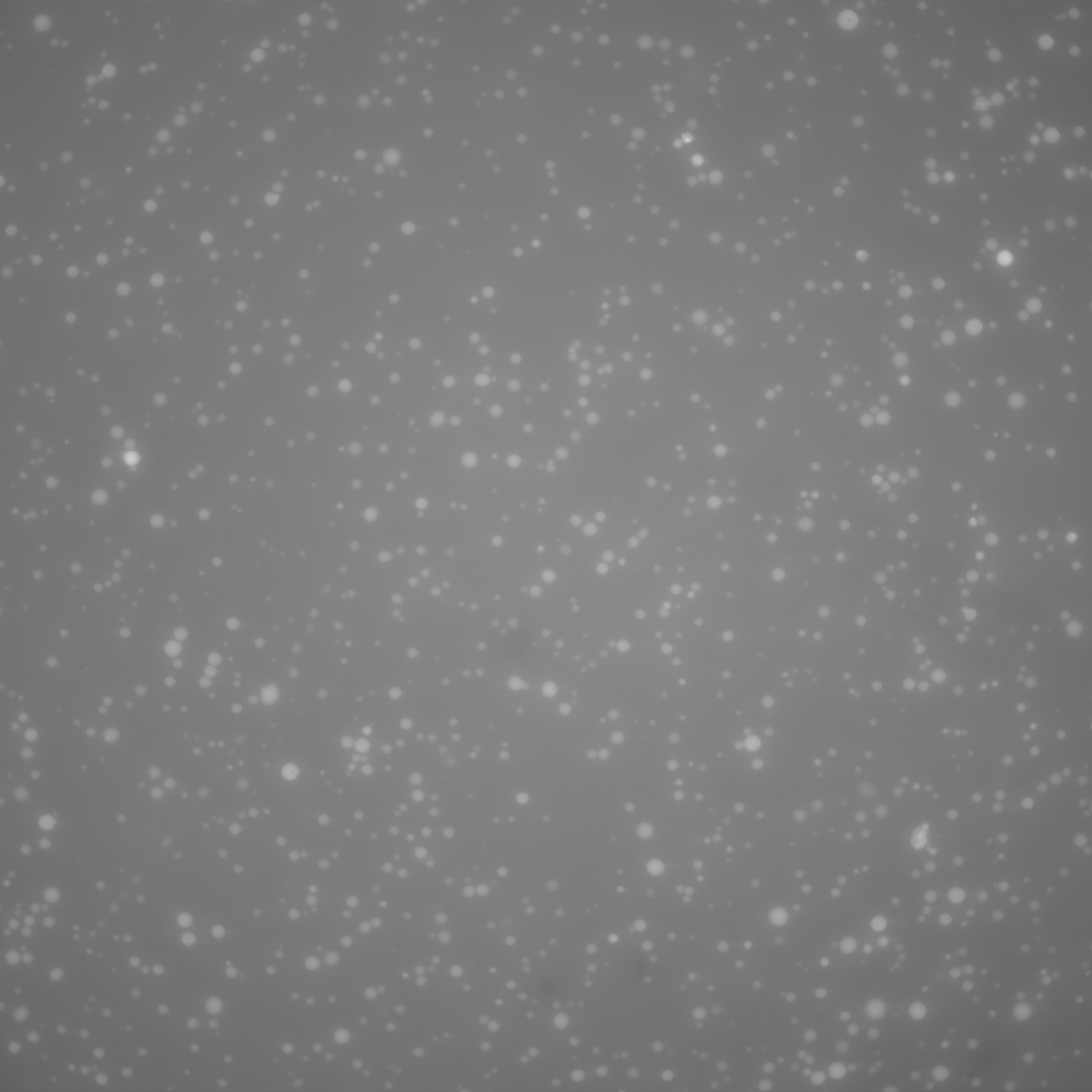

Supplement: Supplementary file 4 — Source data Fig. 2 [file 44318_2025_431_MOESM4_ESM.zip › Figure 2 copy/2A/NoRNA/INPUTDATA/no additive 1.tif]

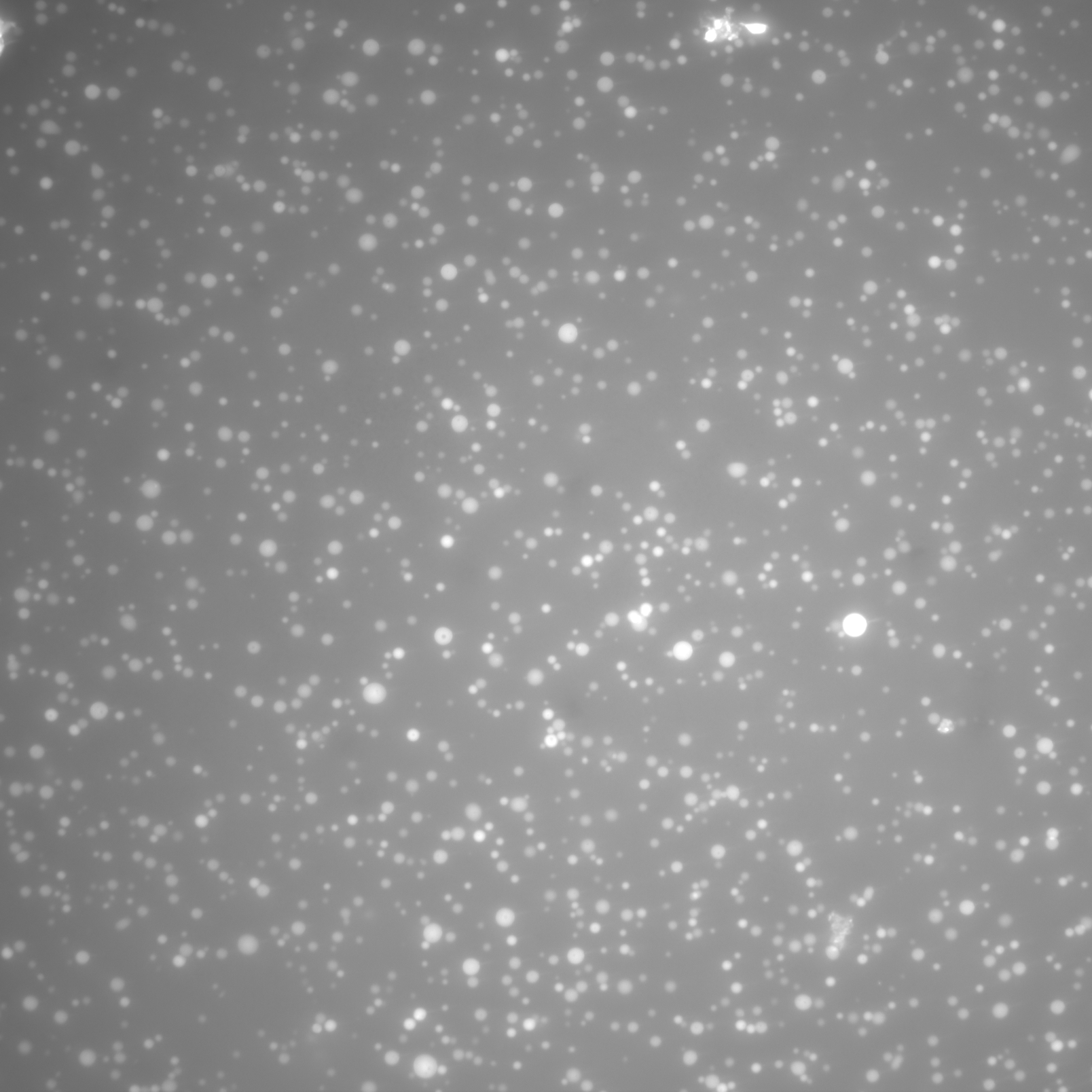

Supplement: Supplementary file 4 — Source data Fig. 2 [file 44318_2025_431_MOESM4_ESM.zip › Figure 2 copy/2A/NoRNA/INPUTDATA/no additive 3.tif]

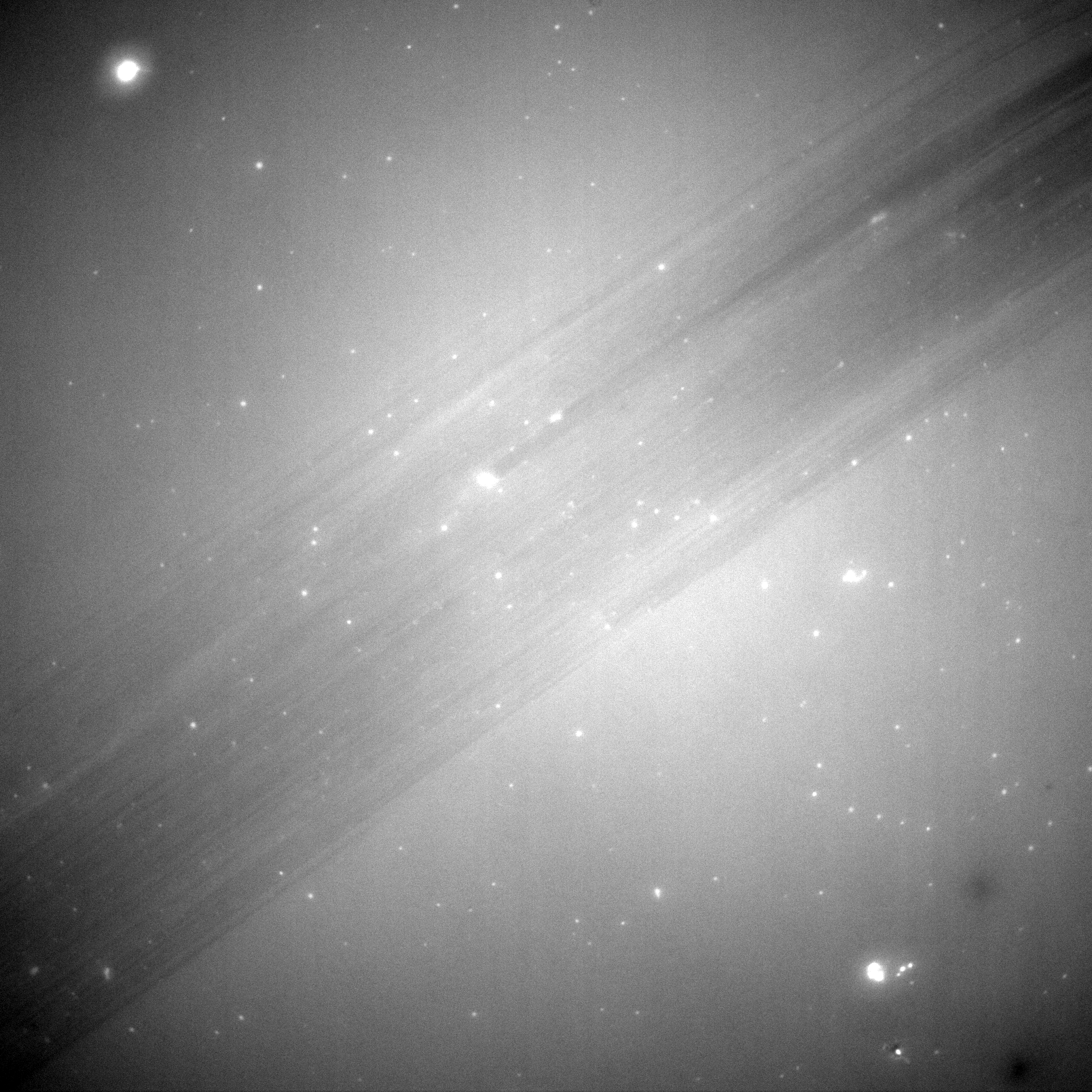

Supplement: Supplementary file 4 — Source data Fig. 2 [file 44318_2025_431_MOESM4_ESM.zip › Figure 2 copy/2A/NoRNA/INPUTDATA/1,6-HD 4.tif]

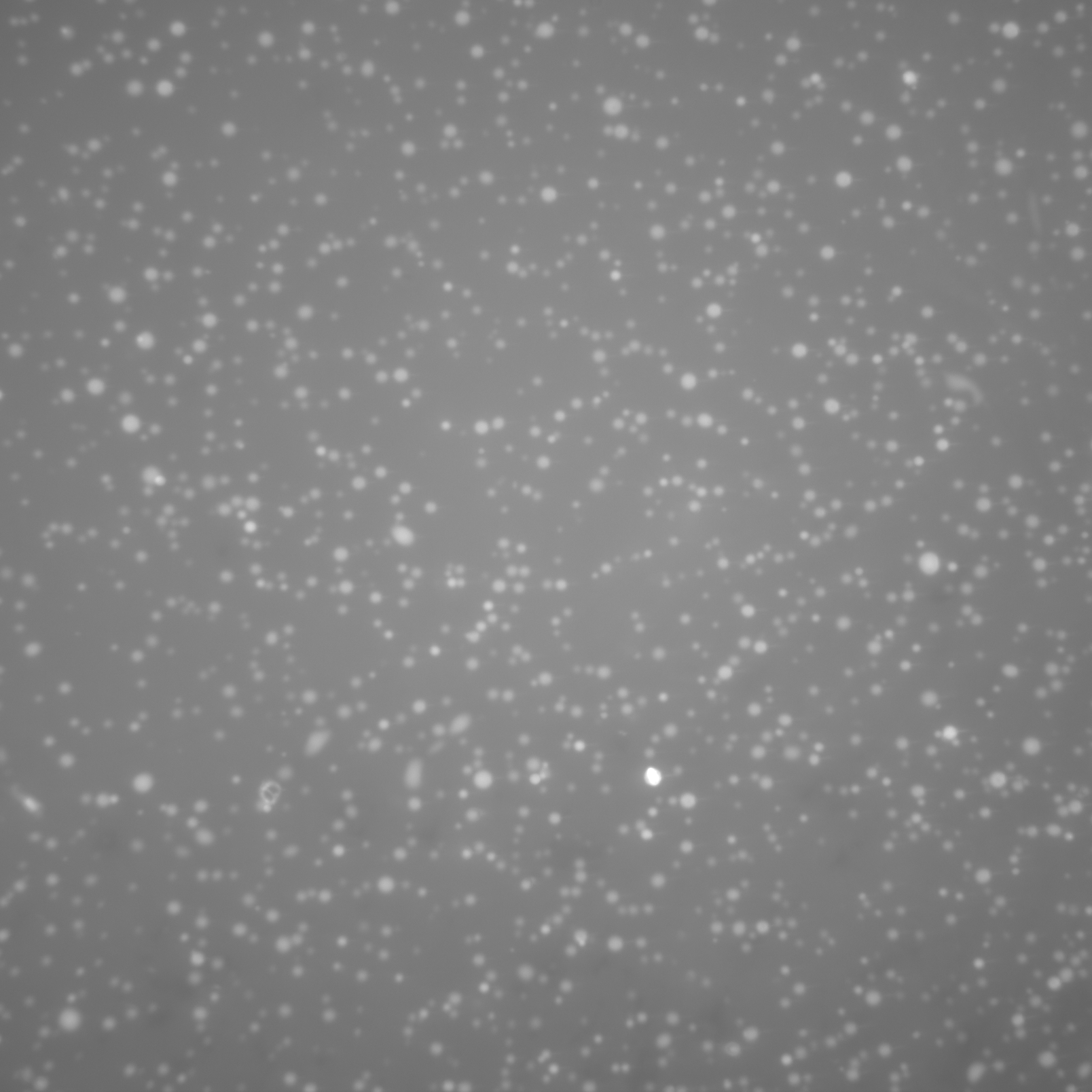

Supplement: Supplementary file 4 — Source data Fig. 2 [file 44318_2025_431_MOESM4_ESM.zip › Figure 2 copy/2A/NoRNA/INPUTDATA/no additive 2.tif]

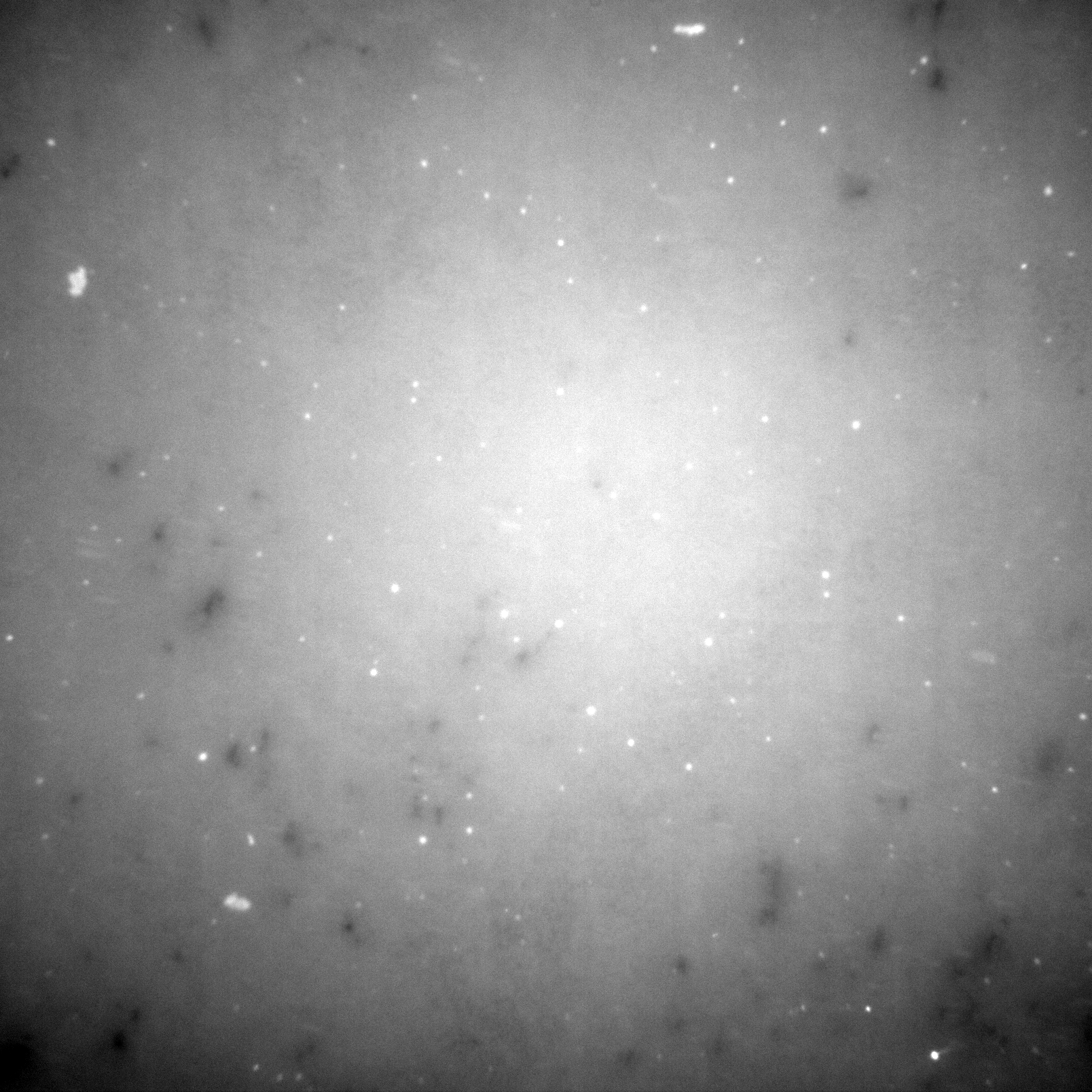

Supplement: Supplementary file 4 — Source data Fig. 2 [file 44318_2025_431_MOESM4_ESM.zip › Figure 2 copy/2A/NoRNA/INPUTDATA/1,6-HD 1.tif]

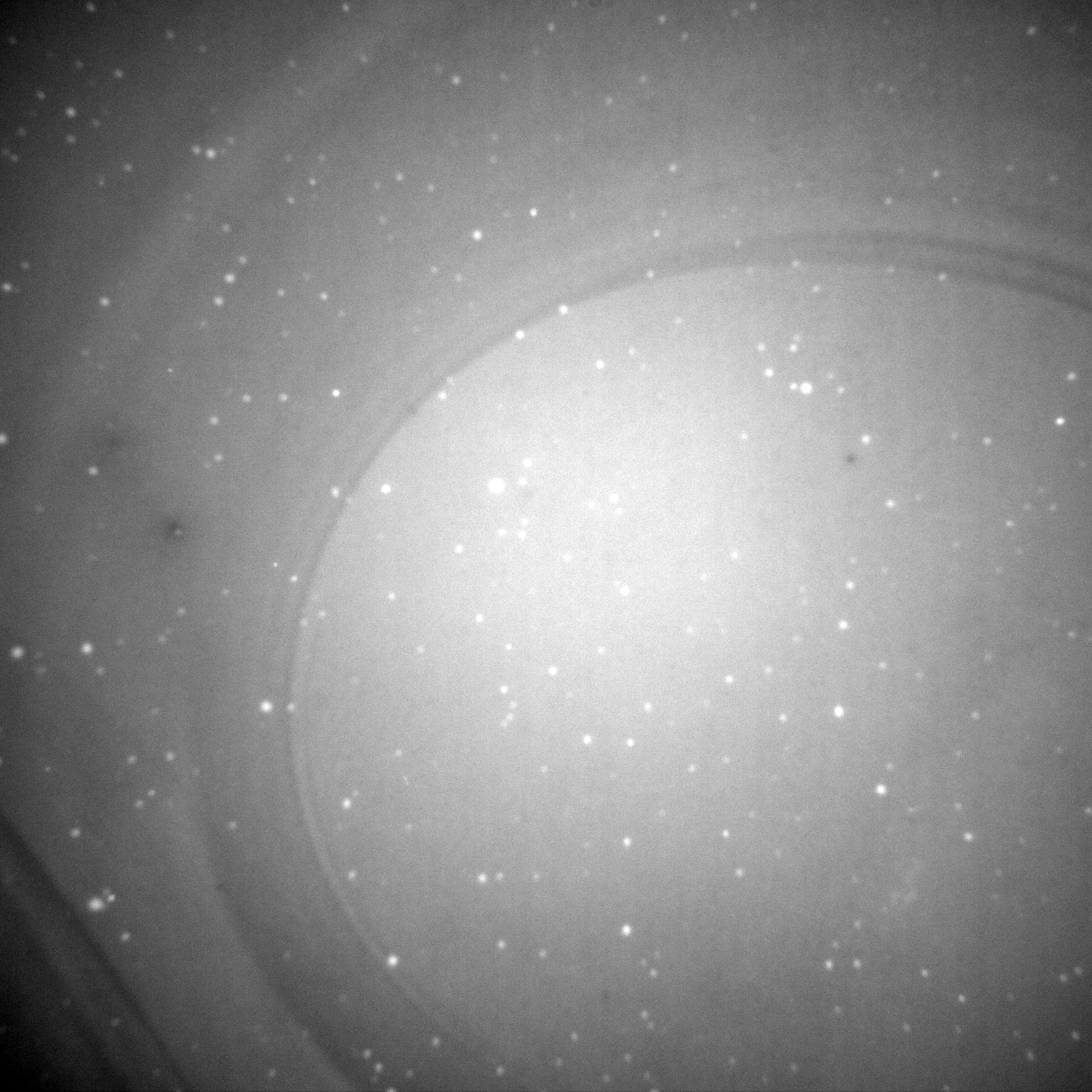

Supplement: Supplementary file 4 — Source data Fig. 2 [file 44318_2025_431_MOESM4_ESM.zip › Figure 2 copy/2A/NoRNA/INPUTDATA/1,6-HD 2.tif]

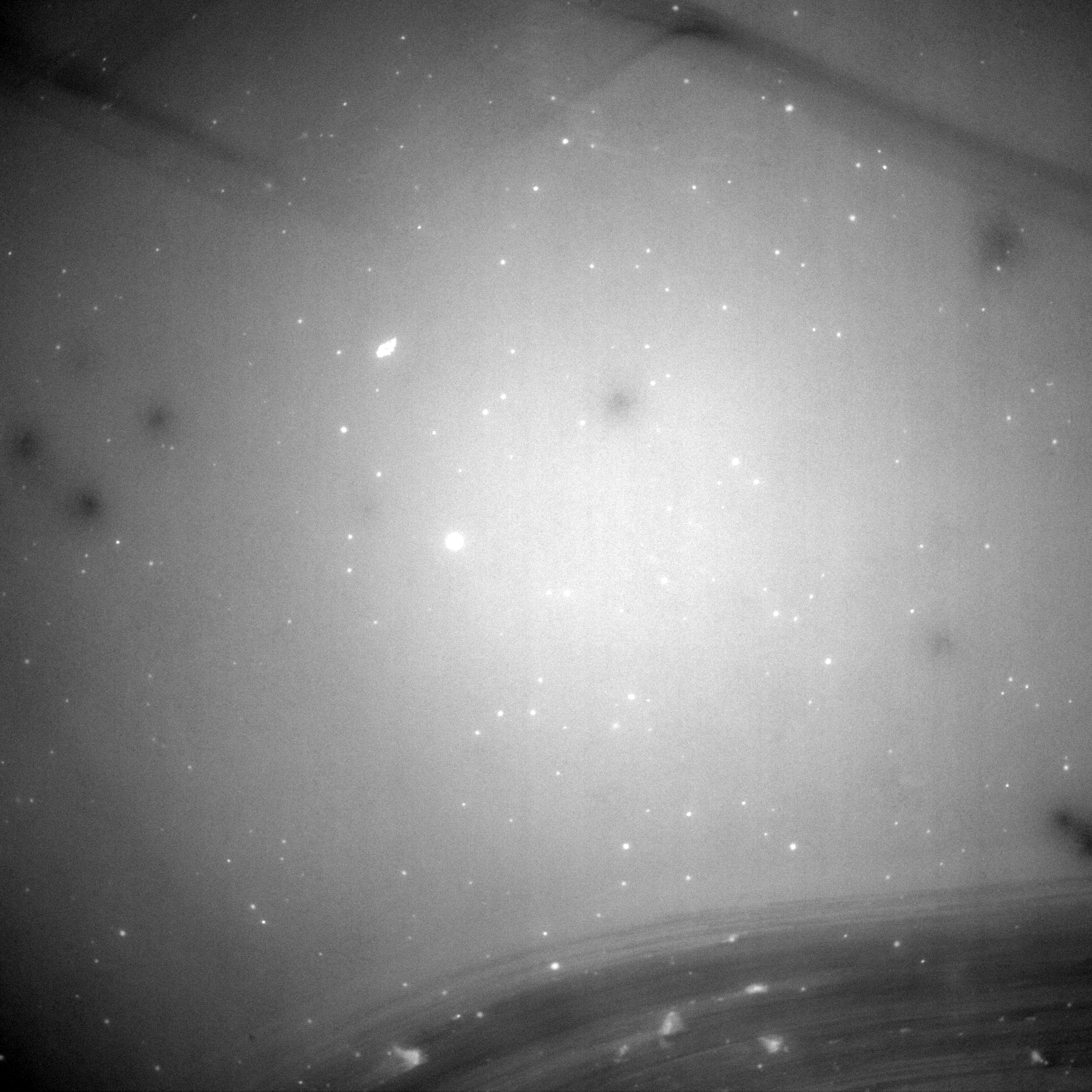

Supplement: Supplementary file 4 — Source data Fig. 2 [file 44318_2025_431_MOESM4_ESM.zip › Figure 2 copy/2A/NoRNA/INPUTDATA/1,6-HD 3.tif]

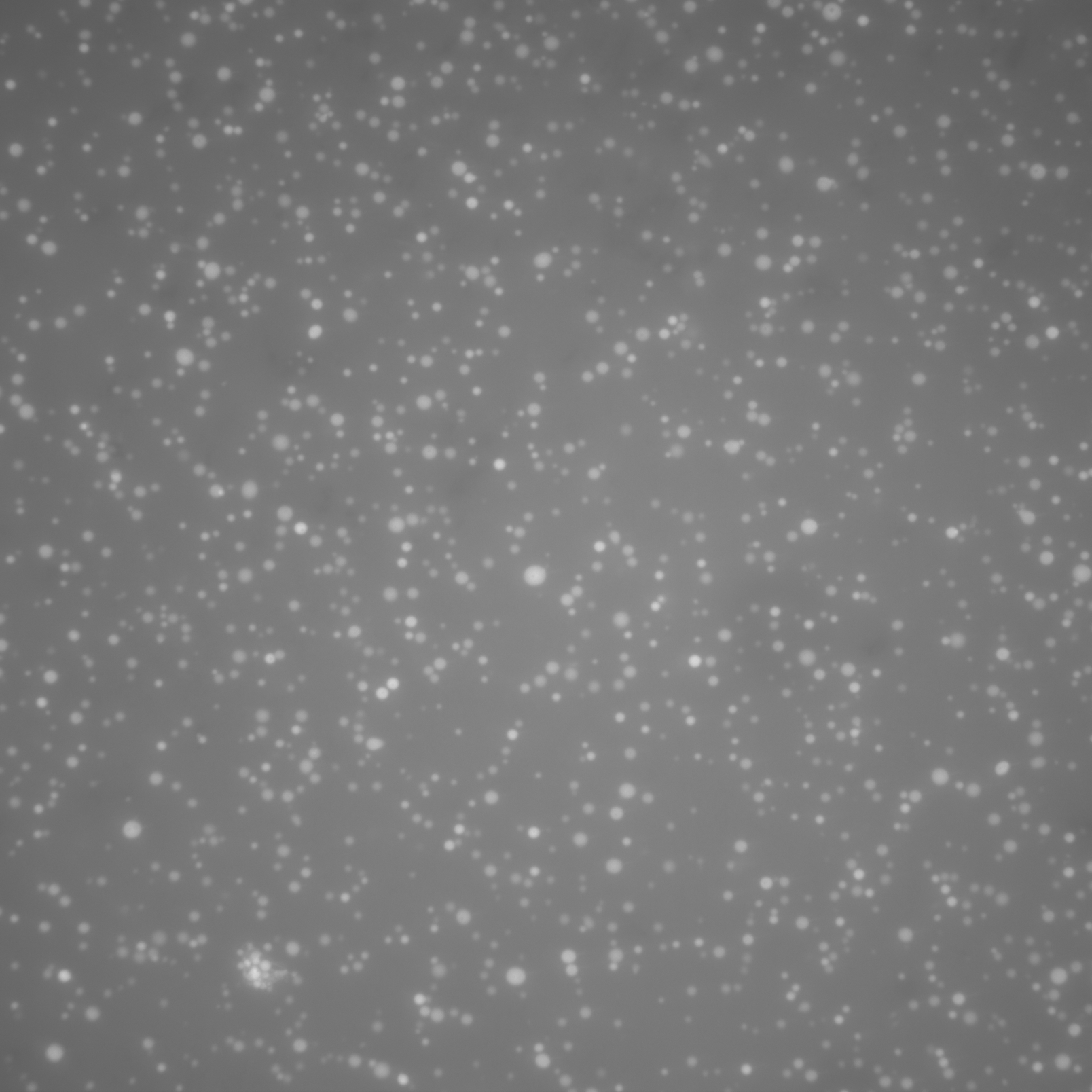

Supplement: Supplementary file 4 — Source data Fig. 2 [file 44318_2025_431_MOESM4_ESM.zip › Figure 2 copy/2A/NoRNA/INPUTDATA/no additive 4.tif]

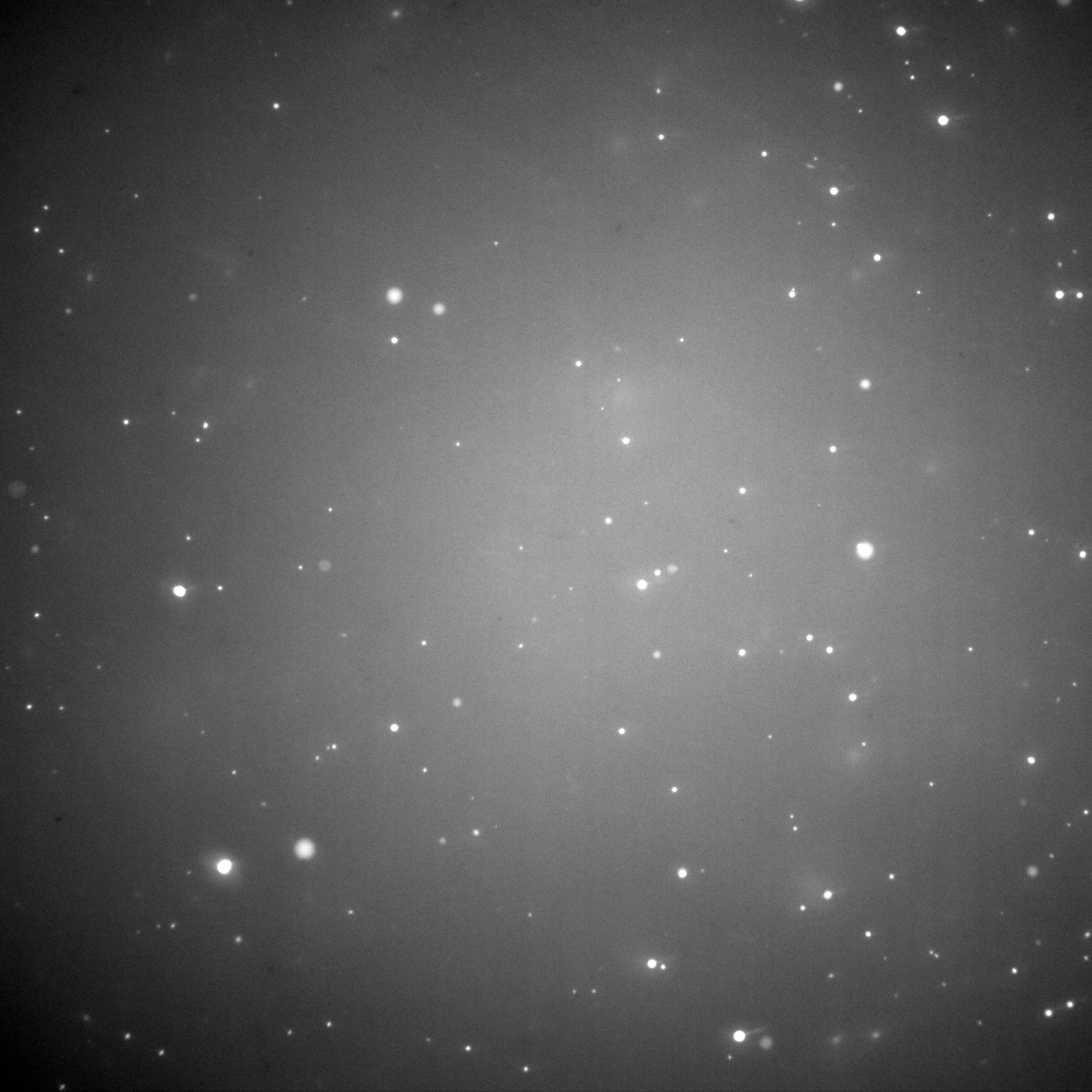

Supplement: Supplementary file 4 — Source data Fig. 2 [file 44318_2025_431_MOESM4_ESM.zip › Figure 2 copy/2A/NoRNA/INPUTDATA/1,2-HD 4.tif]

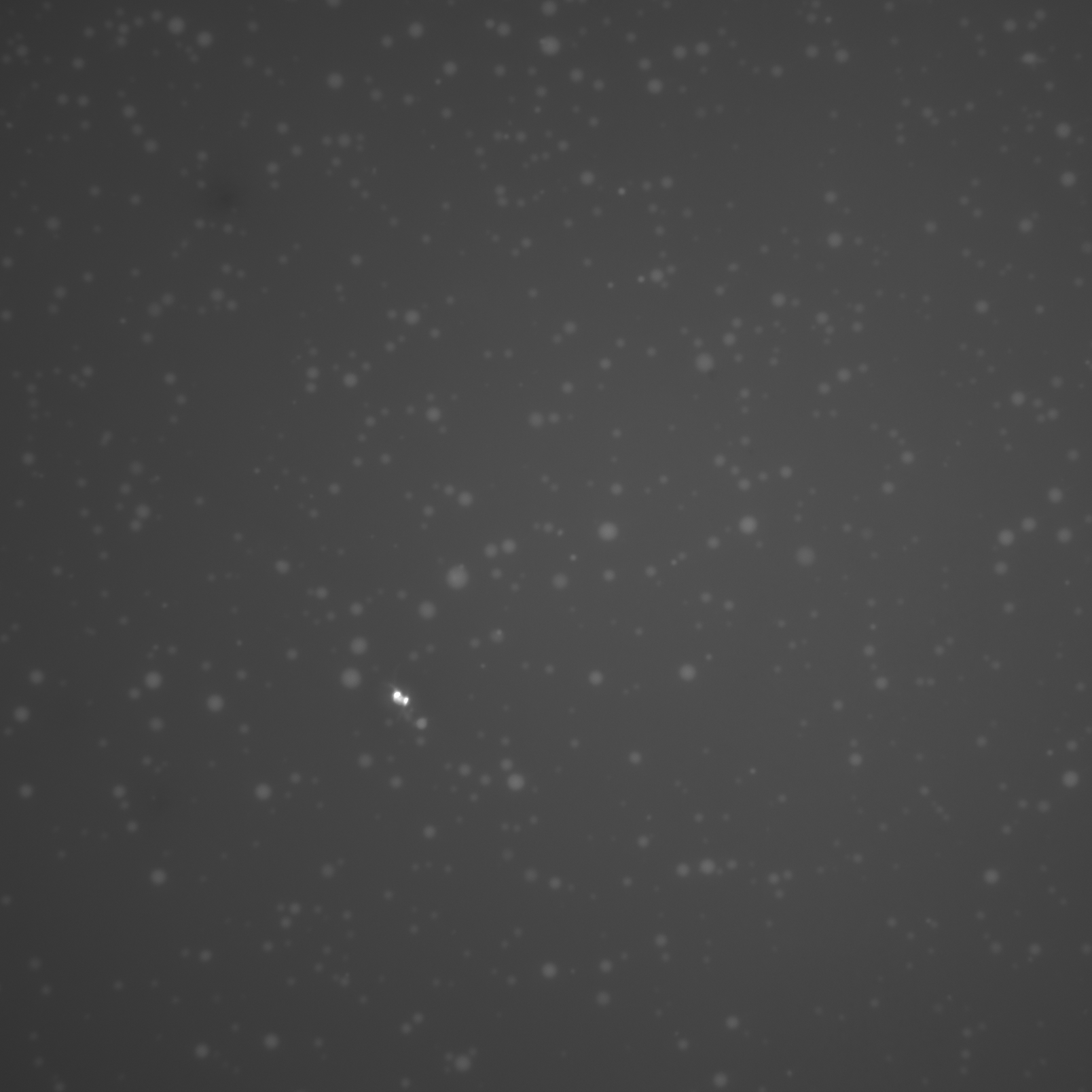

Supplement: Supplementary file 4 — Source data Fig. 2 [file 44318_2025_431_MOESM4_ESM.zip › Figure 2 copy/2A/NoRNA/INPUTDATA/1,4-BD 1.tif]

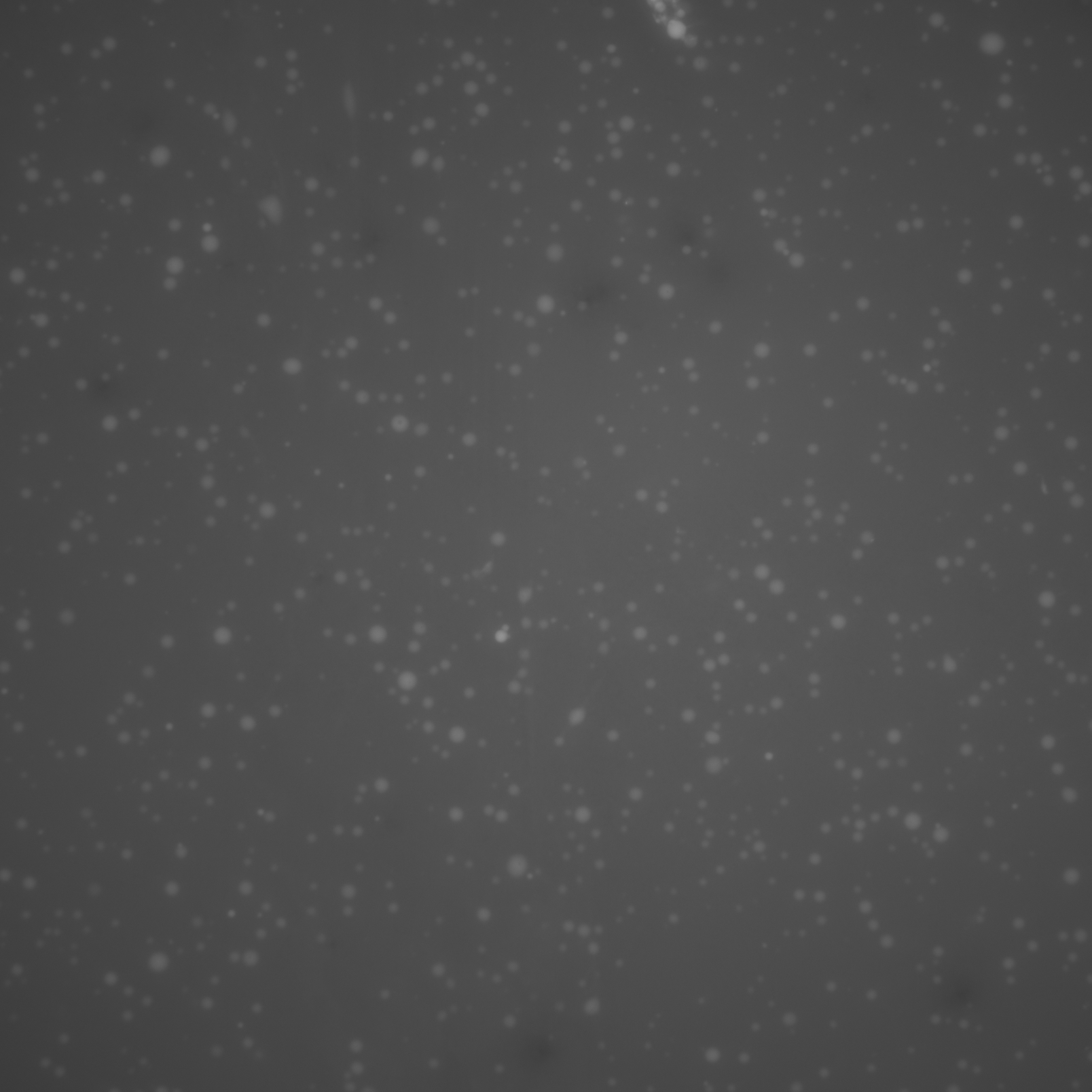

Supplement: Supplementary file 4 — Source data Fig. 2 [file 44318_2025_431_MOESM4_ESM.zip › Figure 2 copy/2A/NoRNA/INPUTDATA/1,4-BD 3.tif]

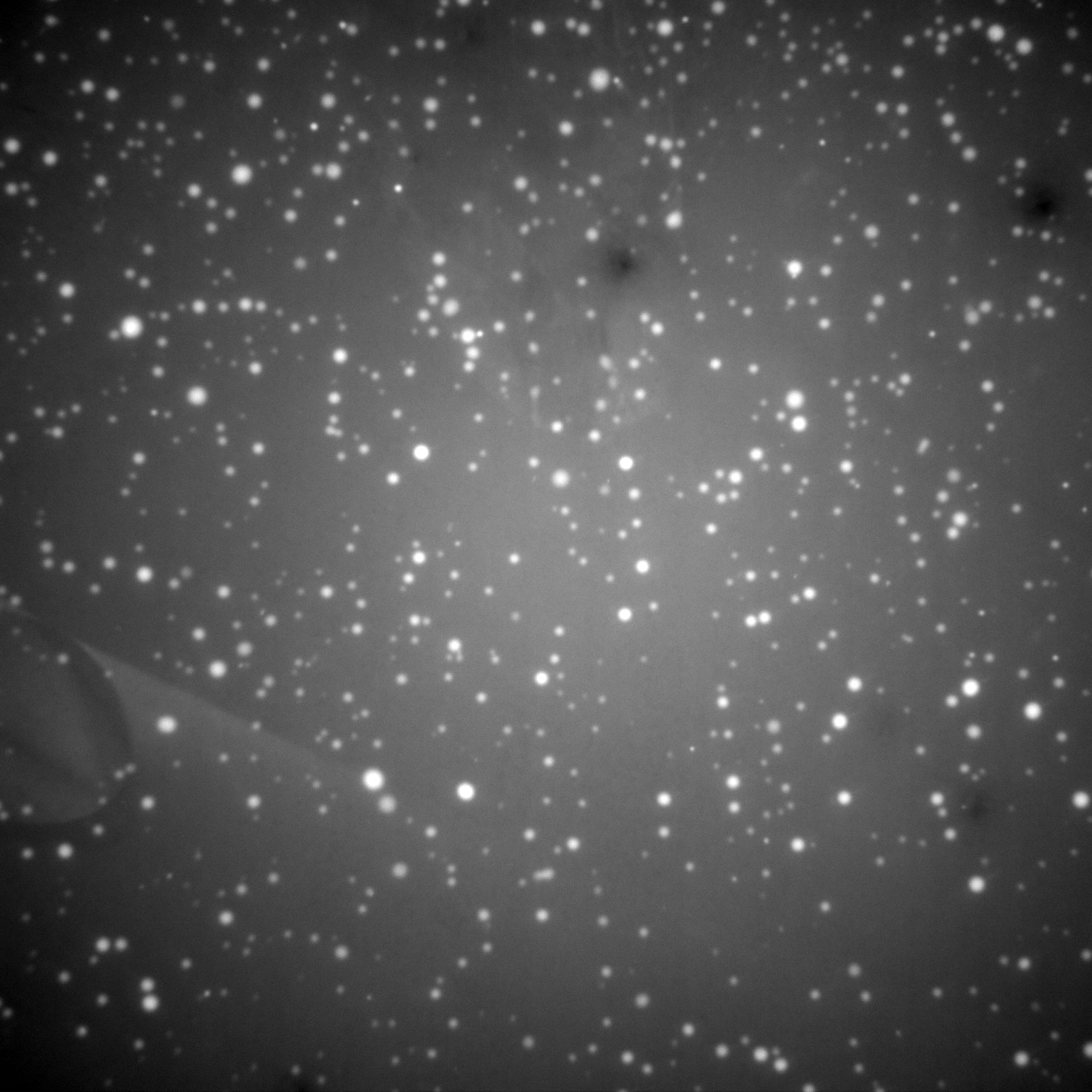

Supplement: Supplementary file 4 — Source data Fig. 2 [file 44318_2025_431_MOESM4_ESM.zip › Figure 2 copy/2A/NoRNA/INPUTDATA/1,4-BD 2.tif]

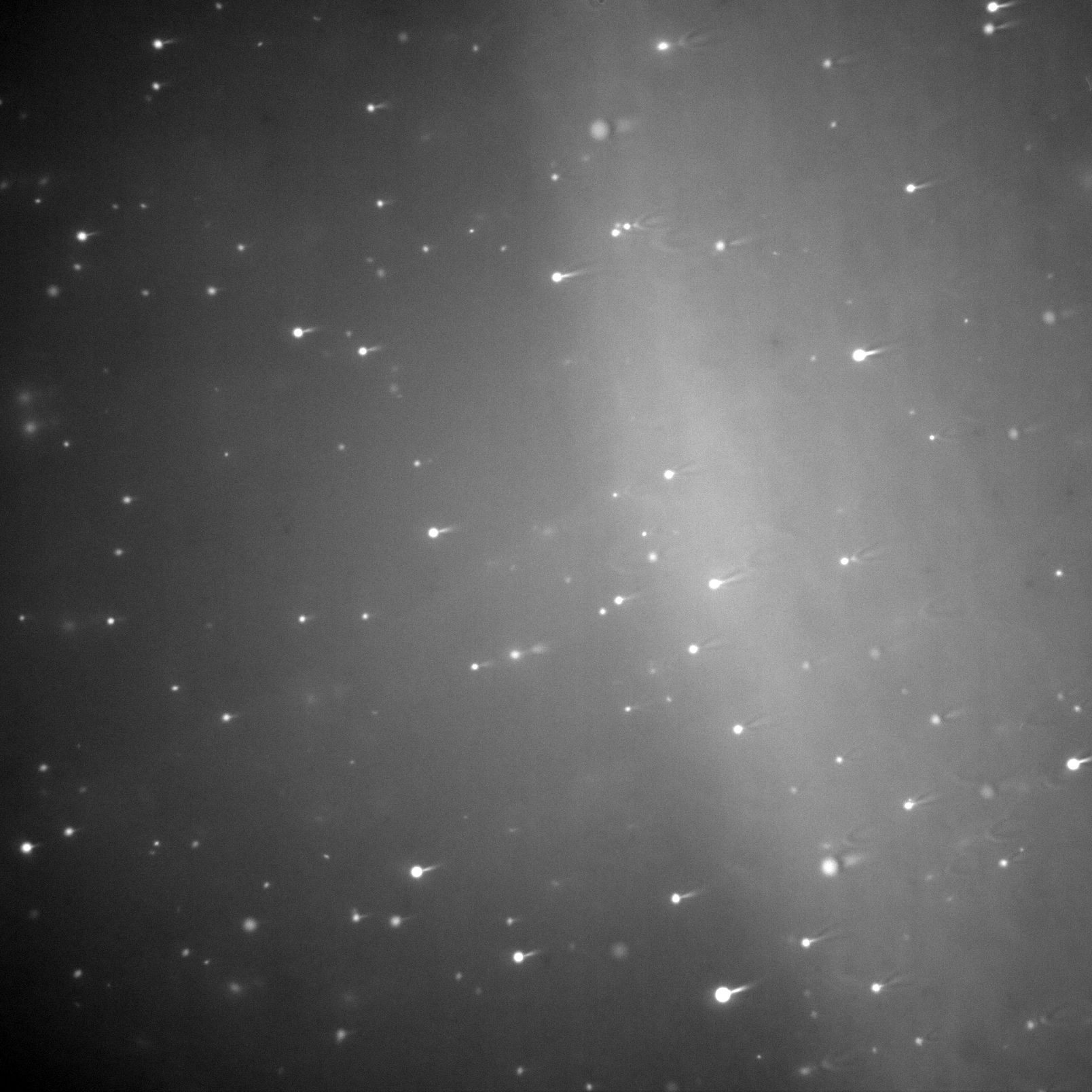

Supplement: Supplementary file 4 — Source data Fig. 2 [file 44318_2025_431_MOESM4_ESM.zip › Figure 2 copy/2A/NoRNA/INPUTDATA/1,2-HD 2.tif]

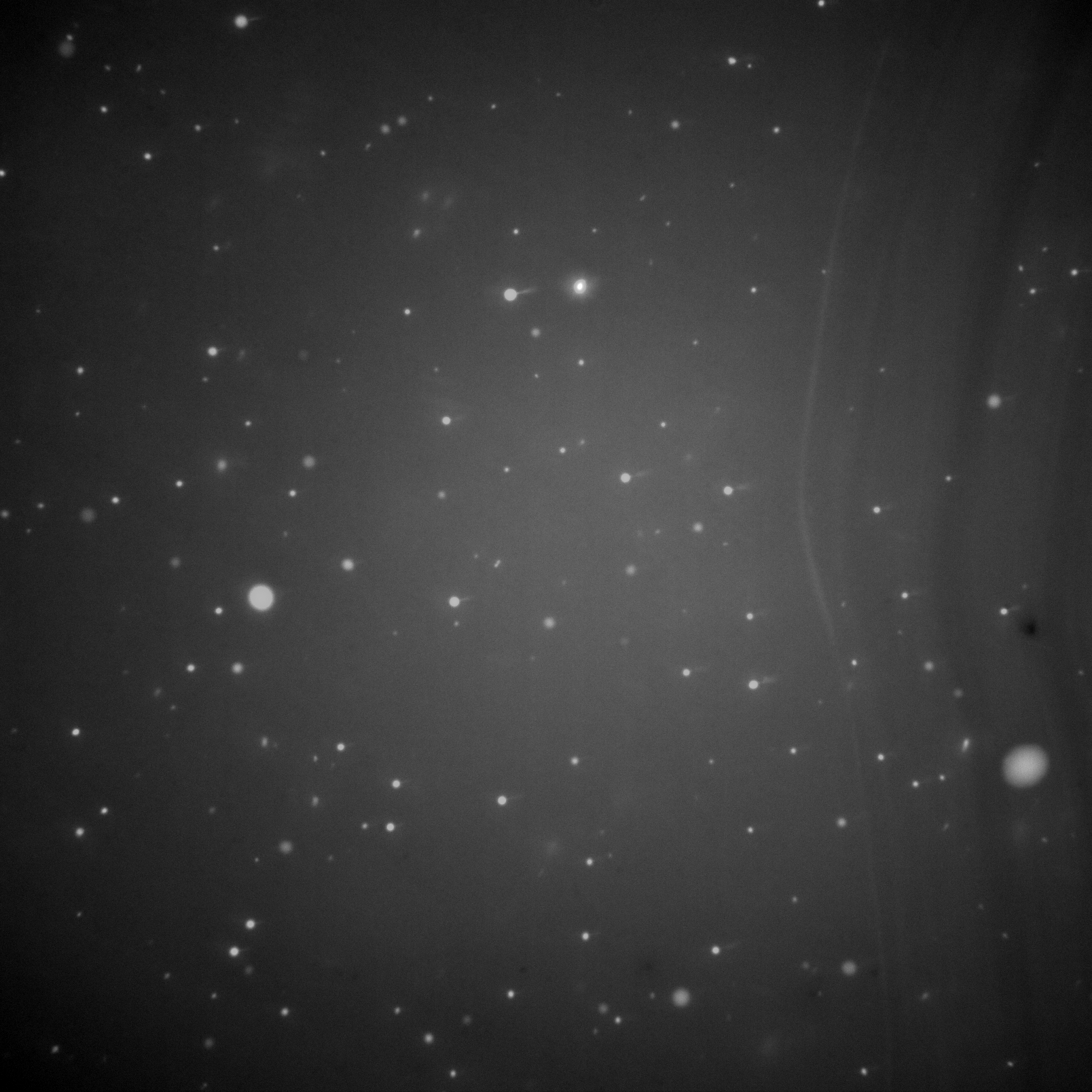

Supplement: Supplementary file 4 — Source data Fig. 2 [file 44318_2025_431_MOESM4_ESM.zip › Figure 2 copy/2A/NoRNA/INPUTDATA/1,2-HD 3.tif]

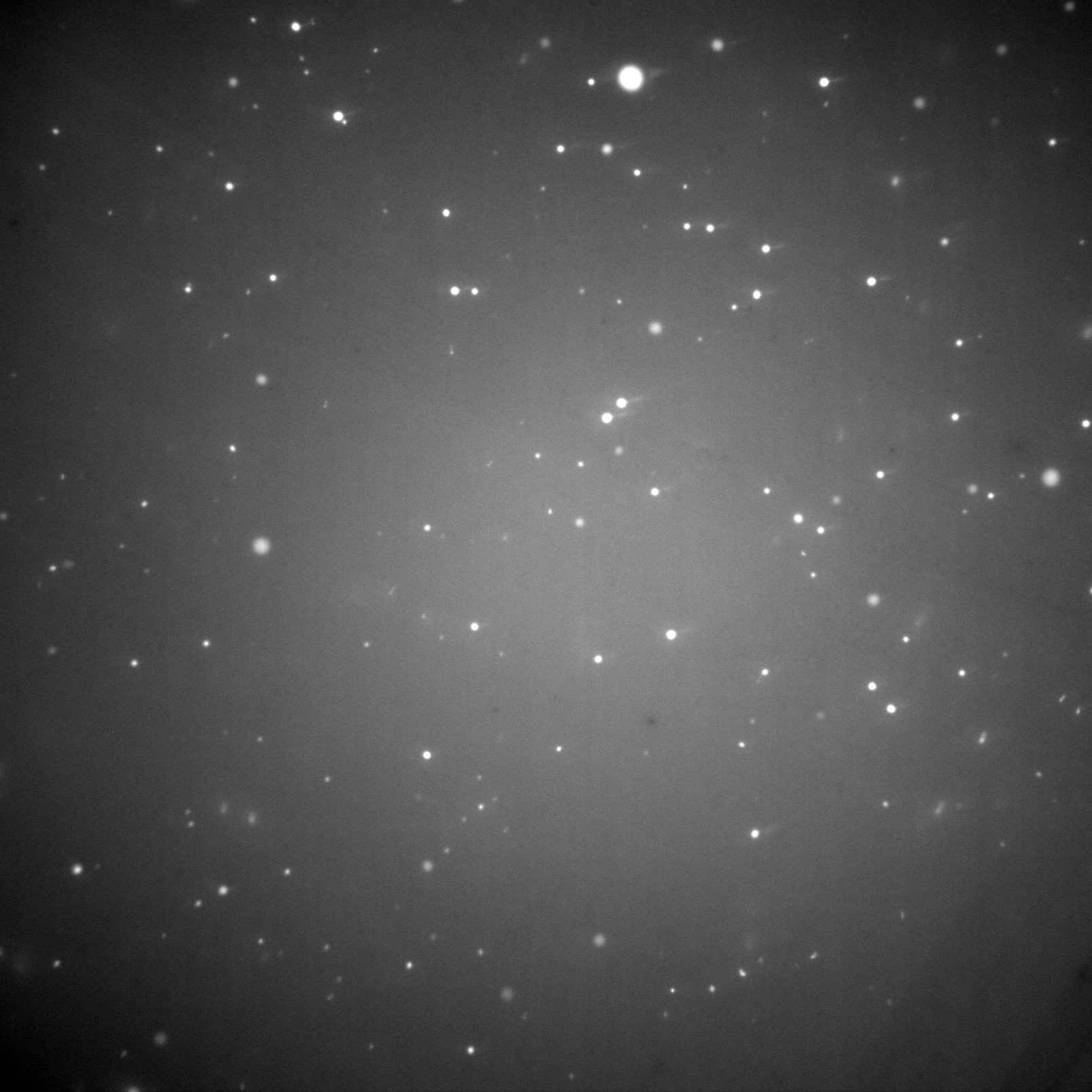

Supplement: Supplementary file 4 — Source data Fig. 2 [file 44318_2025_431_MOESM4_ESM.zip › Figure 2 copy/2A/NoRNA/INPUTDATA/1,2-HD 1.tif]

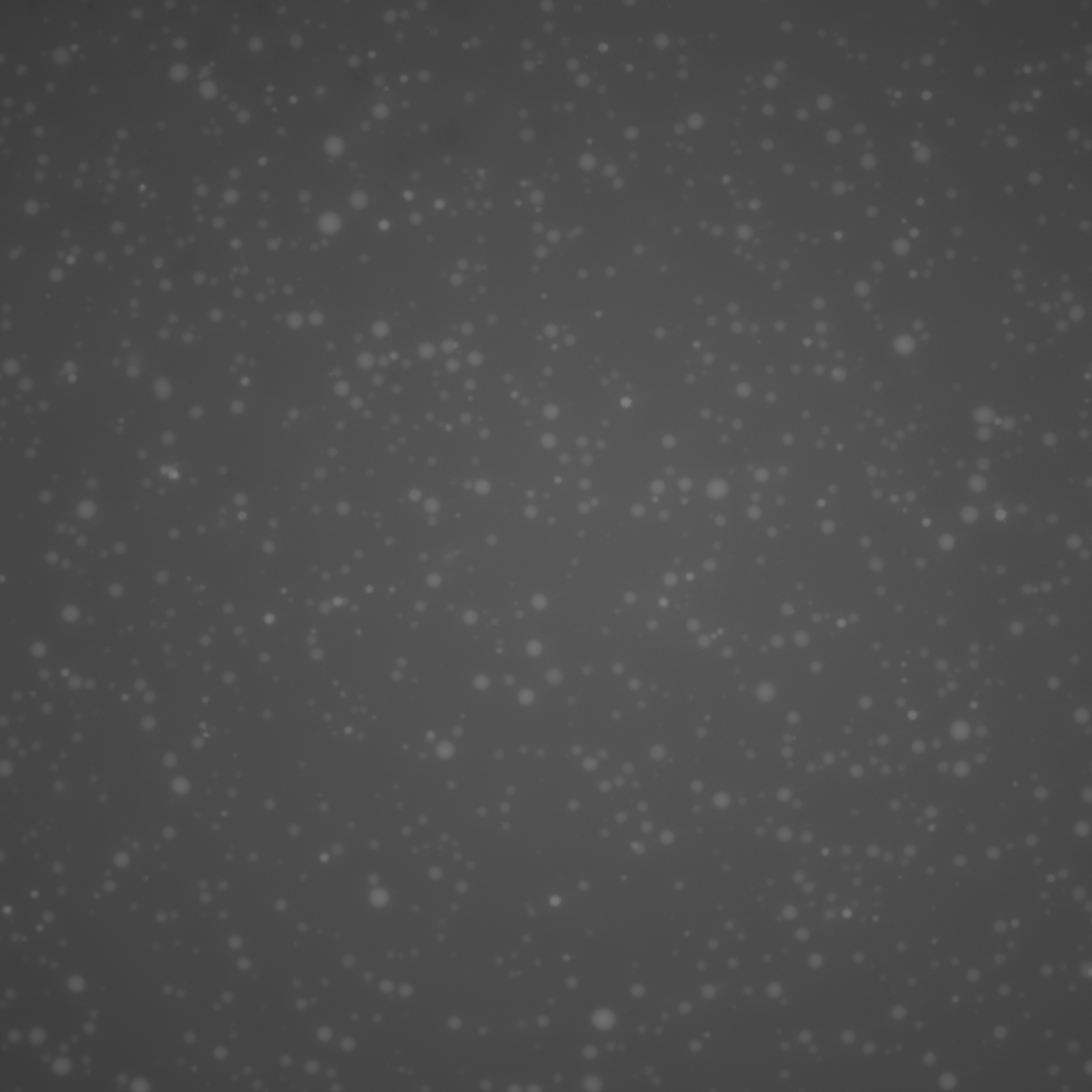

Supplement: Supplementary file 4 — Source data Fig. 2 [file 44318_2025_431_MOESM4_ESM.zip › Figure 2 copy/2A/NoRNA/INPUTDATA/1,4-BD 4.tif]

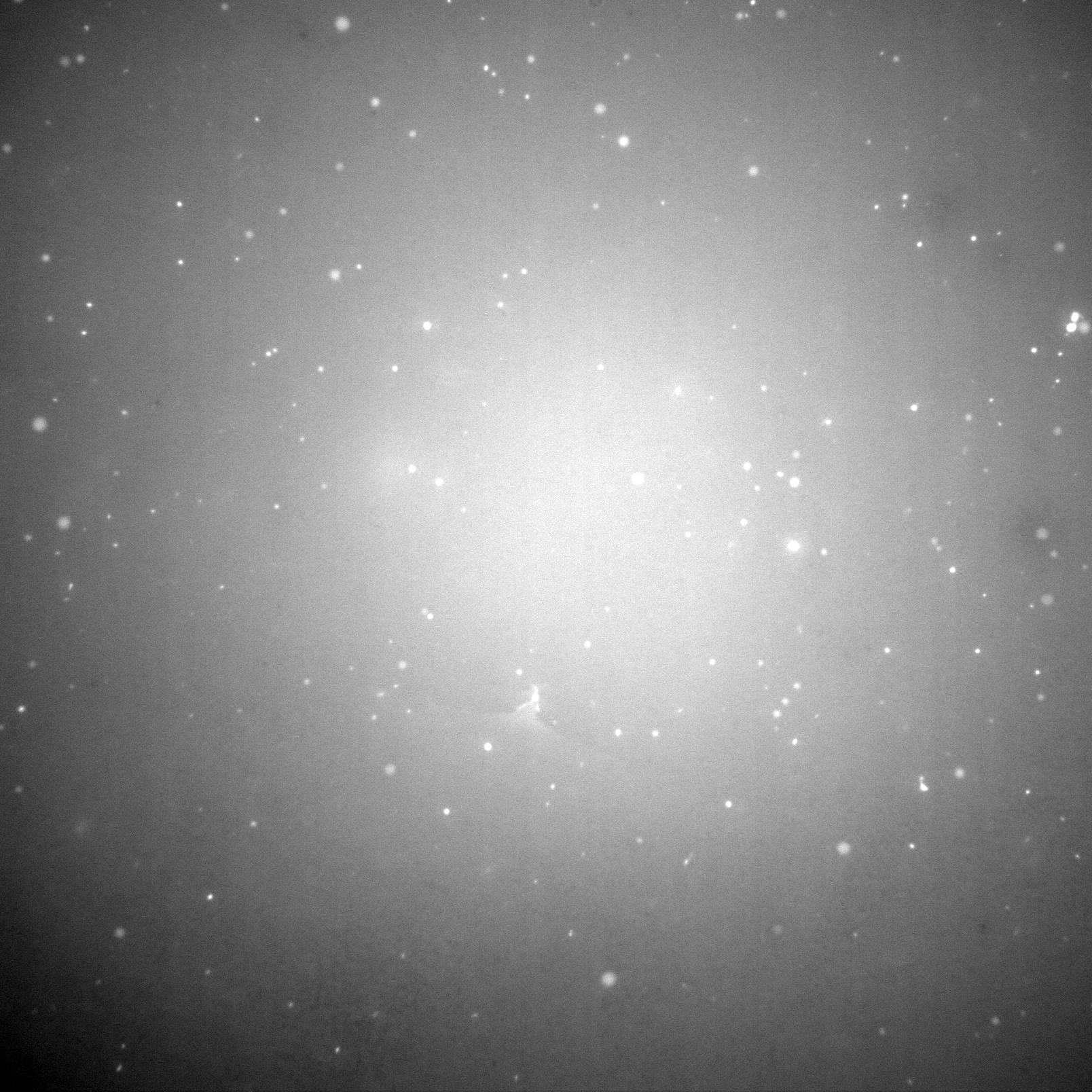

Supplement: Supplementary file 4 — Source data Fig. 2 [file 44318_2025_431_MOESM4_ESM.zip › Figure 2 copy/2A/NoRNA/INPUTDATA/1,2-CHD 1.tif]

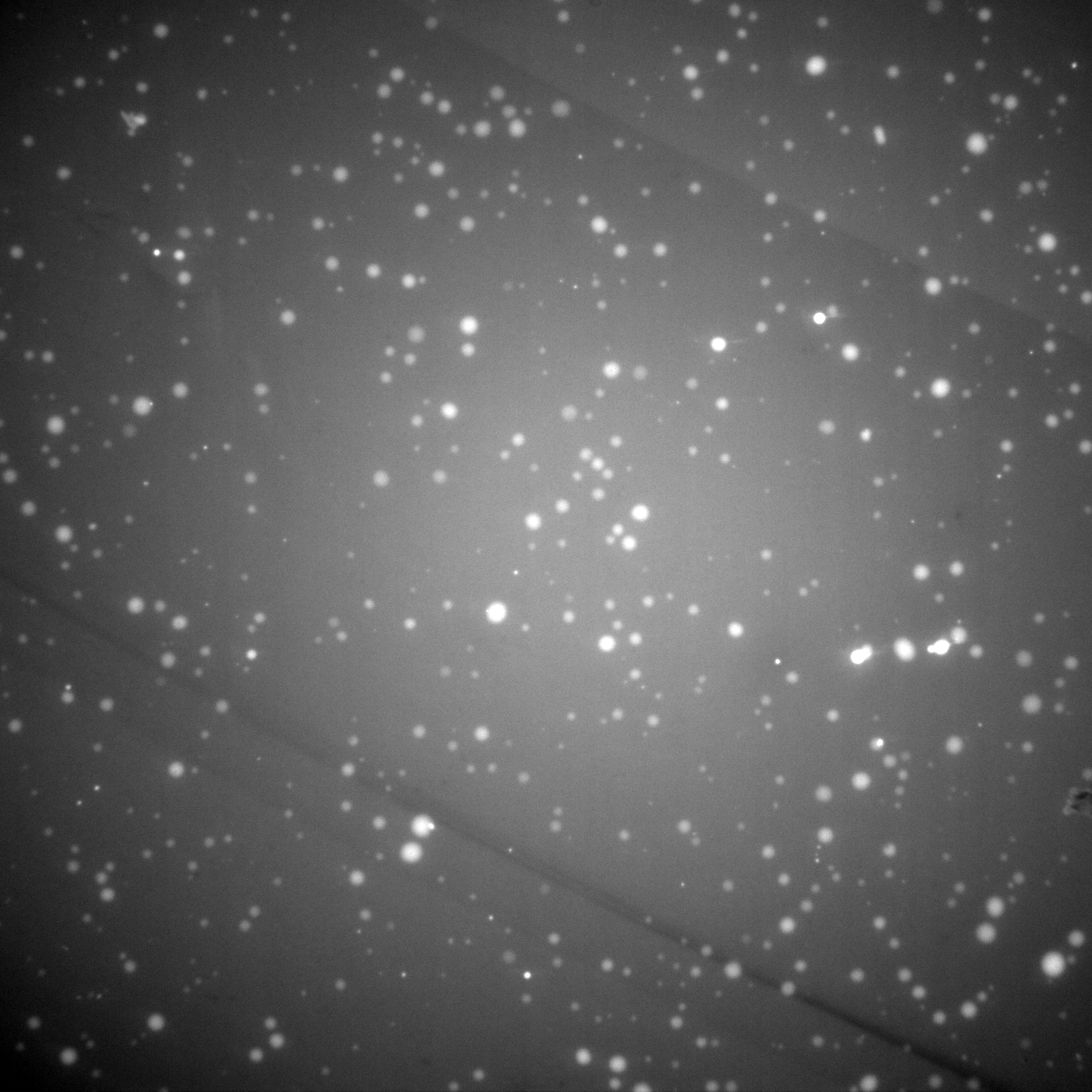

Supplement: Supplementary file 4 — Source data Fig. 2 [file 44318_2025_431_MOESM4_ESM.zip › Figure 2 copy/2A/NoRNA/INPUTDATA/1,5-PD 3.tif]

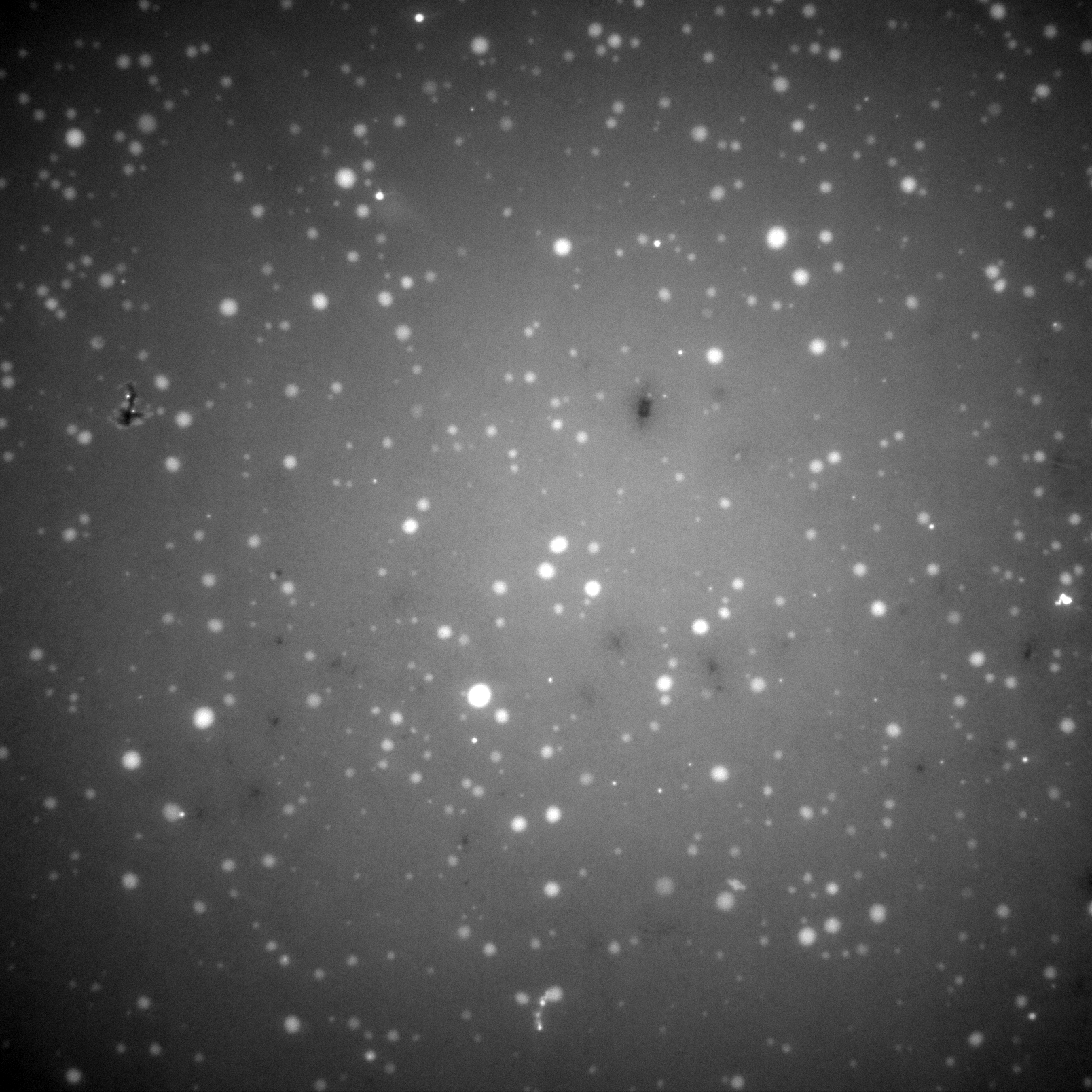

Supplement: Supplementary file 4 — Source data Fig. 2 [file 44318_2025_431_MOESM4_ESM.zip › Figure 2 copy/2A/NoRNA/INPUTDATA/1,5-PD 2.tif]

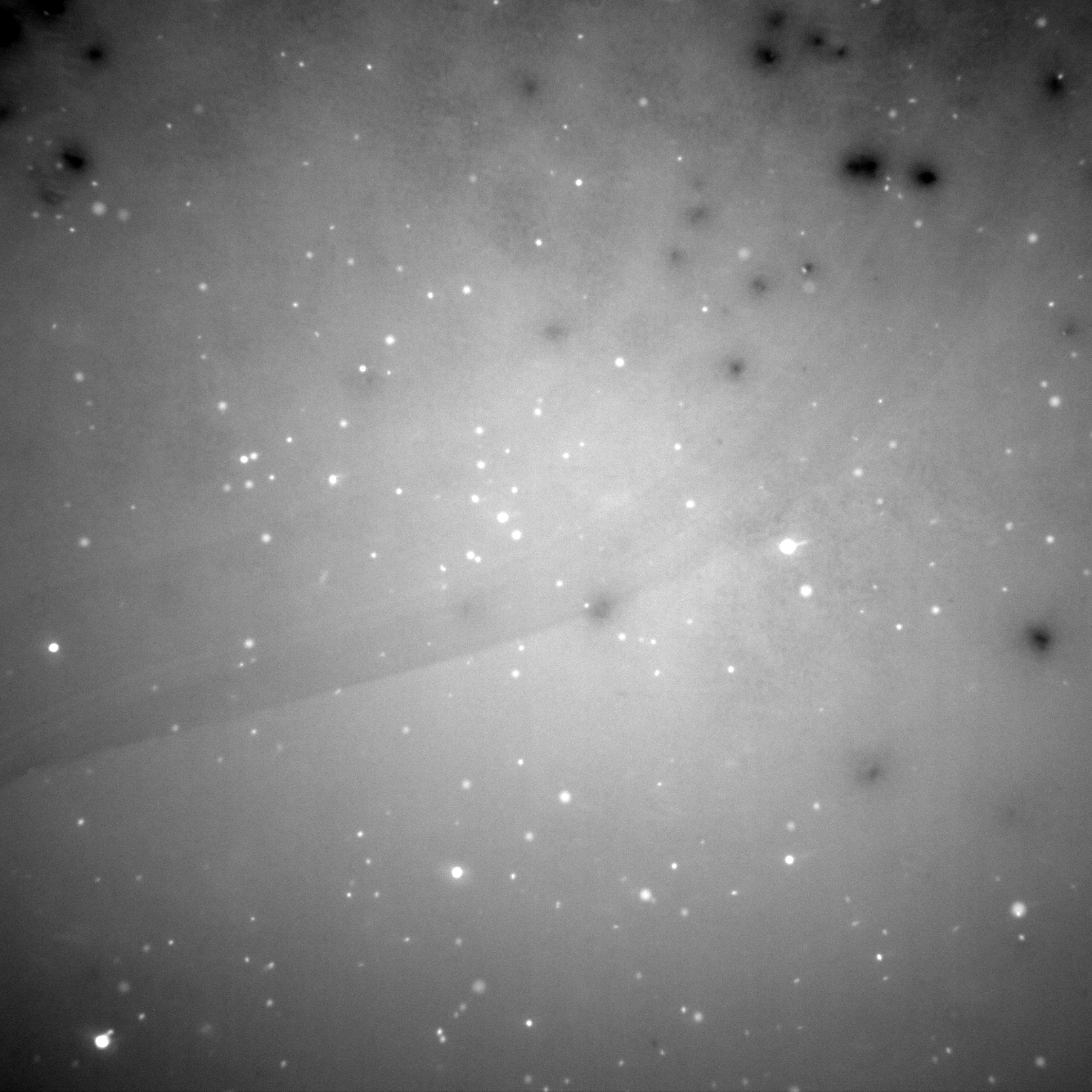

Supplement: Supplementary file 4 — Source data Fig. 2 [file 44318_2025_431_MOESM4_ESM.zip › Figure 2 copy/2A/NoRNA/INPUTDATA/1,2-CHD 2.tif]

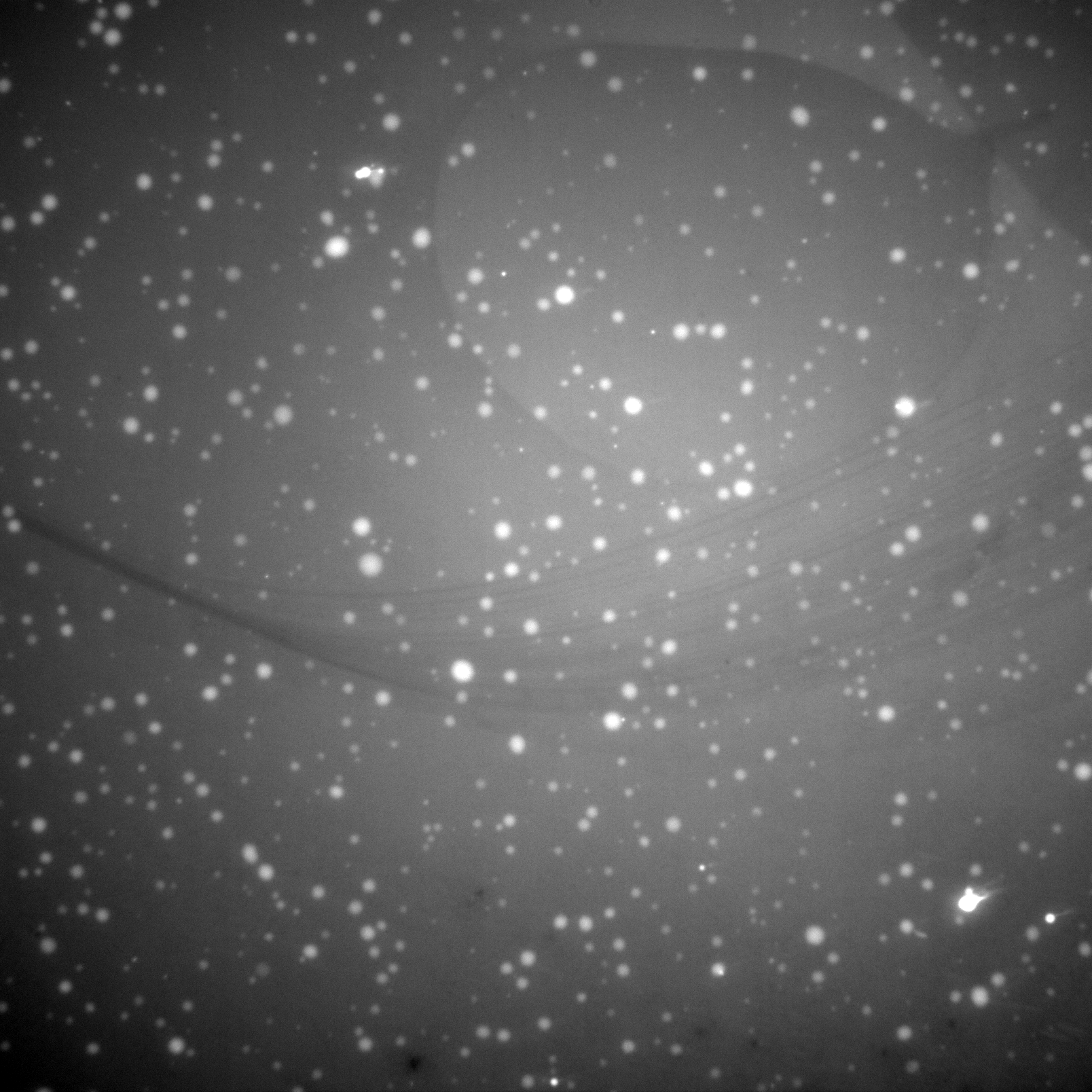

Supplement: Supplementary file 4 — Source data Fig. 2 [file 44318_2025_431_MOESM4_ESM.zip › Figure 2 copy/2A/NoRNA/INPUTDATA/1,5-PD 1.tif]

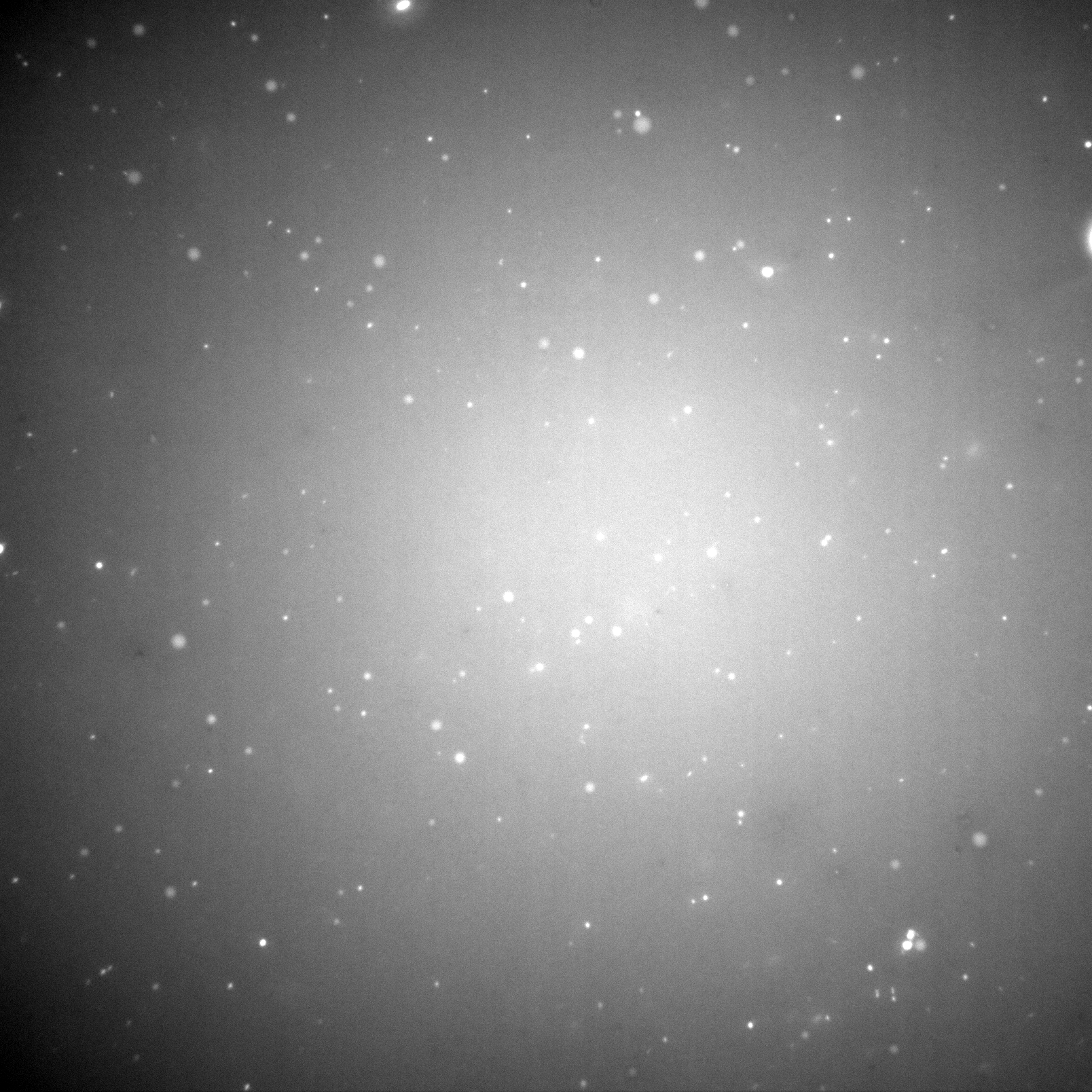

Supplement: Supplementary file 4 — Source data Fig. 2 [file 44318_2025_431_MOESM4_ESM.zip › Figure 2 copy/2A/NoRNA/INPUTDATA/1,2-CHD 3.tif]

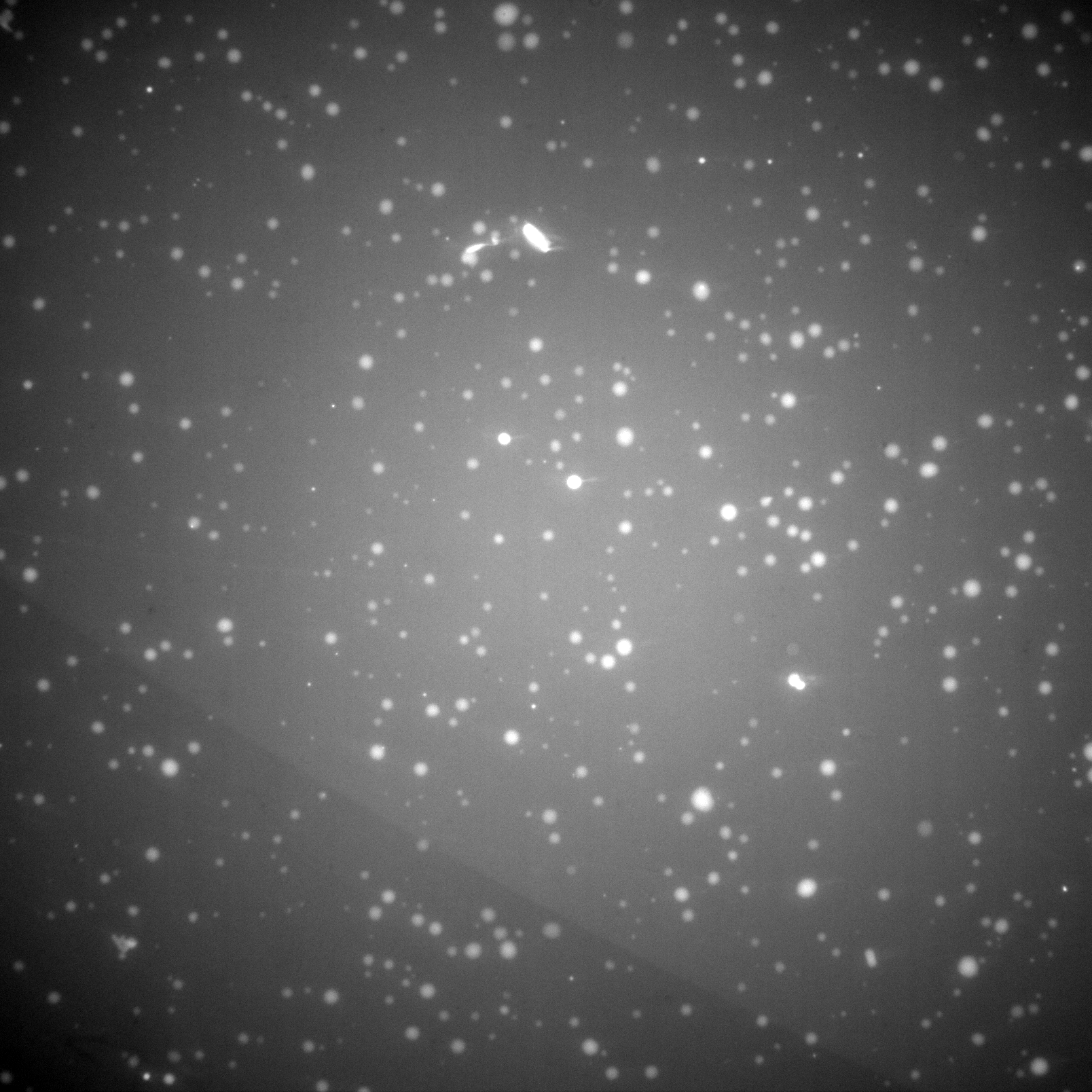

Supplement: Supplementary file 4 — Source data Fig. 2 [file 44318_2025_431_MOESM4_ESM.zip › Figure 2 copy/2A/NoRNA/INPUTDATA/1,5-PD 4.tif]

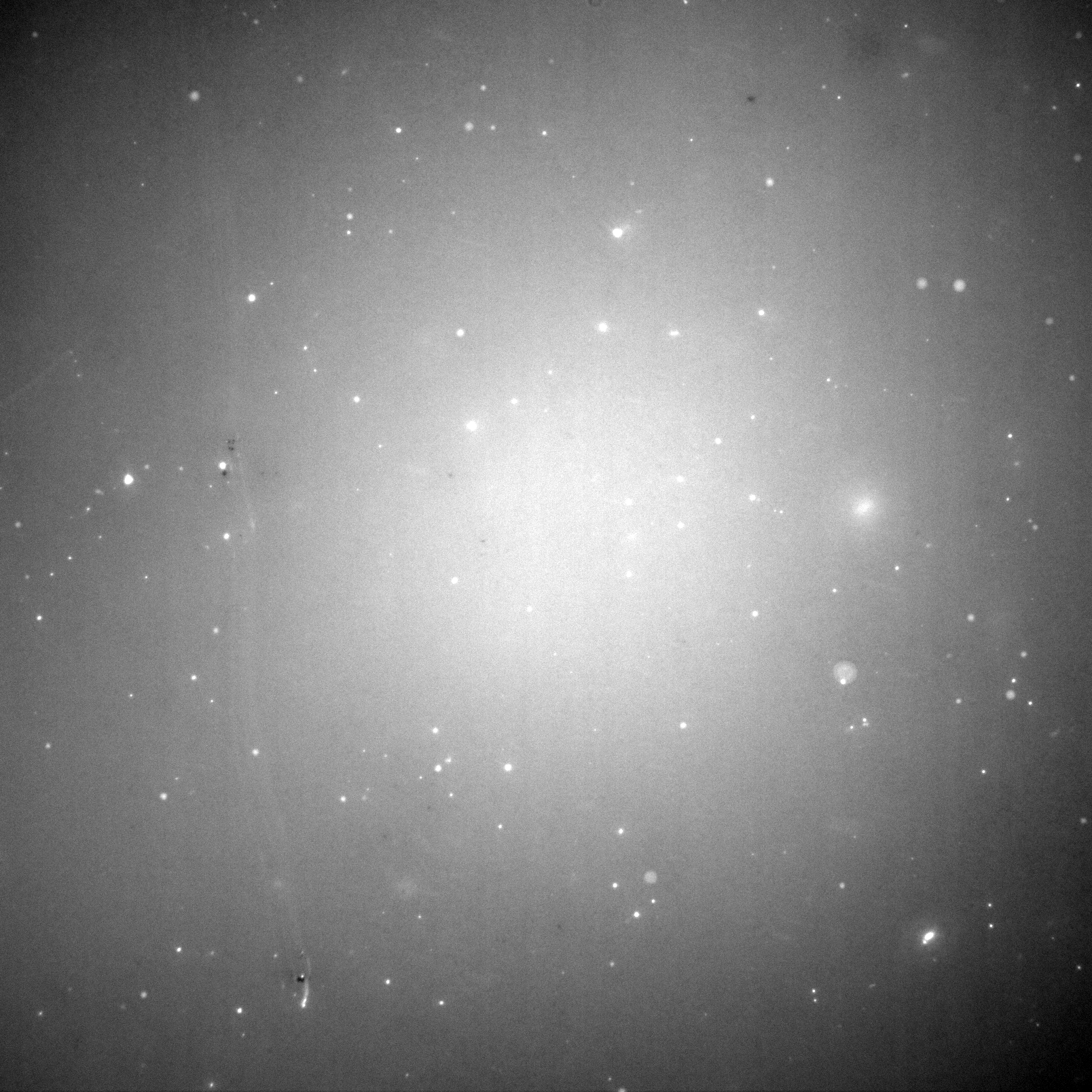

Supplement: Supplementary file 4 — Source data Fig. 2 [file 44318_2025_431_MOESM4_ESM.zip › Figure 2 copy/2A/NoRNA/INPUTDATA/1,2-CHD 4.tif]

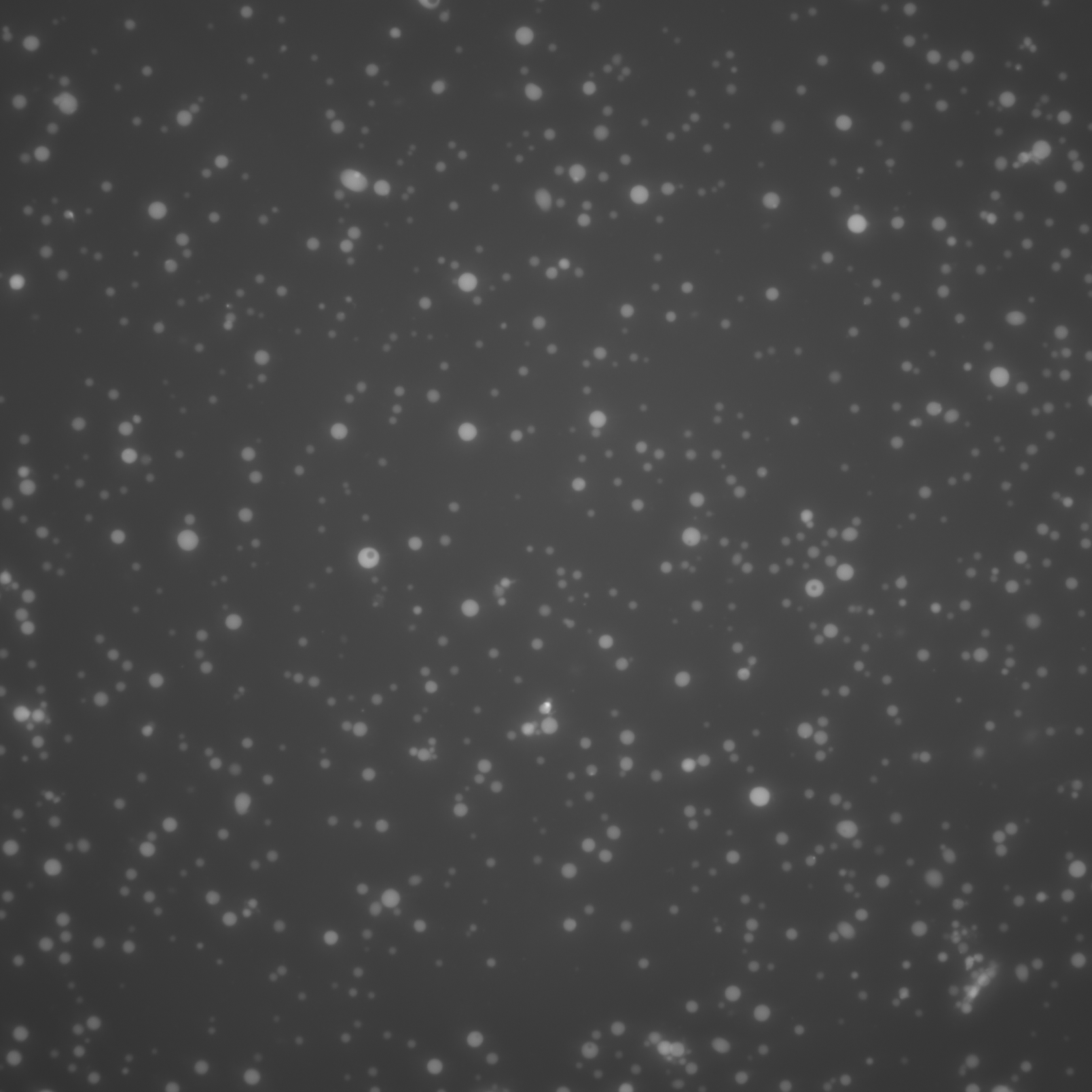

Supplement: Supplementary file 4 — Source data Fig. 2 [file 44318_2025_431_MOESM4_ESM.zip › Figure 2 copy/2A/polyU_RNA/InputData/12CHD/1,2-CHD 1.tif]

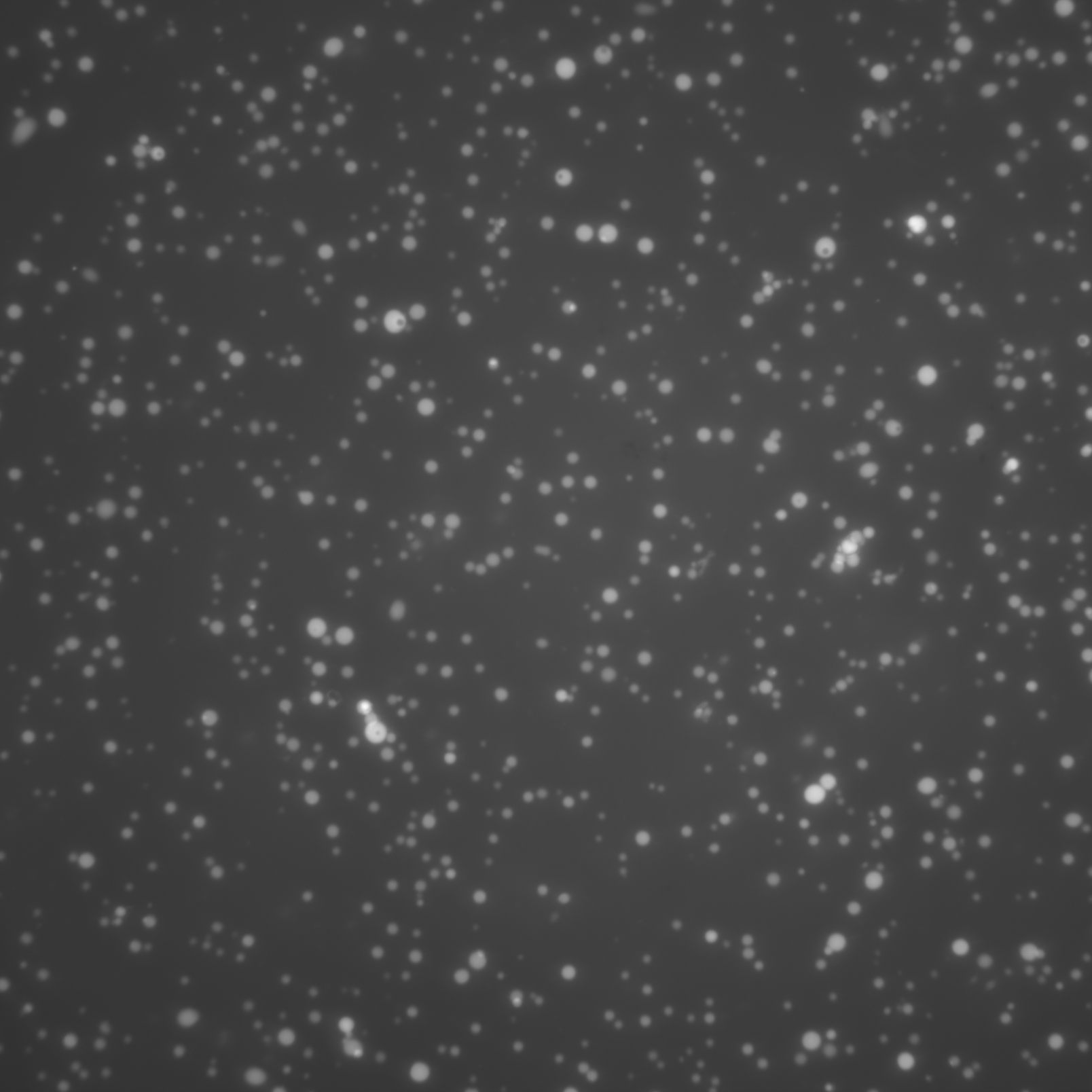

Supplement: Supplementary file 4 — Source data Fig. 2 [file 44318_2025_431_MOESM4_ESM.zip › Figure 2 copy/2A/polyU_RNA/InputData/12CHD/1,2-CHD 2.tif]

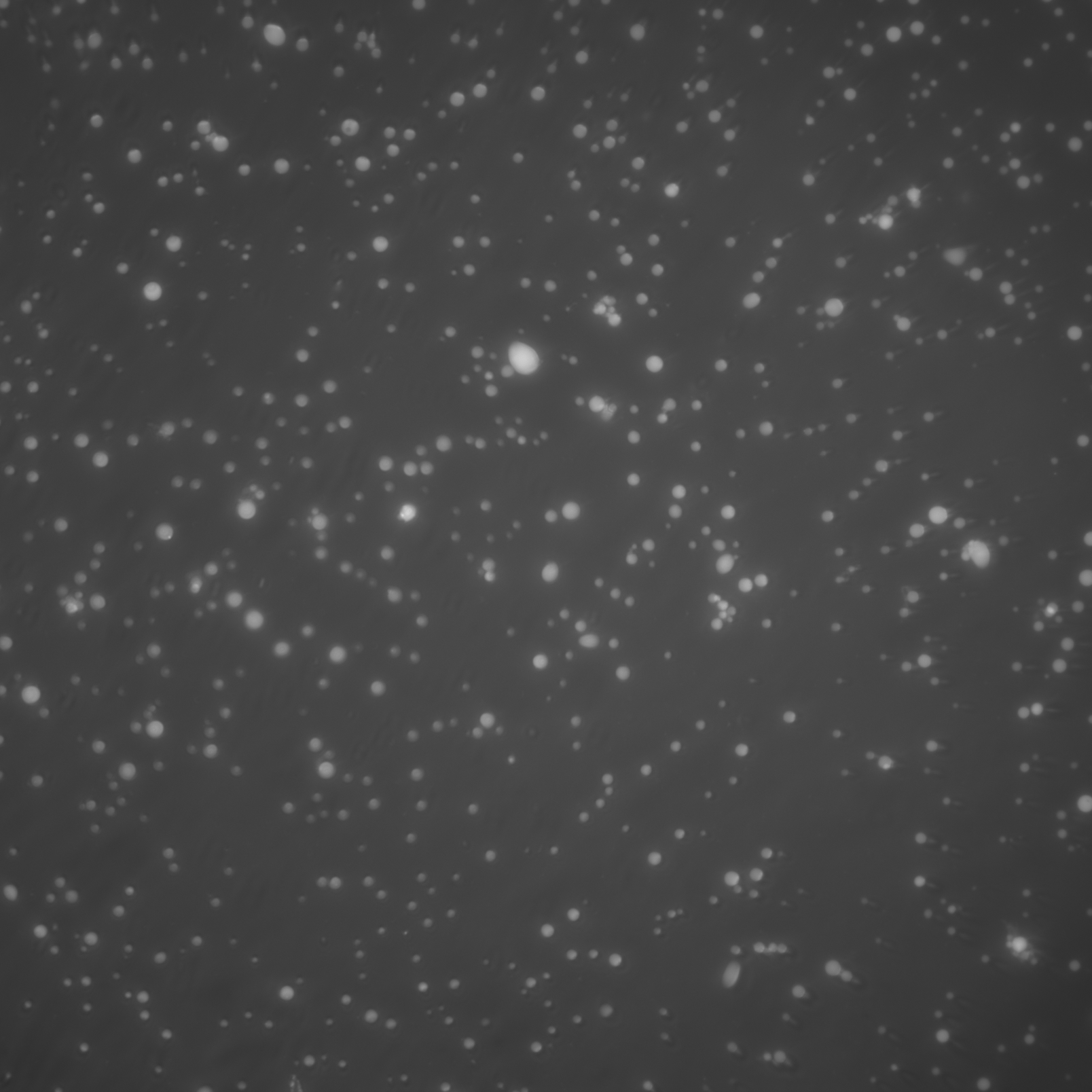

Supplement: Supplementary file 4 — Source data Fig. 2 [file 44318_2025_431_MOESM4_ESM.zip › Figure 2 copy/2A/polyU_RNA/InputData/12CHD/1,2-CHD 3.tif]

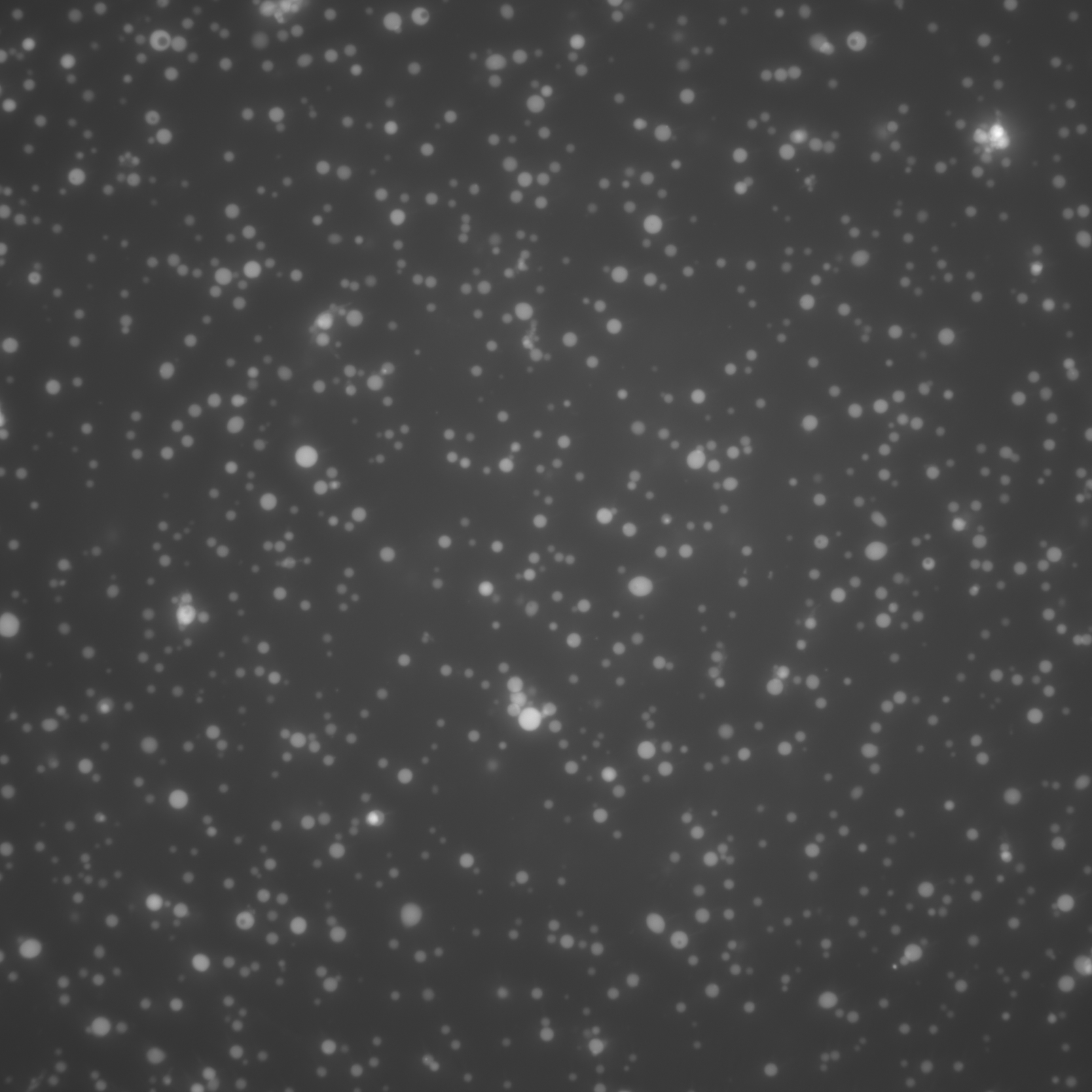

Supplement: Supplementary file 4 — Source data Fig. 2 [file 44318_2025_431_MOESM4_ESM.zip › Figure 2 copy/2A/polyU_RNA/InputData/12CHD/1,2-CHD 4.tif]

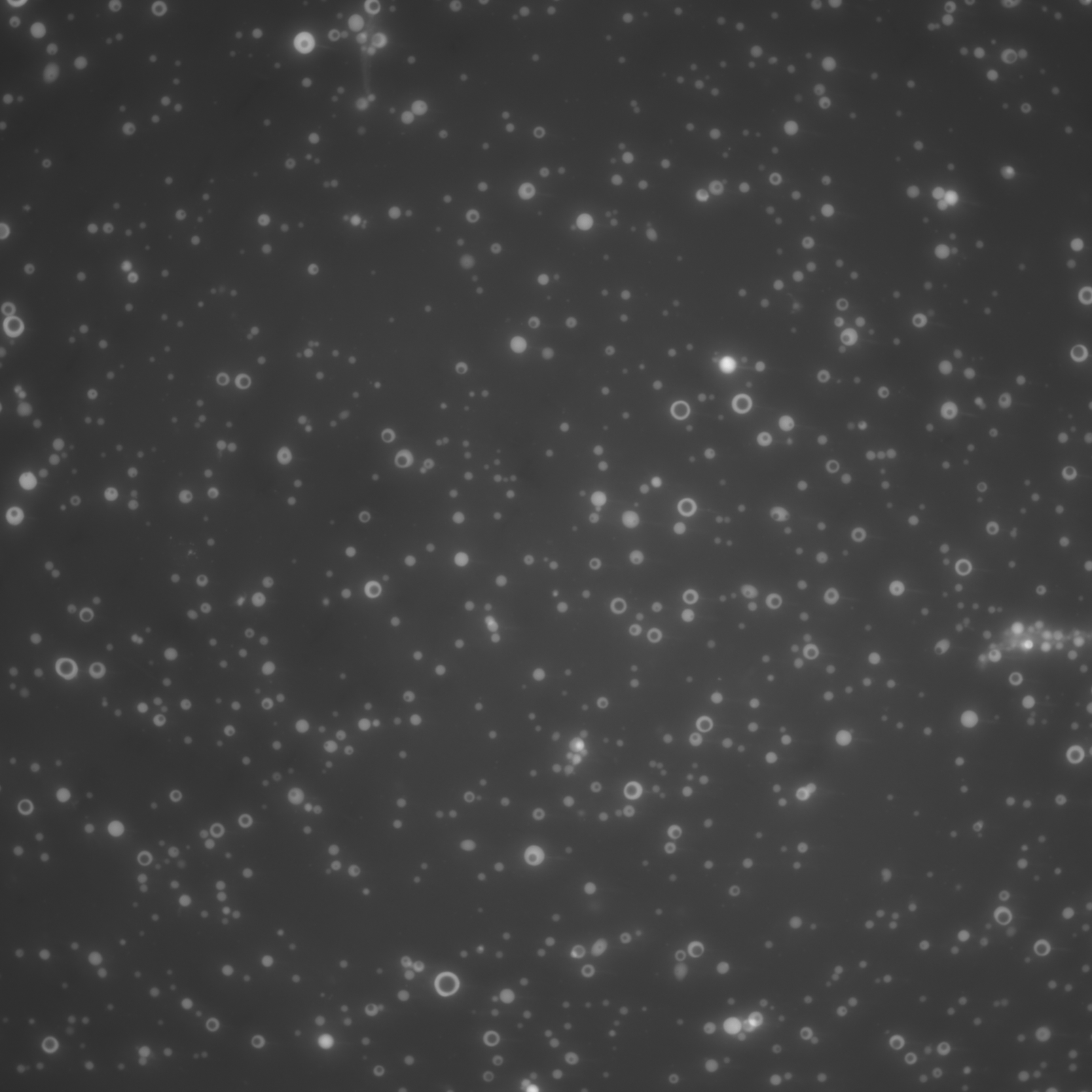

Supplement: Supplementary file 4 — Source data Fig. 2 [file 44318_2025_431_MOESM4_ESM.zip › Figure 2 copy/2A/polyU_RNA/InputData/25HD/2,5-HD 4.tif]

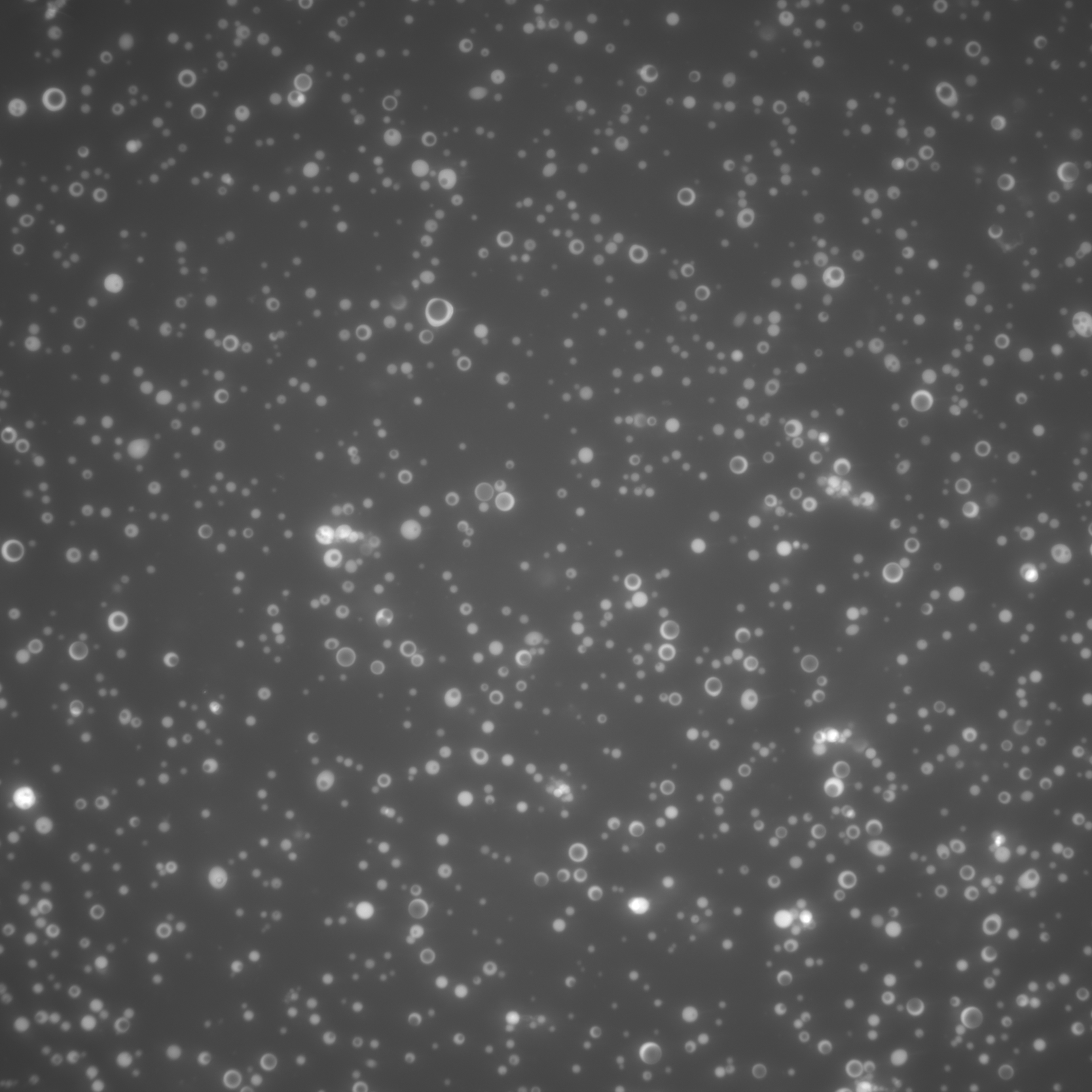

Supplement: Supplementary file 4 — Source data Fig. 2 [file 44318_2025_431_MOESM4_ESM.zip › Figure 2 copy/2A/polyU_RNA/InputData/25HD/2,5-HD 3.tif]

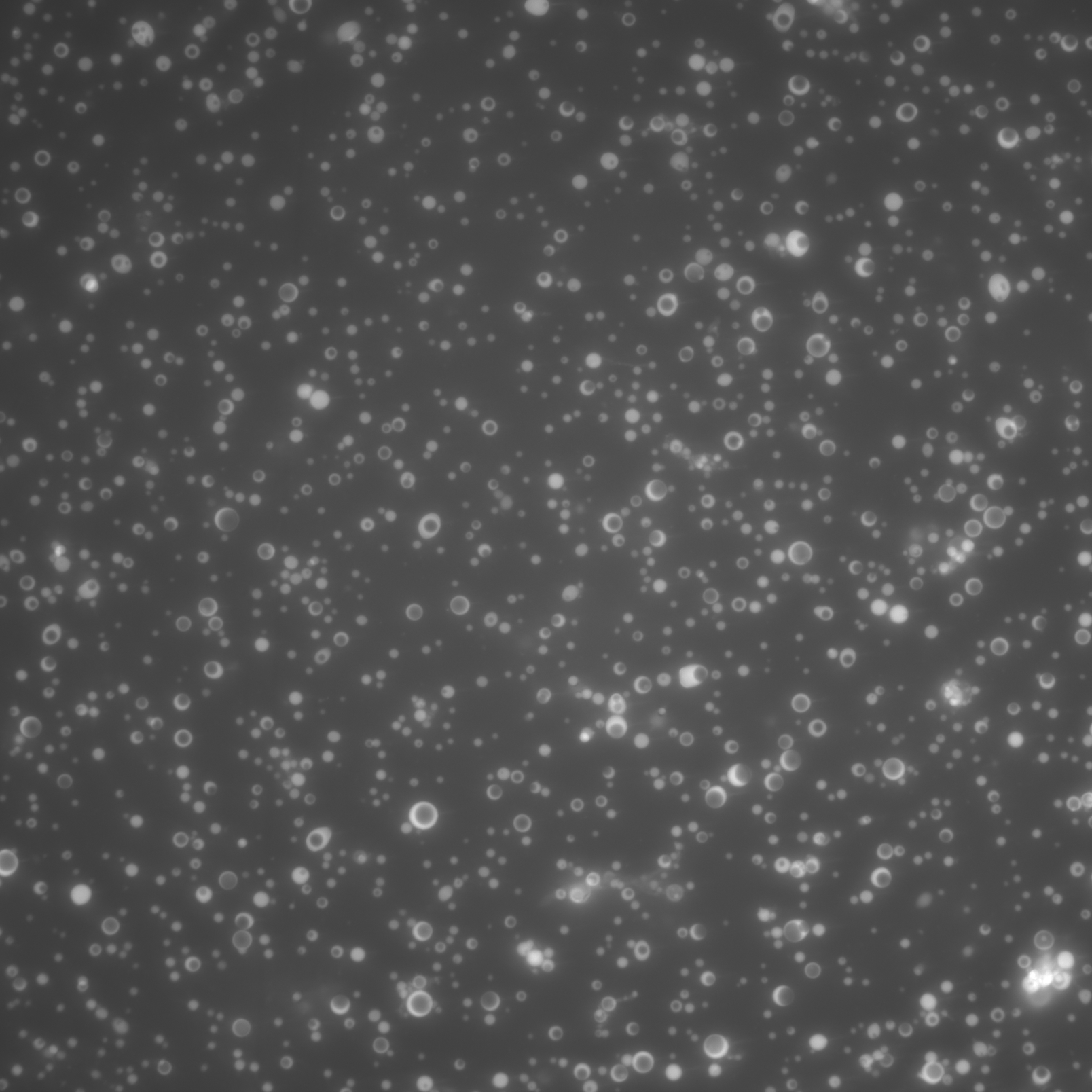

Supplement: Supplementary file 4 — Source data Fig. 2 [file 44318_2025_431_MOESM4_ESM.zip › Figure 2 copy/2A/polyU_RNA/InputData/25HD/2,5-HD 2.tif]

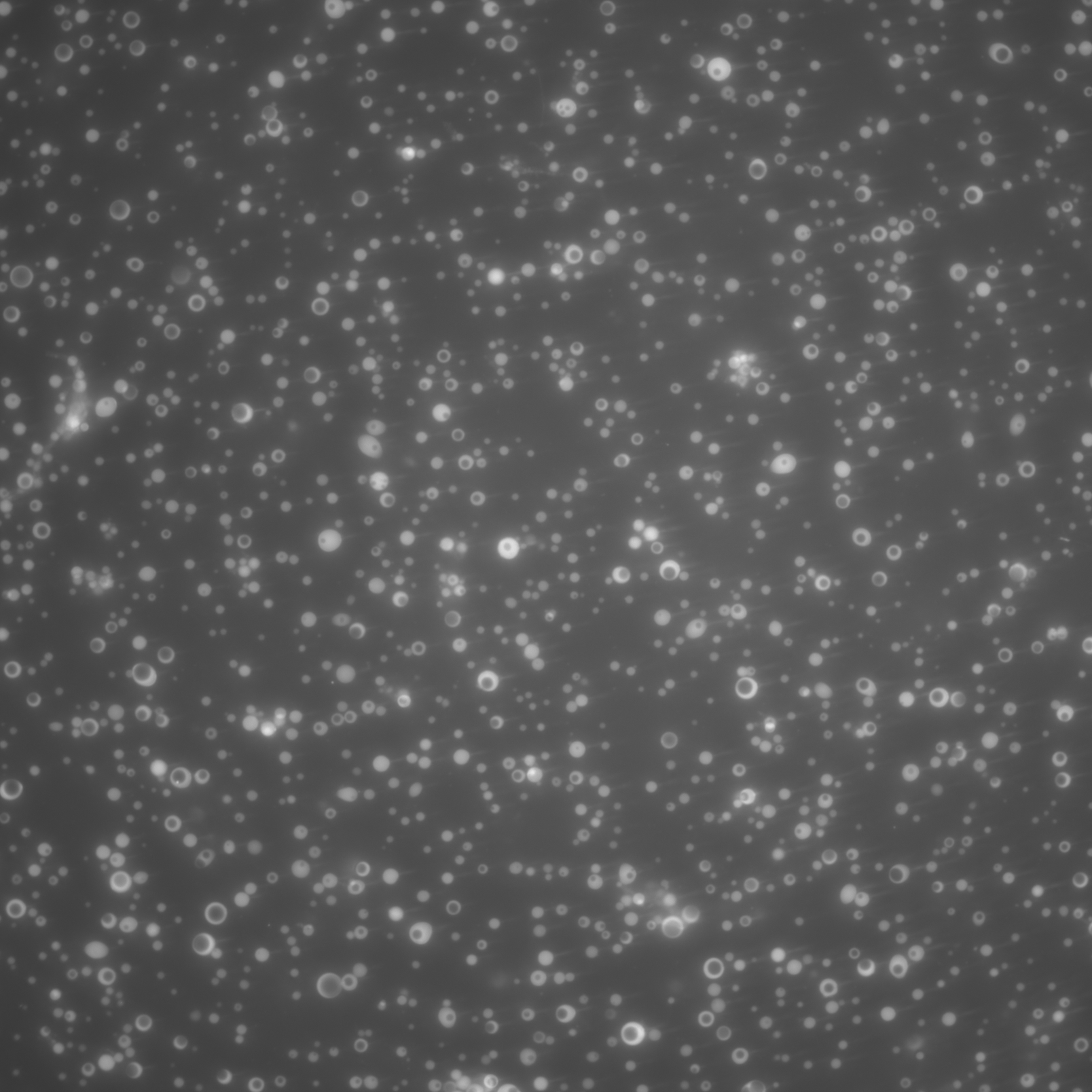

Supplement: Supplementary file 4 — Source data Fig. 2 [file 44318_2025_431_MOESM4_ESM.zip › Figure 2 copy/2A/polyU_RNA/InputData/25HD/2,5-HD 1.tif]

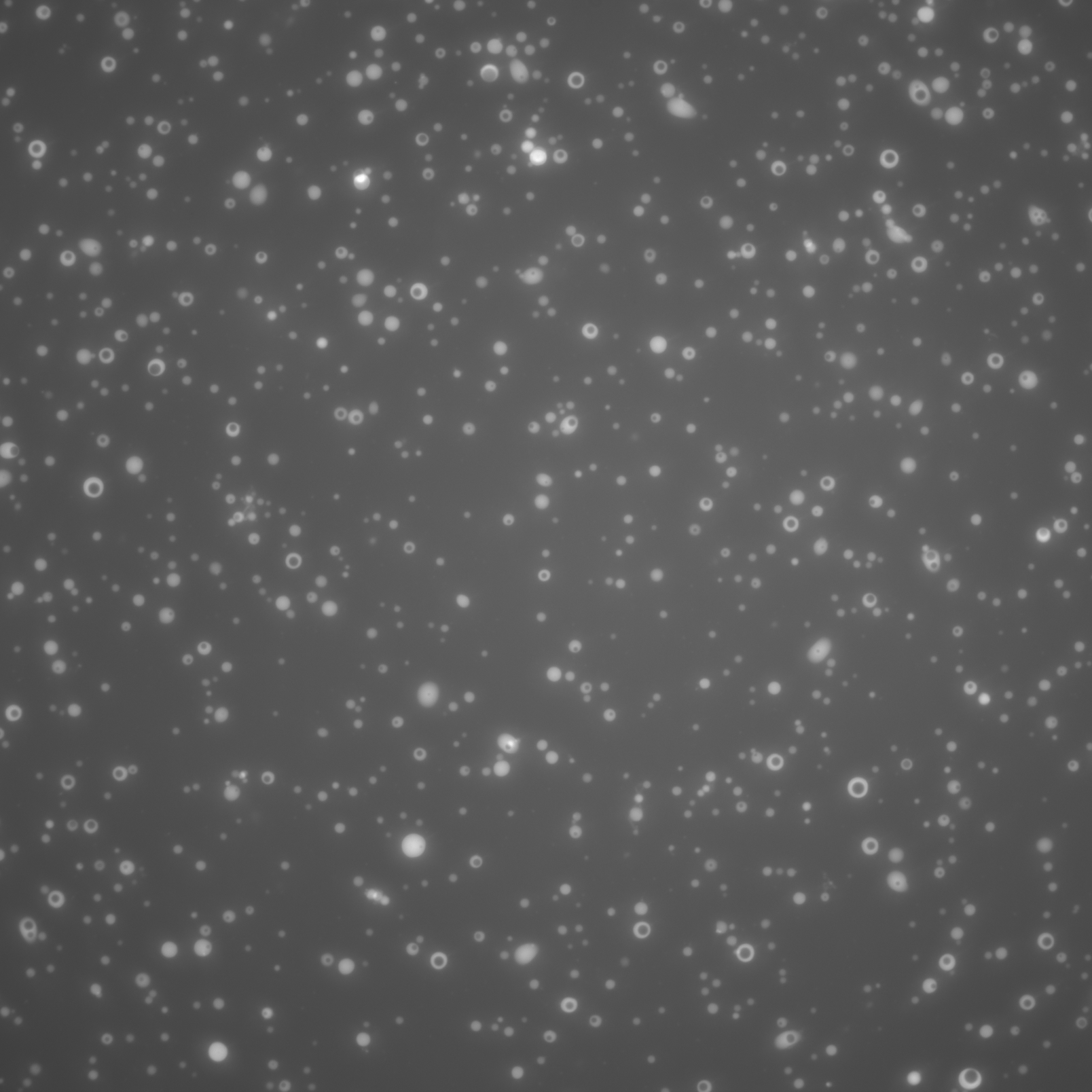

Supplement: Supplementary file 4 — Source data Fig. 2 [file 44318_2025_431_MOESM4_ESM.zip › Figure 2 copy/2A/polyU_RNA/InputData/14BD/1,4-BD 1.tif]

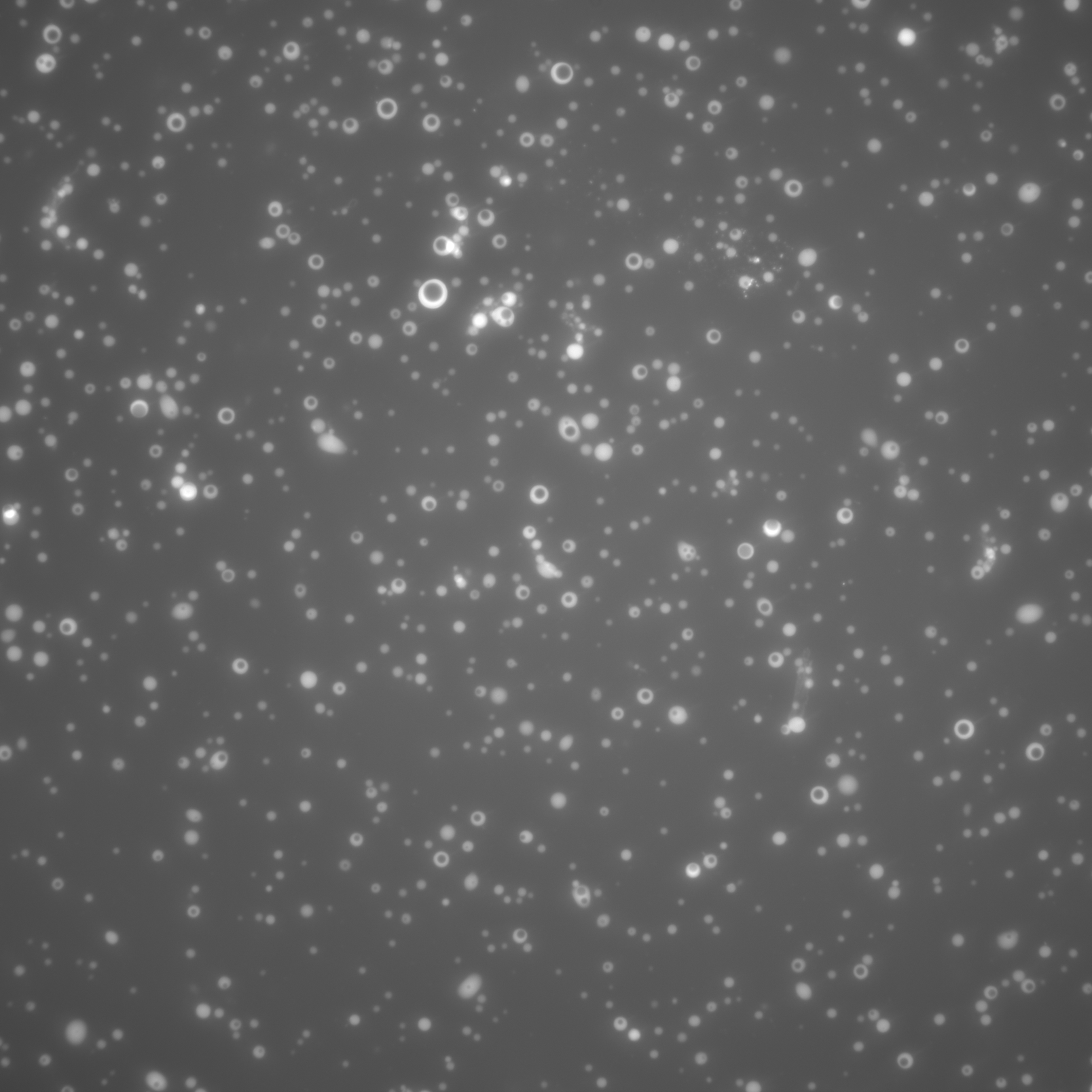

Supplement: Supplementary file 4 — Source data Fig. 2 [file 44318_2025_431_MOESM4_ESM.zip › Figure 2 copy/2A/polyU_RNA/InputData/14BD/1,4-BD 3.tif]

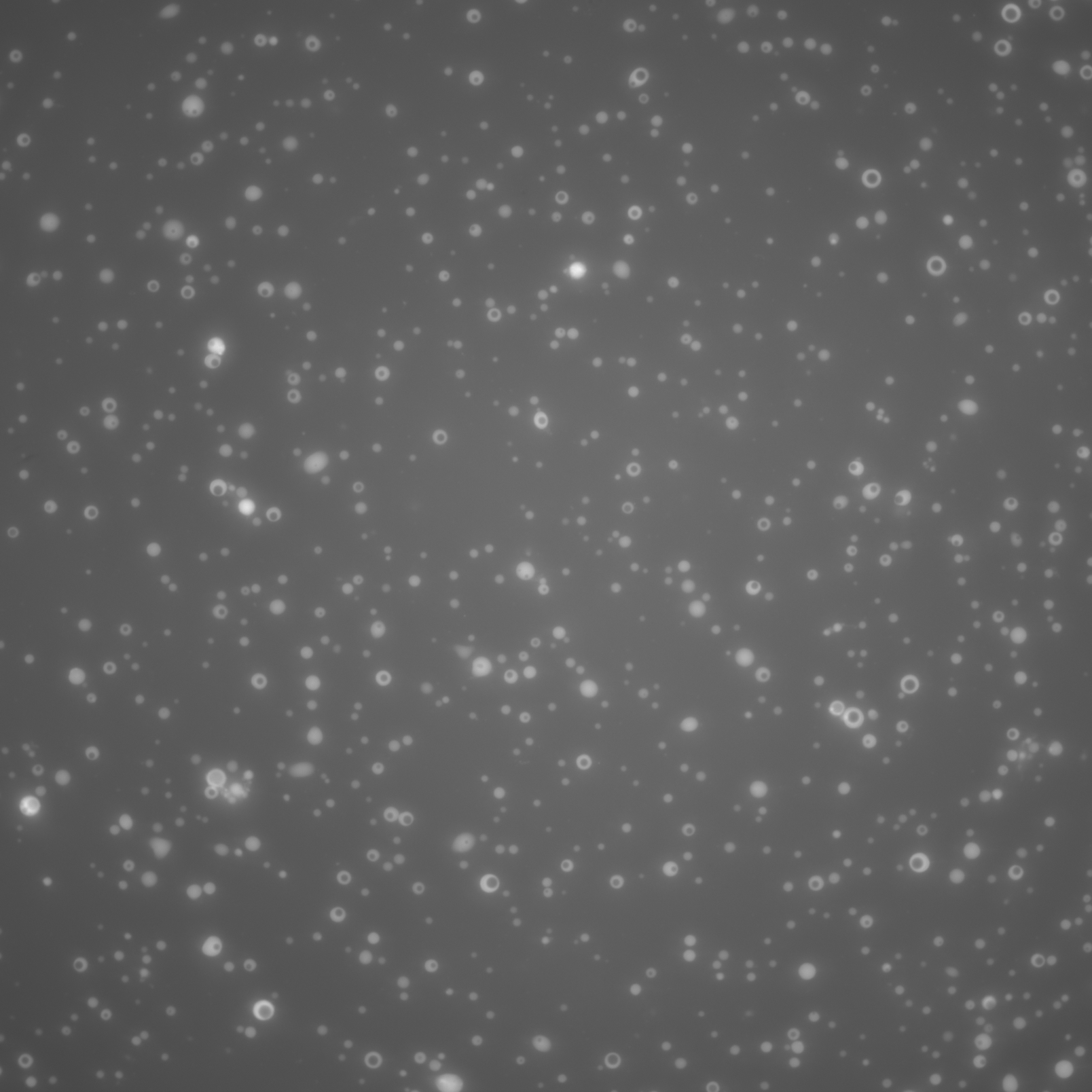

Supplement: Supplementary file 4 — Source data Fig. 2 [file 44318_2025_431_MOESM4_ESM.zip › Figure 2 copy/2A/polyU_RNA/InputData/14BD/1,4-BD 2.tif]

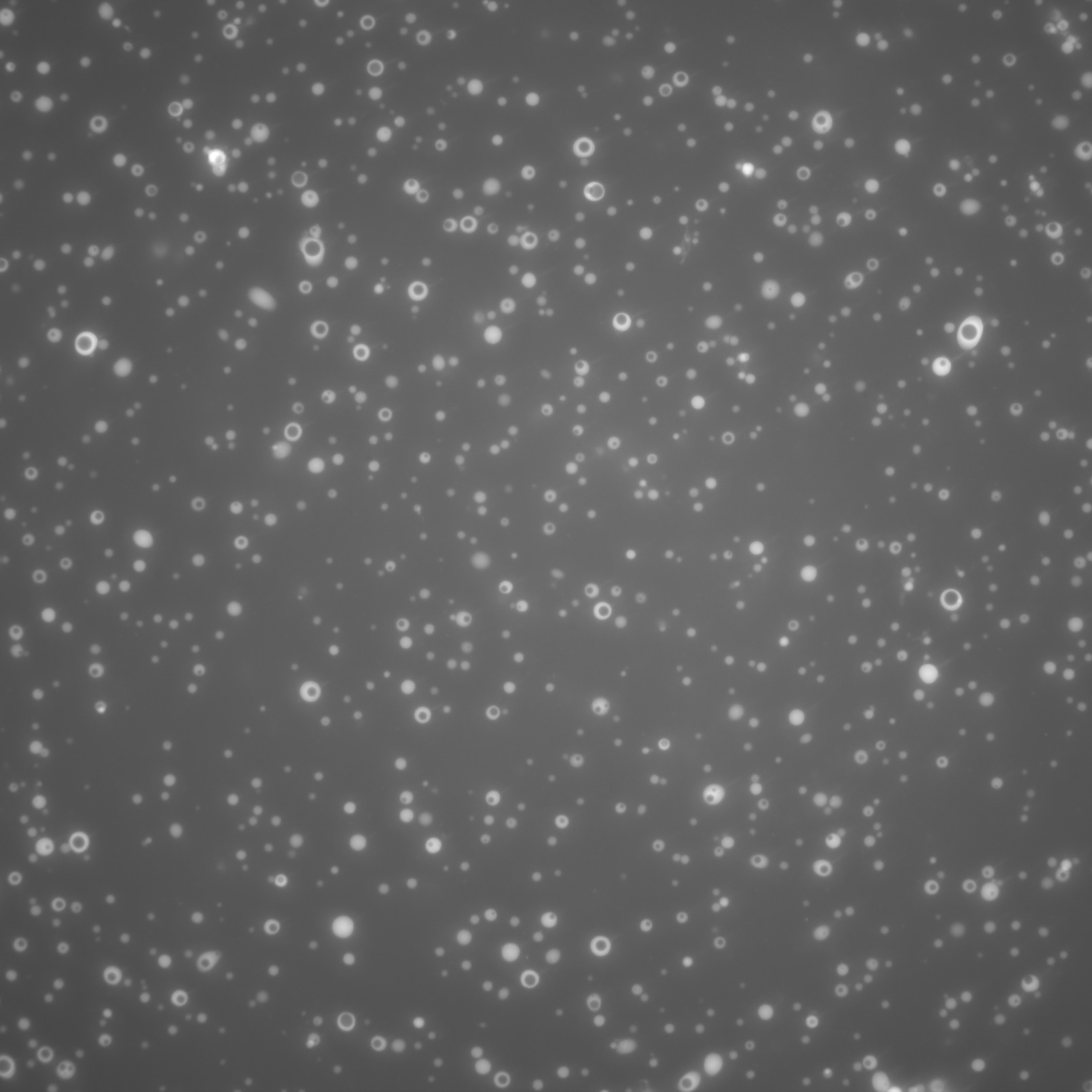

Supplement: Supplementary file 4 — Source data Fig. 2 [file 44318_2025_431_MOESM4_ESM.zip › Figure 2 copy/2A/polyU_RNA/InputData/14BD/1,4-BD 4.tif]

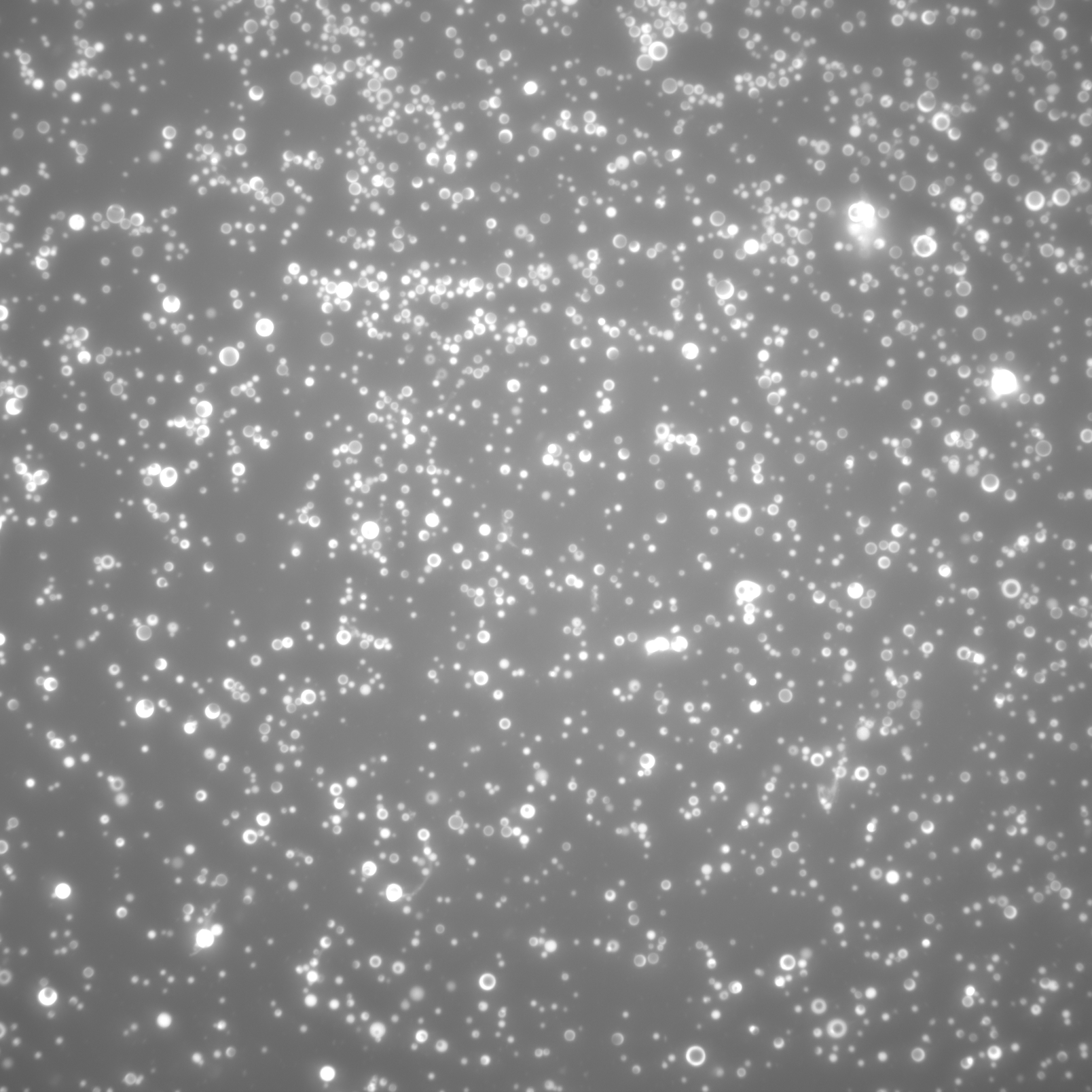

Supplement: Supplementary file 4 — Source data Fig. 2 [file 44318_2025_431_MOESM4_ESM.zip › Figure 2 copy/2A/polyU_RNA/InputData/NoDiol/no additivie 2.tif]

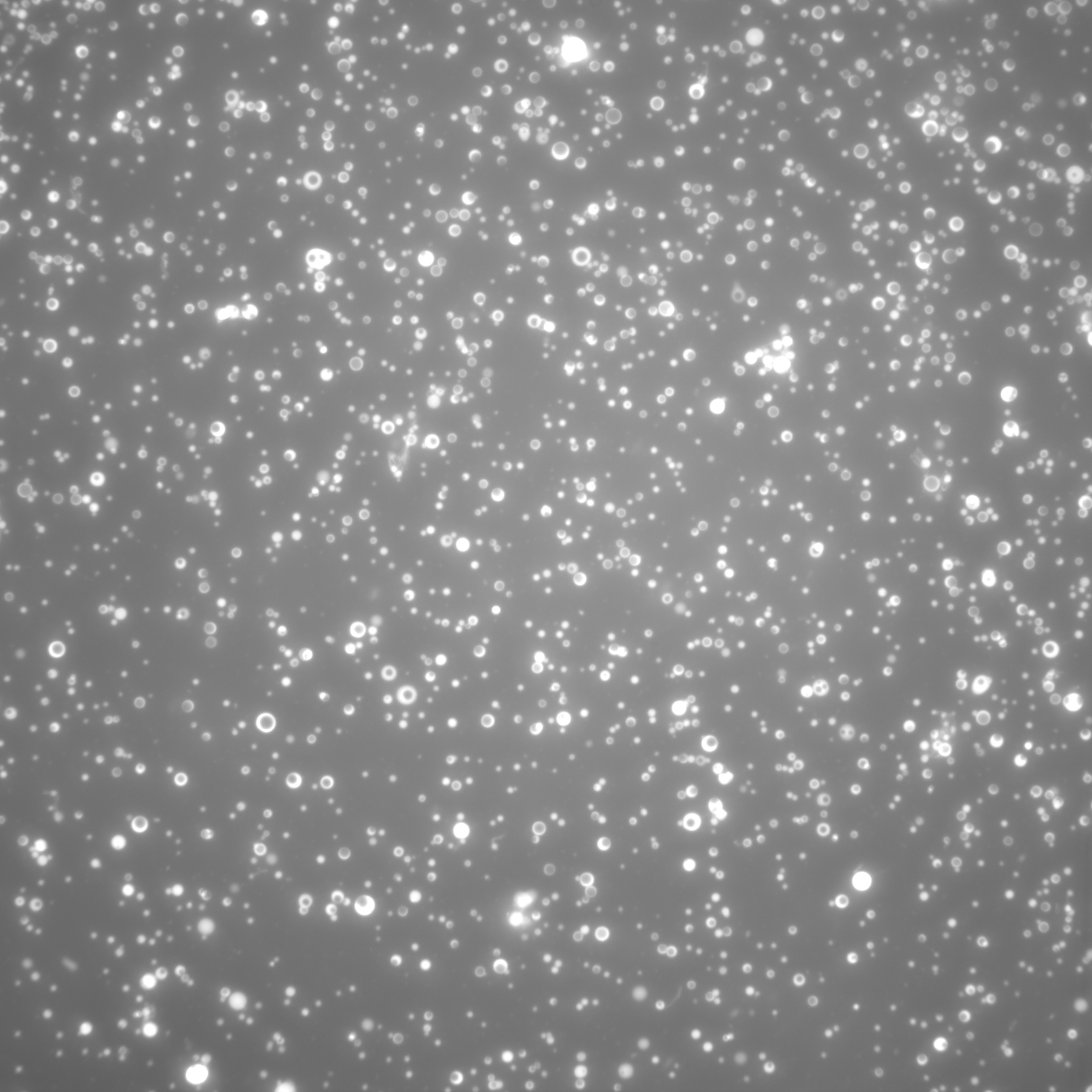

Supplement: Supplementary file 4 — Source data Fig. 2 [file 44318_2025_431_MOESM4_ESM.zip › Figure 2 copy/2A/polyU_RNA/InputData/NoDiol/no additivie 3.tif]

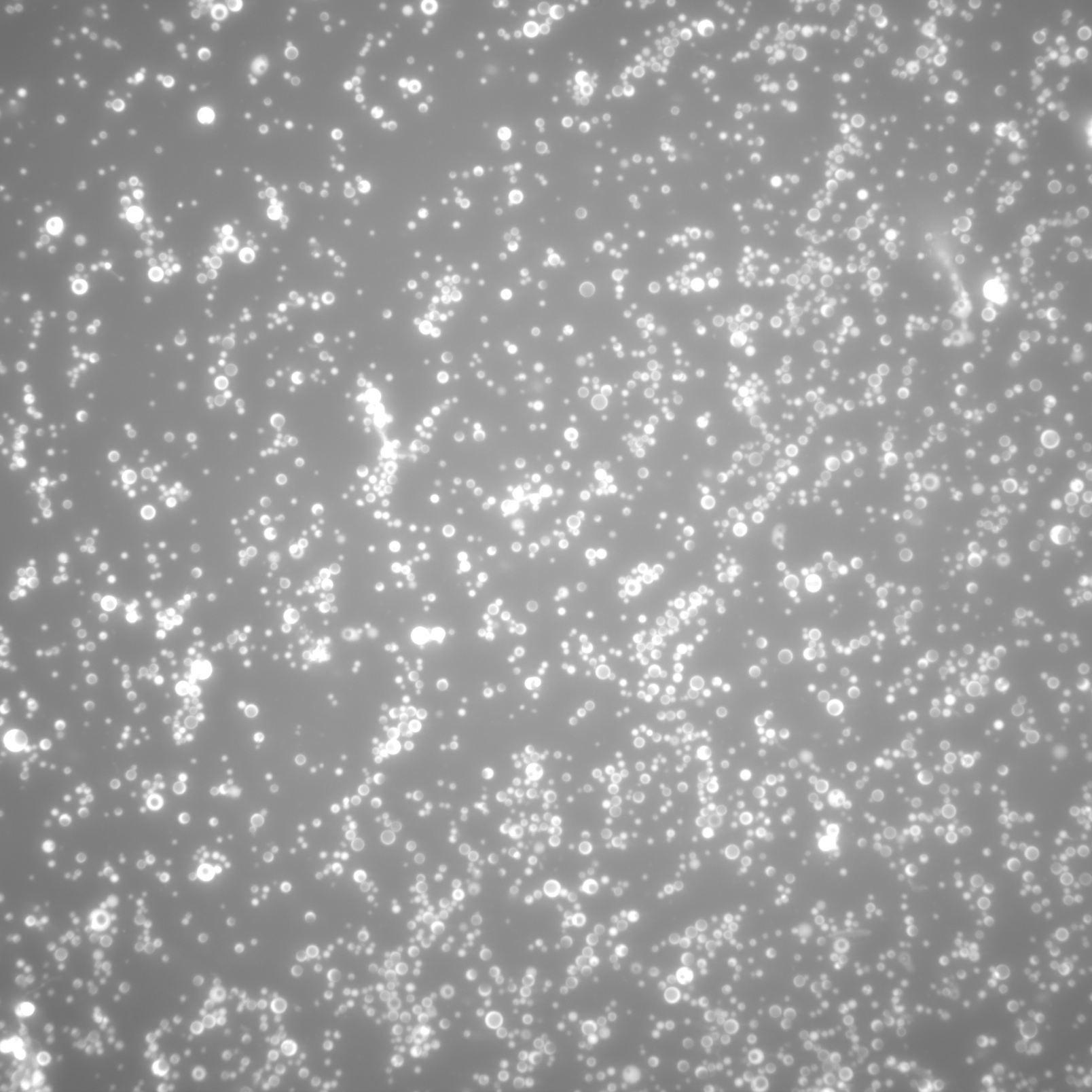

Supplement: Supplementary file 4 — Source data Fig. 2 [file 44318_2025_431_MOESM4_ESM.zip › Figure 2 copy/2A/polyU_RNA/InputData/NoDiol/no additivie 1.tif]

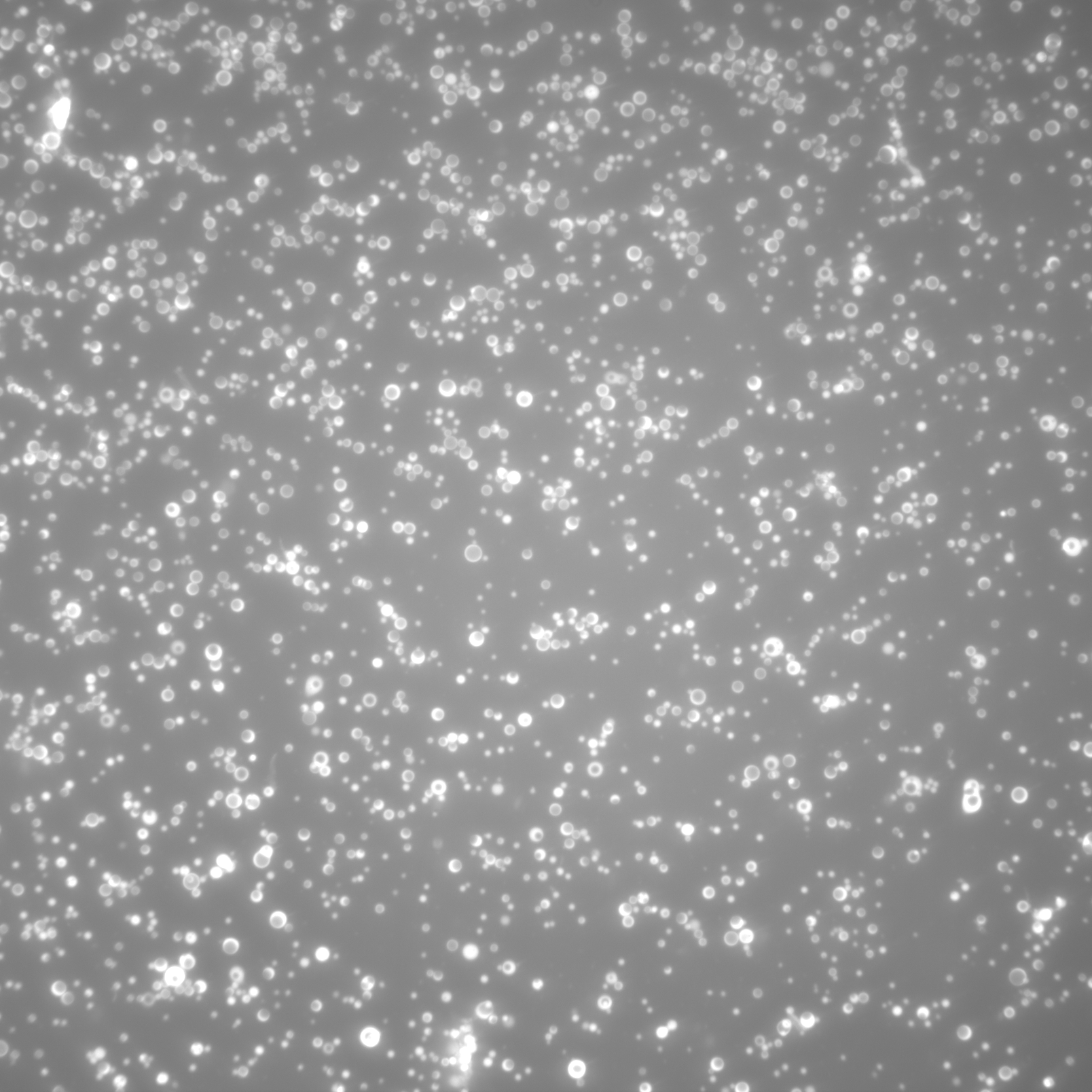

Supplement: Supplementary file 4 — Source data Fig. 2 [file 44318_2025_431_MOESM4_ESM.zip › Figure 2 copy/2A/polyU_RNA/InputData/NoDiol/no additivie 4.tif]

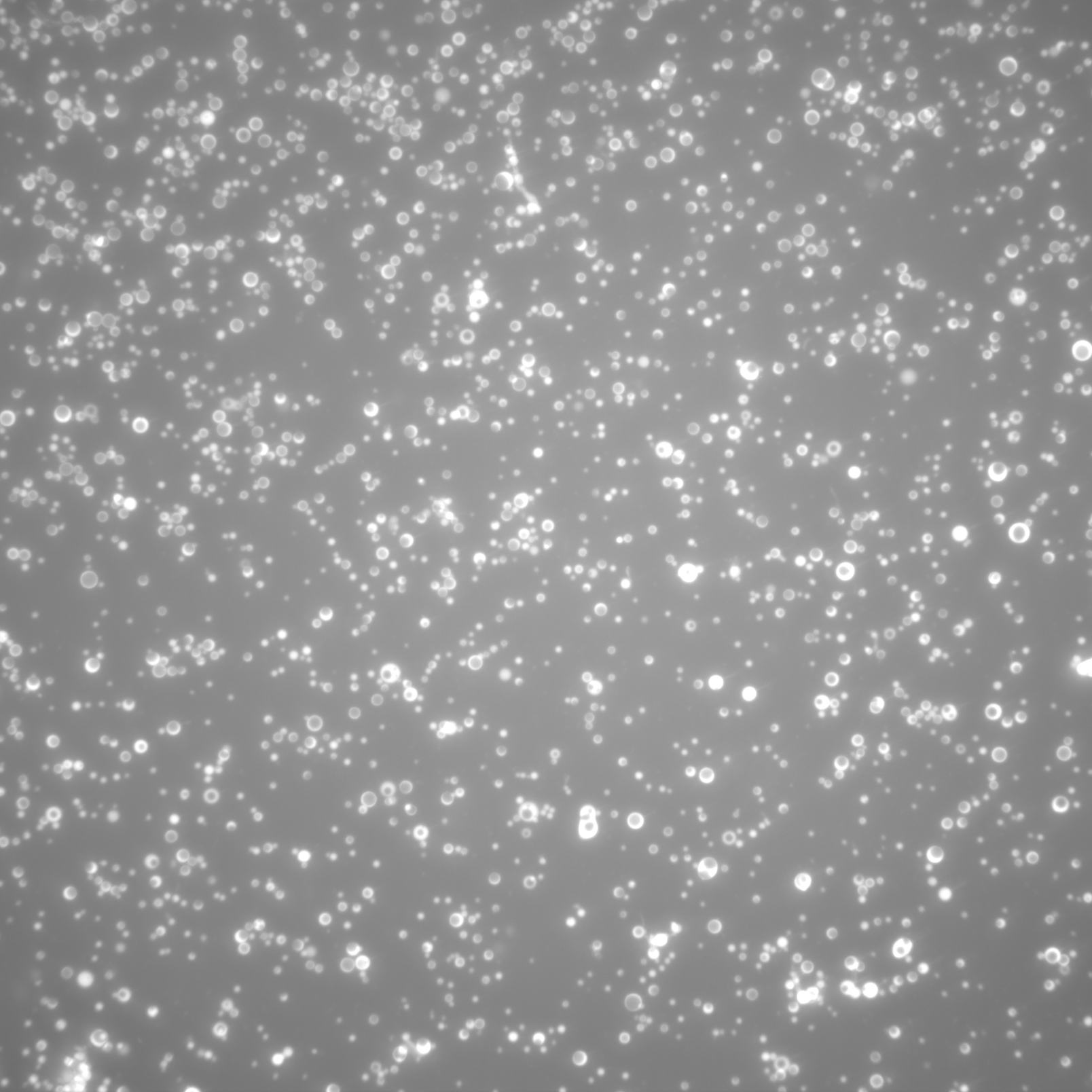

Supplement: Supplementary file 4 — Source data Fig. 2 [file 44318_2025_431_MOESM4_ESM.zip › Figure 2 copy/2A/polyU_RNA/InputData/NoDiol/no additivie 5.tif]

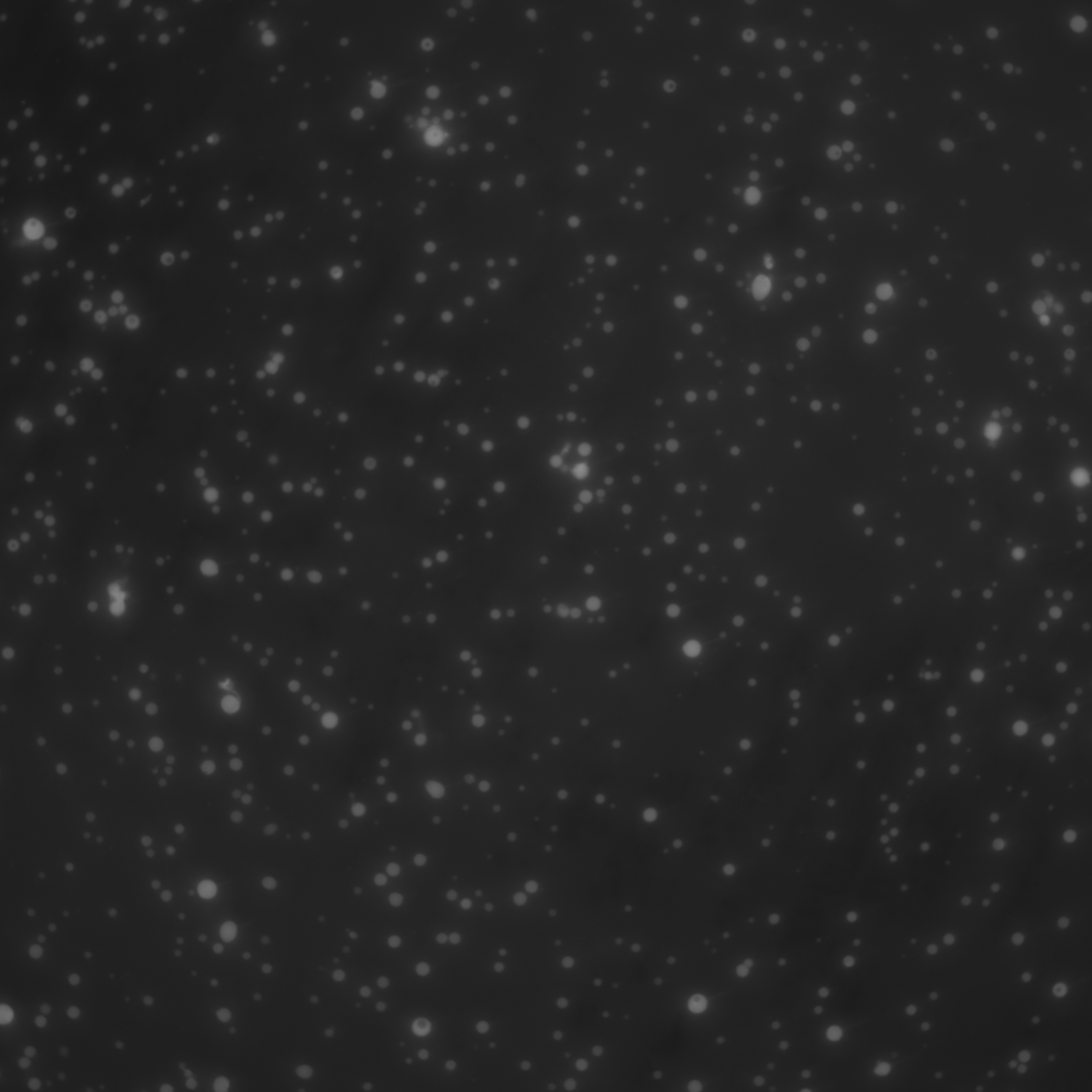

Supplement: Supplementary file 4 — Source data Fig. 2 [file 44318_2025_431_MOESM4_ESM.zip › Figure 2 copy/2A/polyU_RNA/InputData/12HD/1,2-HD 2.tif]

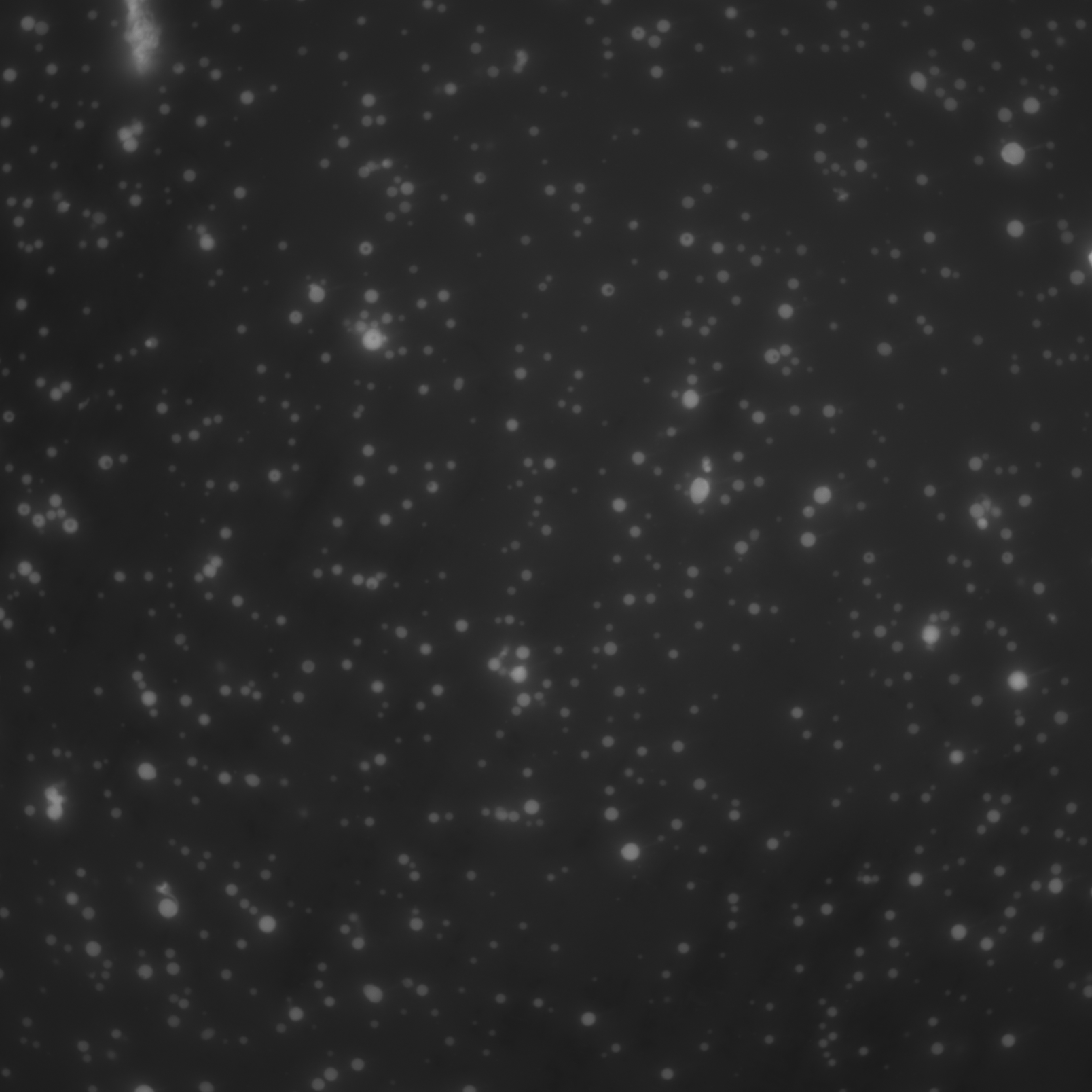

Supplement: Supplementary file 4 — Source data Fig. 2 [file 44318_2025_431_MOESM4_ESM.zip › Figure 2 copy/2A/polyU_RNA/InputData/12HD/1,2-HD 3.tif]

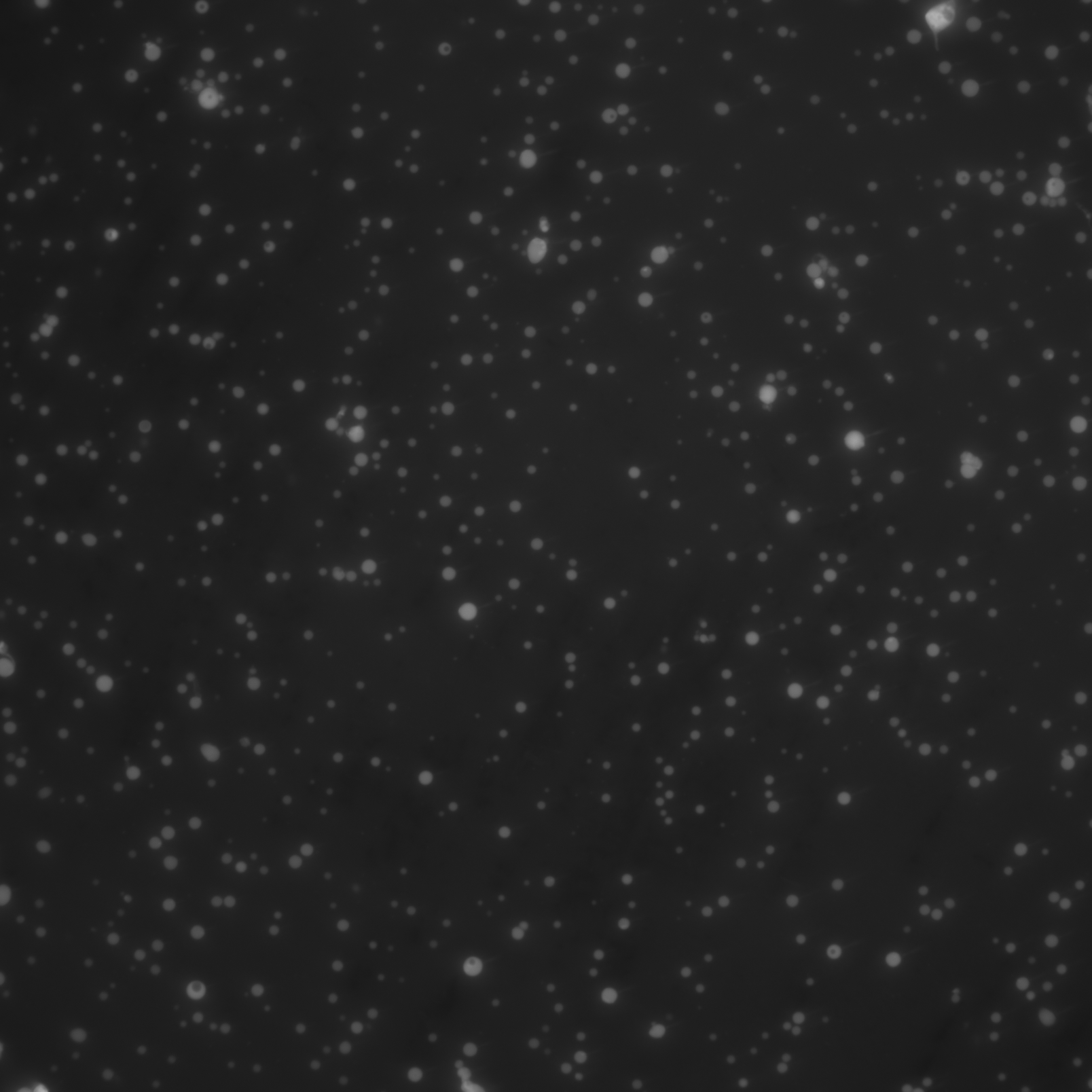

Supplement: Supplementary file 4 — Source data Fig. 2 [file 44318_2025_431_MOESM4_ESM.zip › Figure 2 copy/2A/polyU_RNA/InputData/12HD/1,2-HD 1.tif]

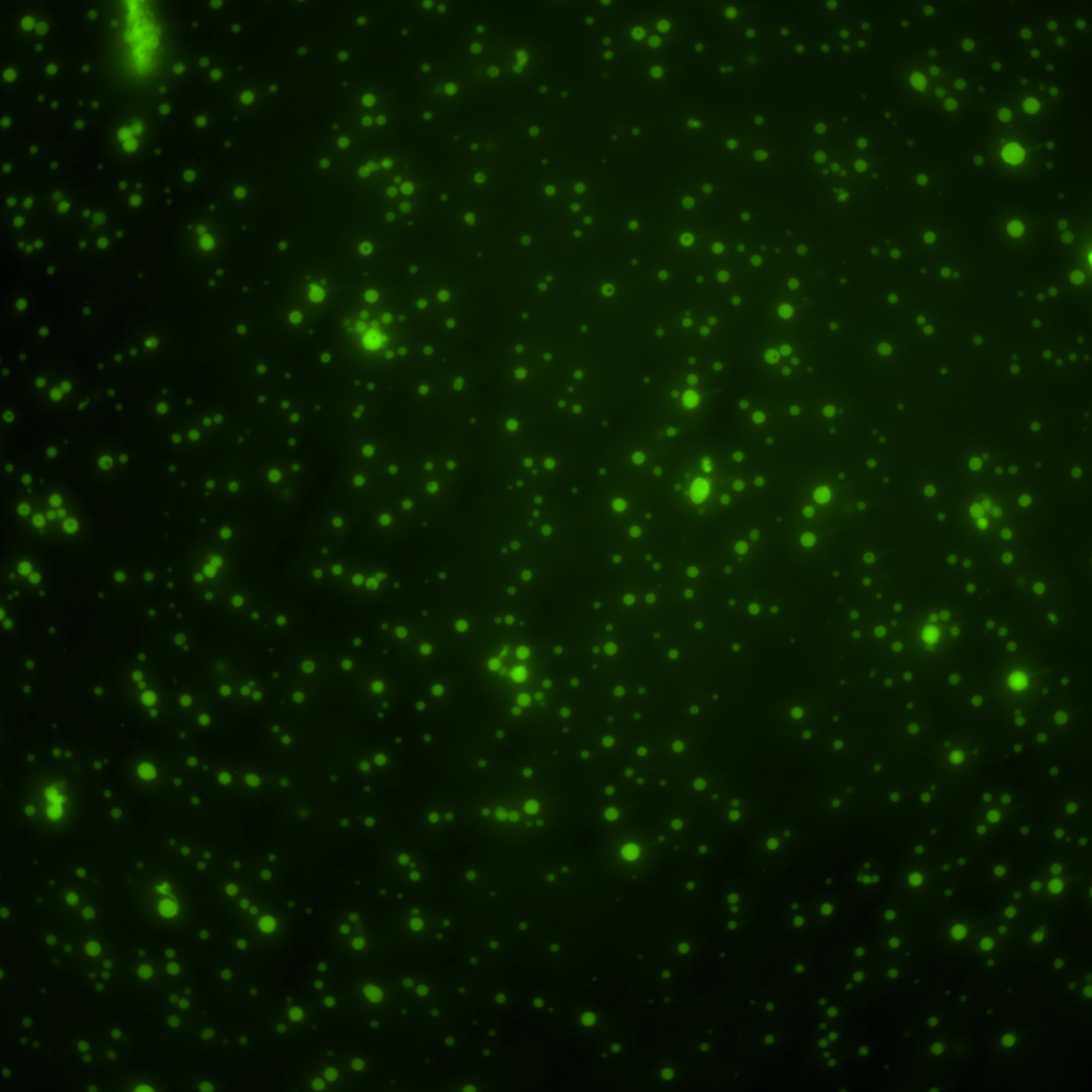

Supplement: Supplementary file 4 — Source data Fig. 2 [file 44318_2025_431_MOESM4_ESM.zip › Figure 2 copy/2A/polyU_RNA/InputData/12HD/1,2-HD 3.png]

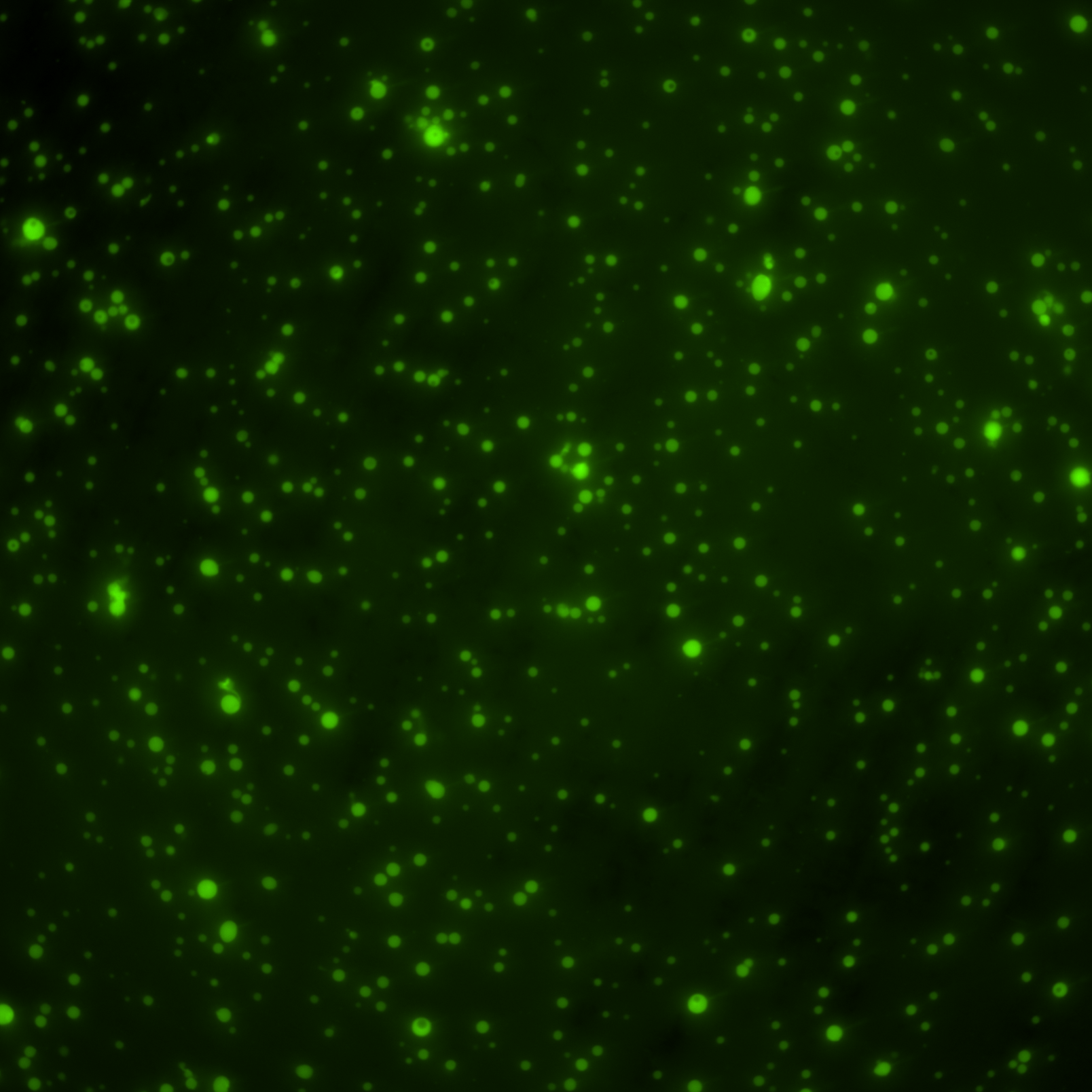

Supplement: Supplementary file 4 — Source data Fig. 2 [file 44318_2025_431_MOESM4_ESM.zip › Figure 2 copy/2A/polyU_RNA/InputData/12HD/1,2-HD 2.png]

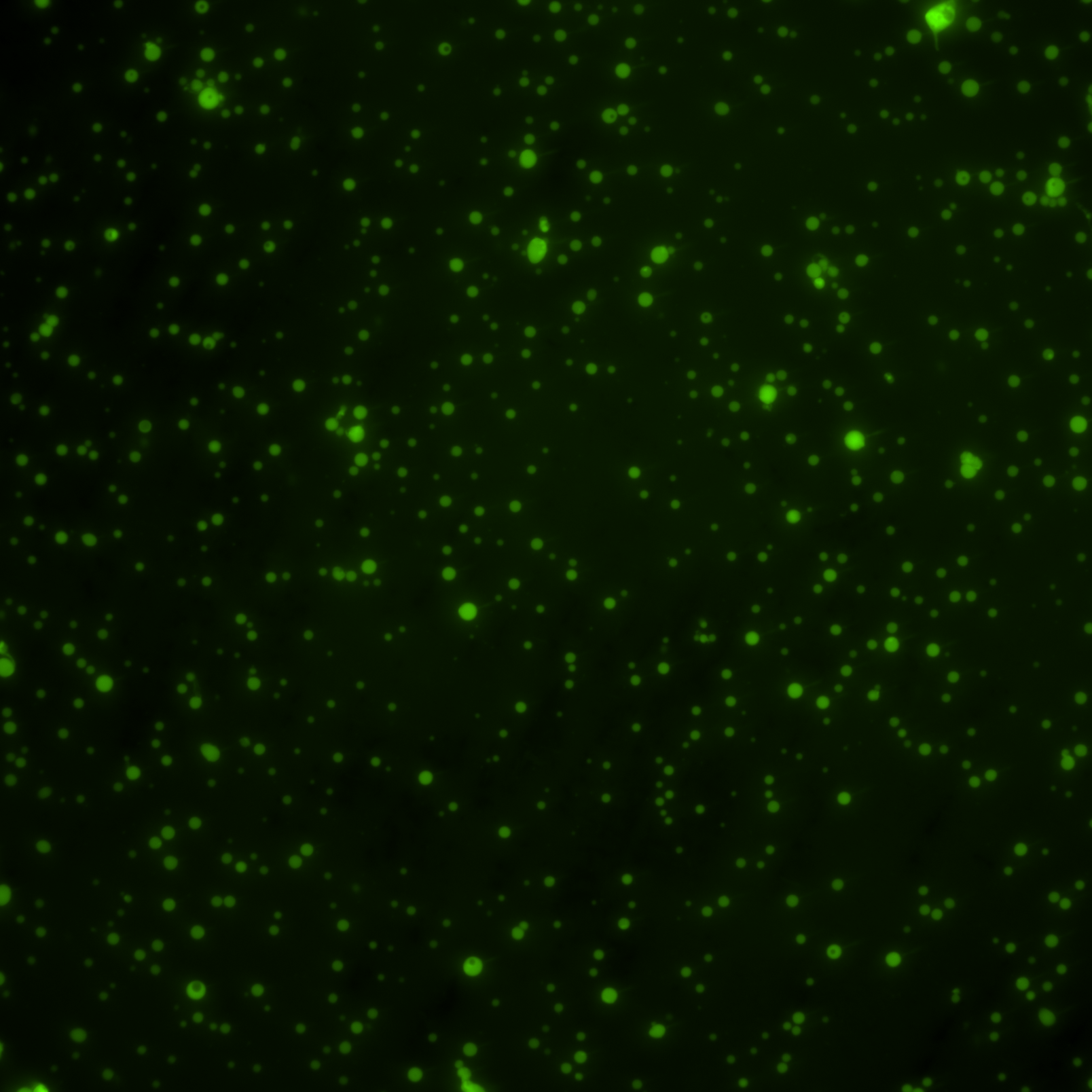

Supplement: Supplementary file 4 — Source data Fig. 2 [file 44318_2025_431_MOESM4_ESM.zip › Figure 2 copy/2A/polyU_RNA/InputData/12HD/1,2-HD 1.png]

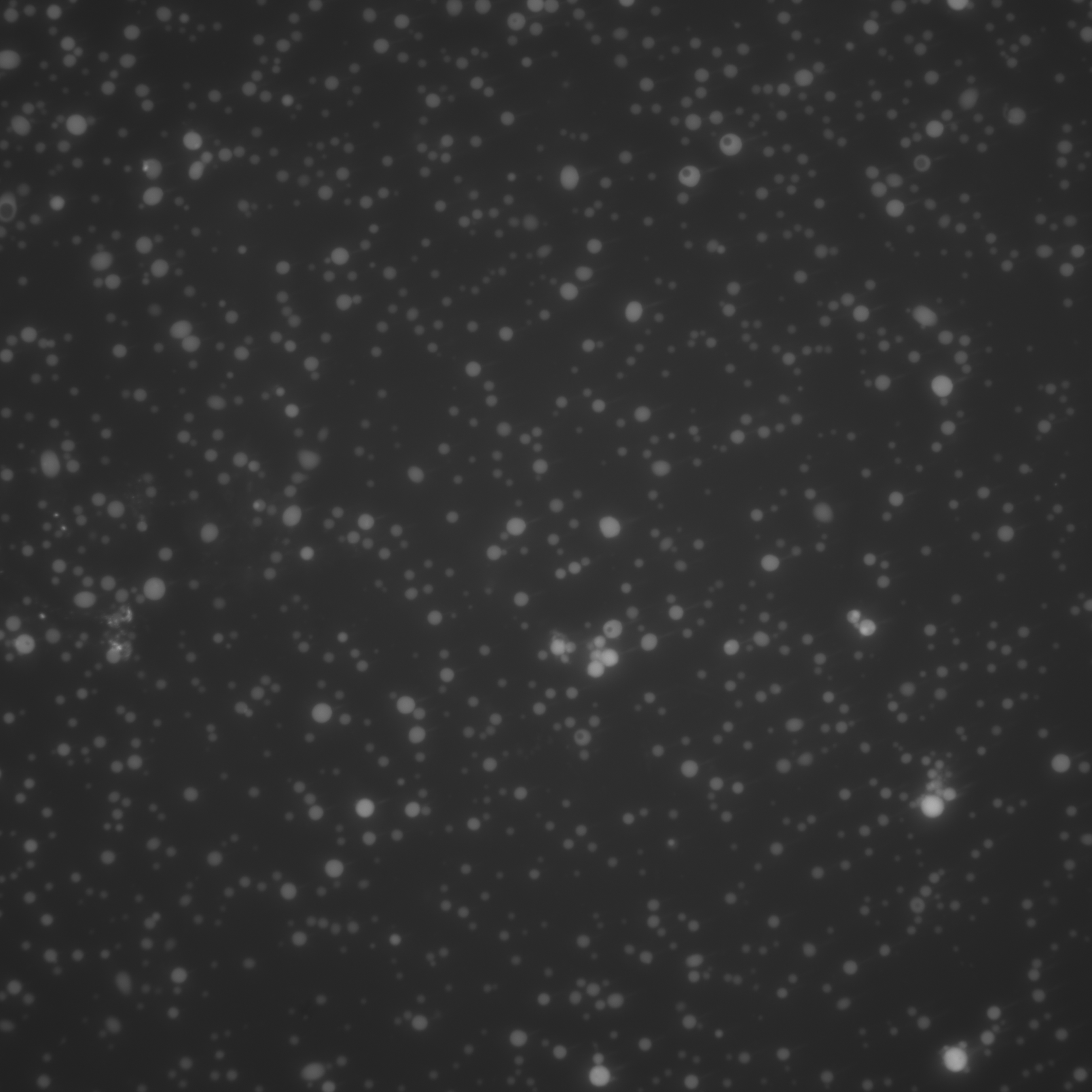

Supplement: Supplementary file 4 — Source data Fig. 2 [file 44318_2025_431_MOESM4_ESM.zip › Figure 2 copy/2A/polyU_RNA/InputData/16HD/1,6-HD 4.tif]

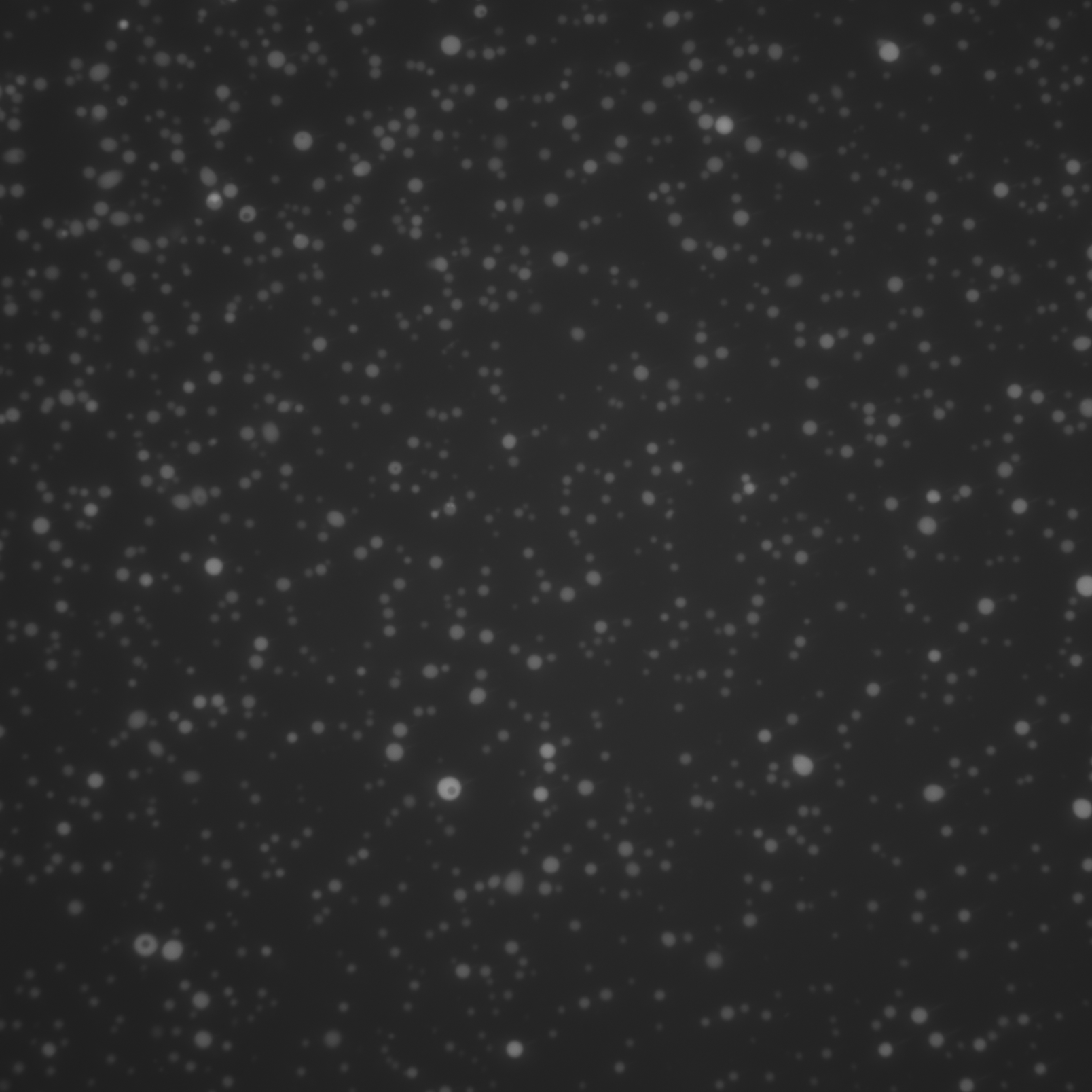

Supplement: Supplementary file 4 — Source data Fig. 2 [file 44318_2025_431_MOESM4_ESM.zip › Figure 2 copy/2A/polyU_RNA/InputData/16HD/1,6-HD 1.tif]

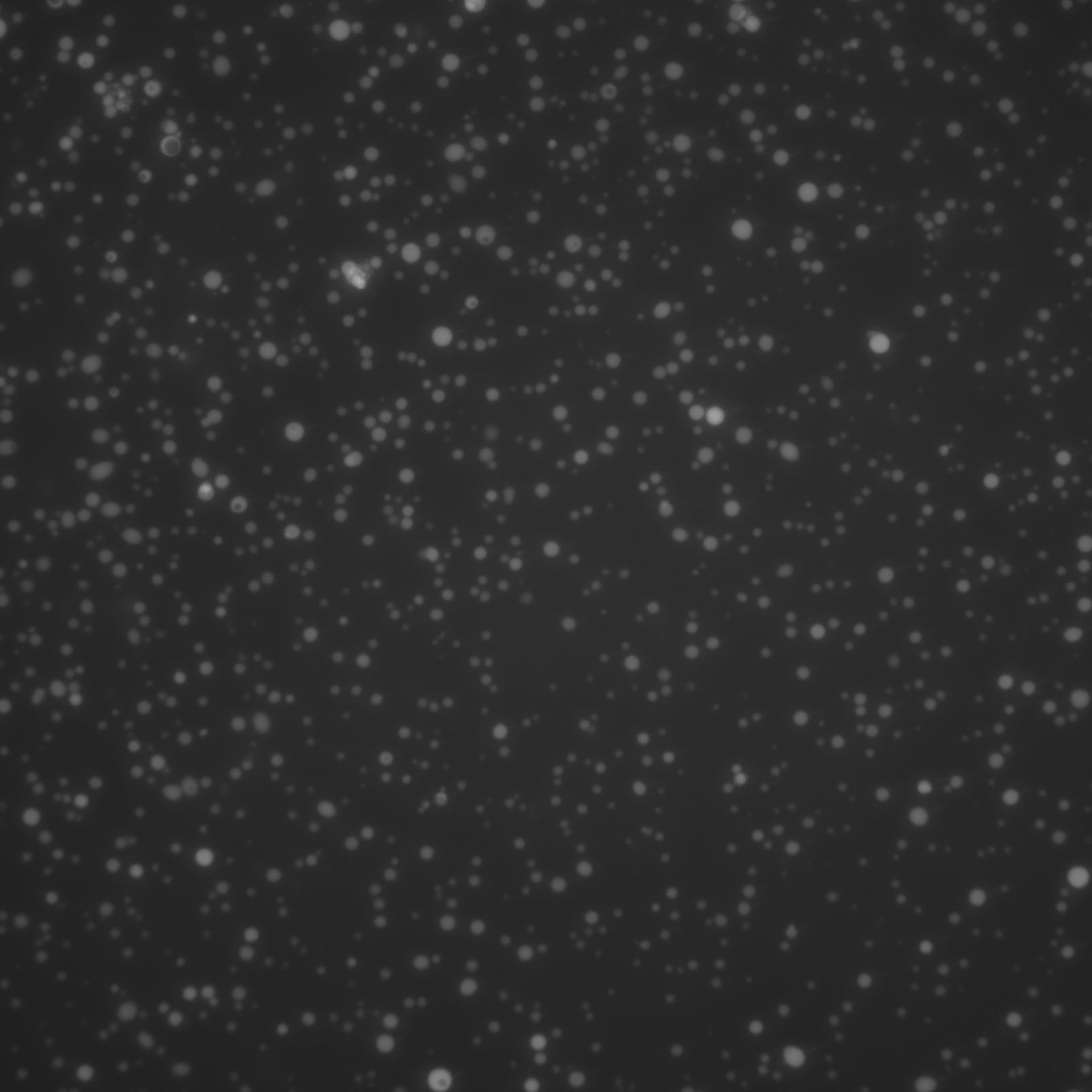

Supplement: Supplementary file 4 — Source data Fig. 2 [file 44318_2025_431_MOESM4_ESM.zip › Figure 2 copy/2A/polyU_RNA/InputData/16HD/1,6-HD 2.tif]

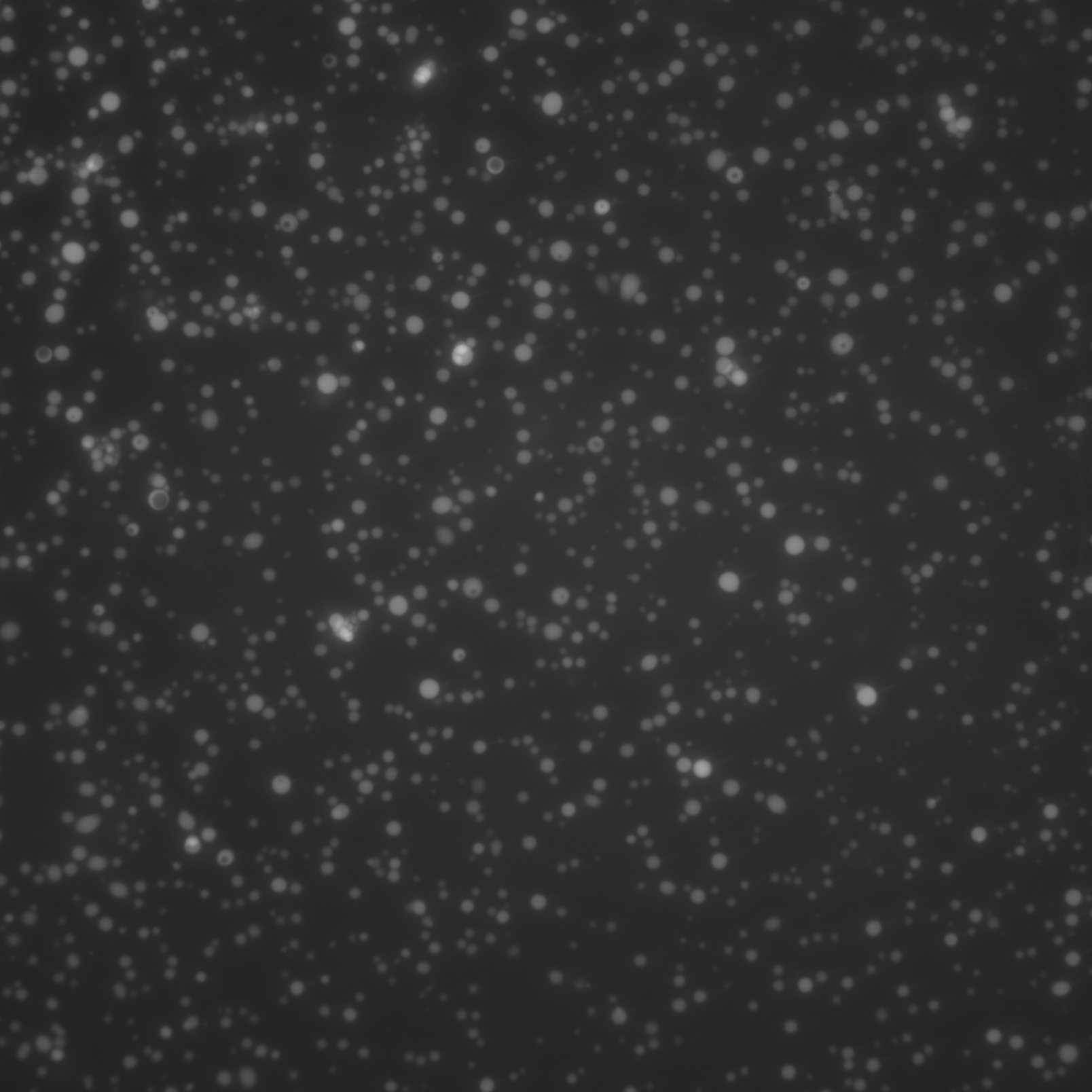

Supplement: Supplementary file 4 — Source data Fig. 2 [file 44318_2025_431_MOESM4_ESM.zip › Figure 2 copy/2A/polyU_RNA/InputData/16HD/1,6-HD 3.tif]

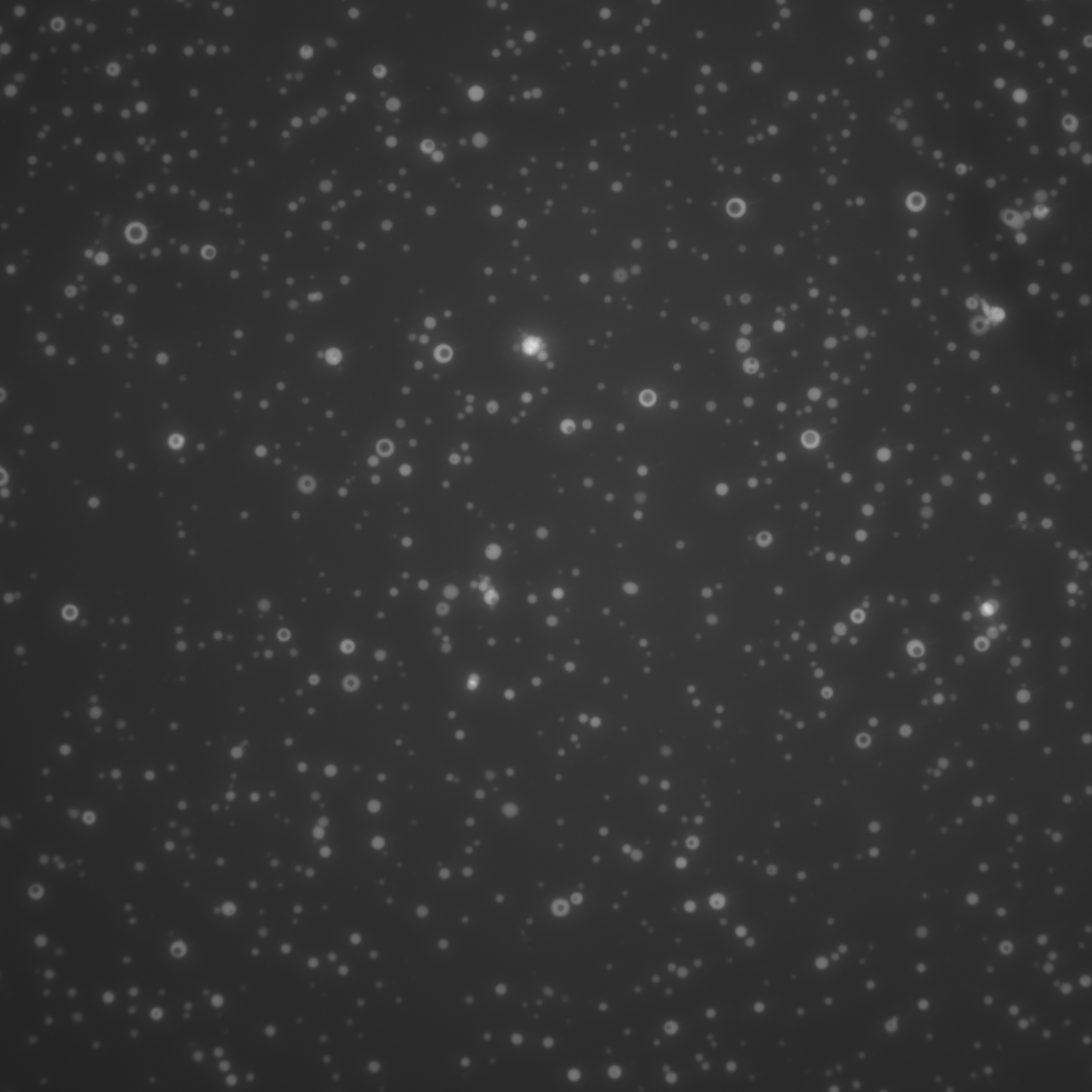

Supplement: Supplementary file 4 — Source data Fig. 2 [file 44318_2025_431_MOESM4_ESM.zip › Figure 2 copy/2A/polyU_RNA/InputData/15PD/1,5-PD 3.tif]

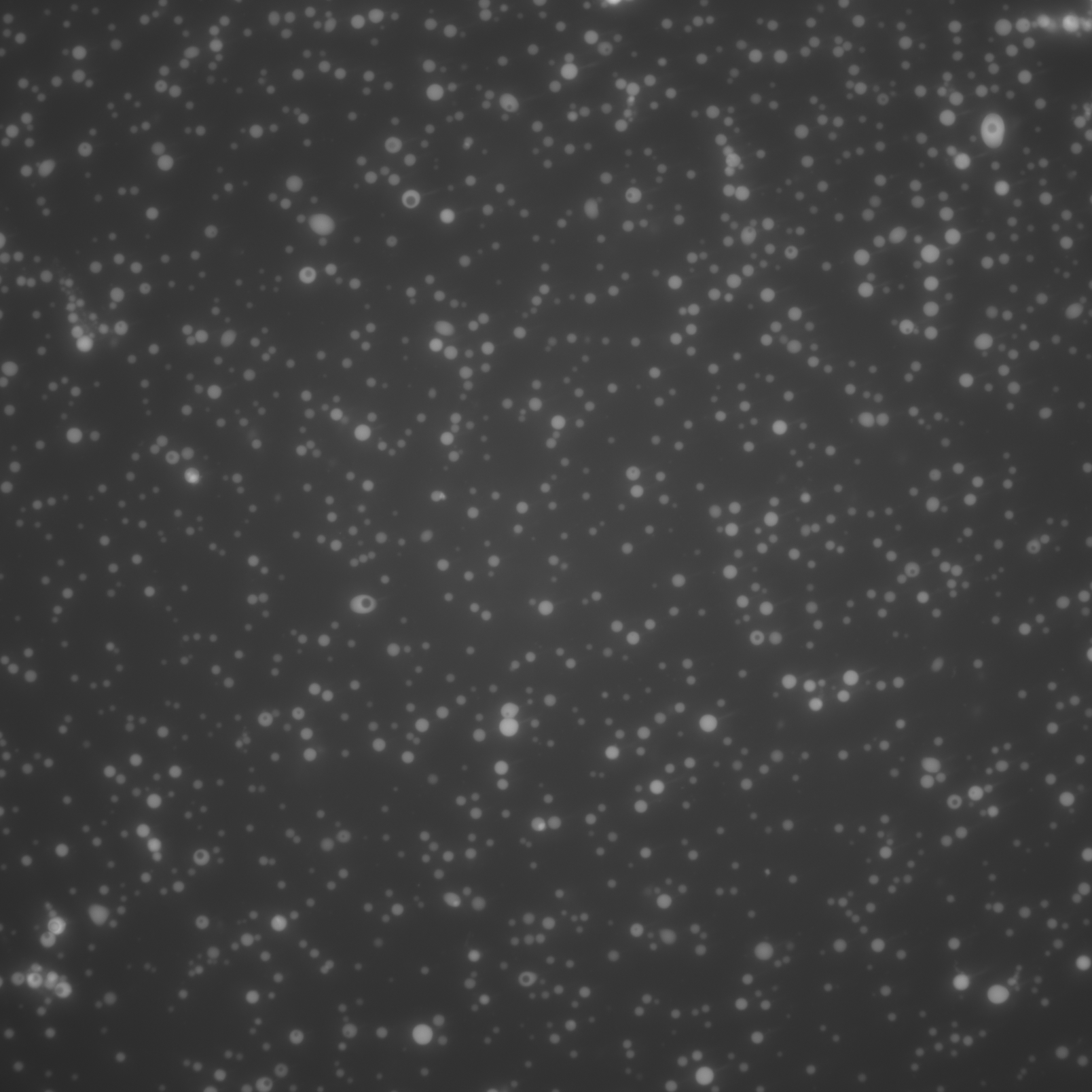

Supplement: Supplementary file 4 — Source data Fig. 2 [file 44318_2025_431_MOESM4_ESM.zip › Figure 2 copy/2A/polyU_RNA/InputData/15PD/1,5-PD 2.tif]

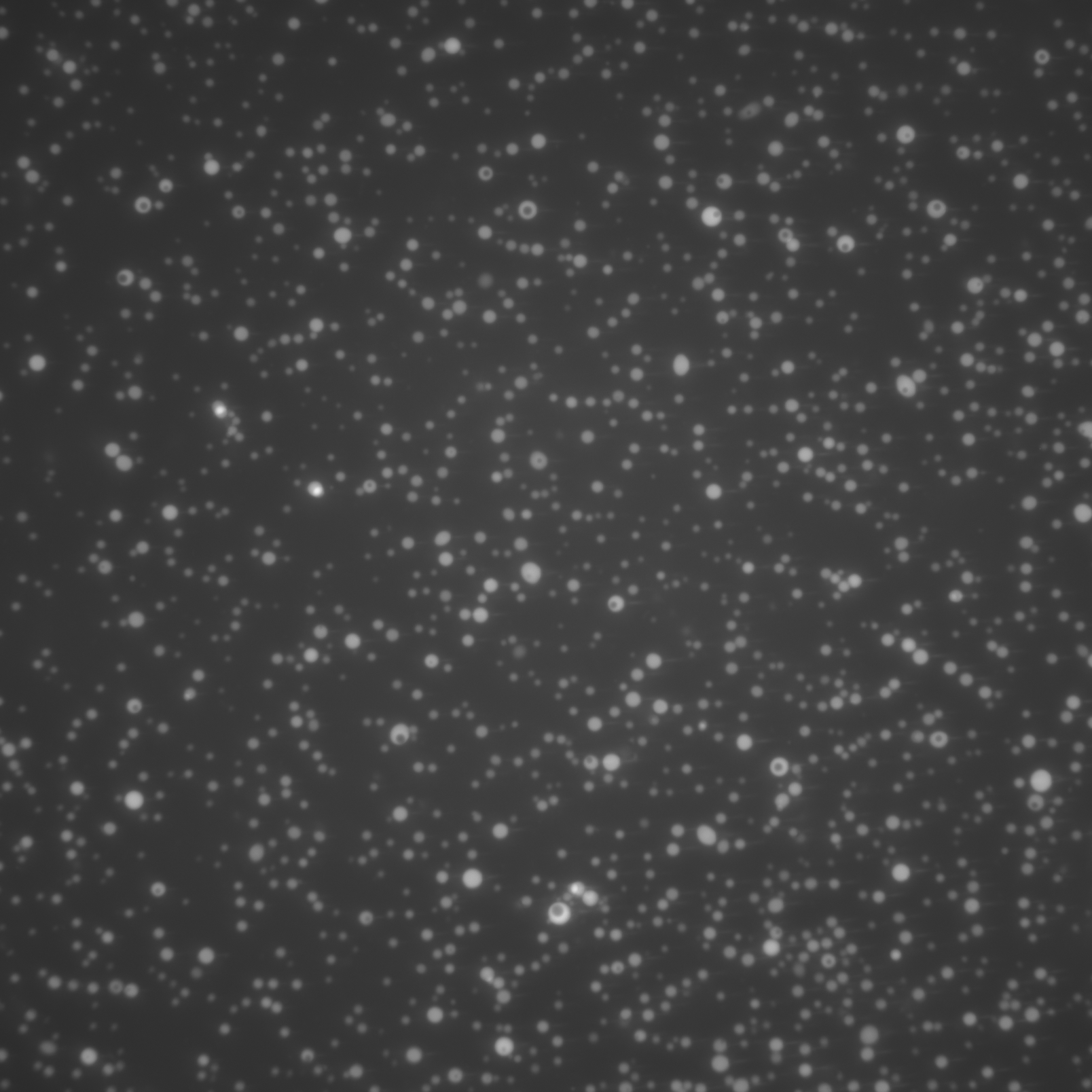

Supplement: Supplementary file 4 — Source data Fig. 2 [file 44318_2025_431_MOESM4_ESM.zip › Figure 2 copy/2A/polyU_RNA/InputData/15PD/1,5-PD 1.tif]

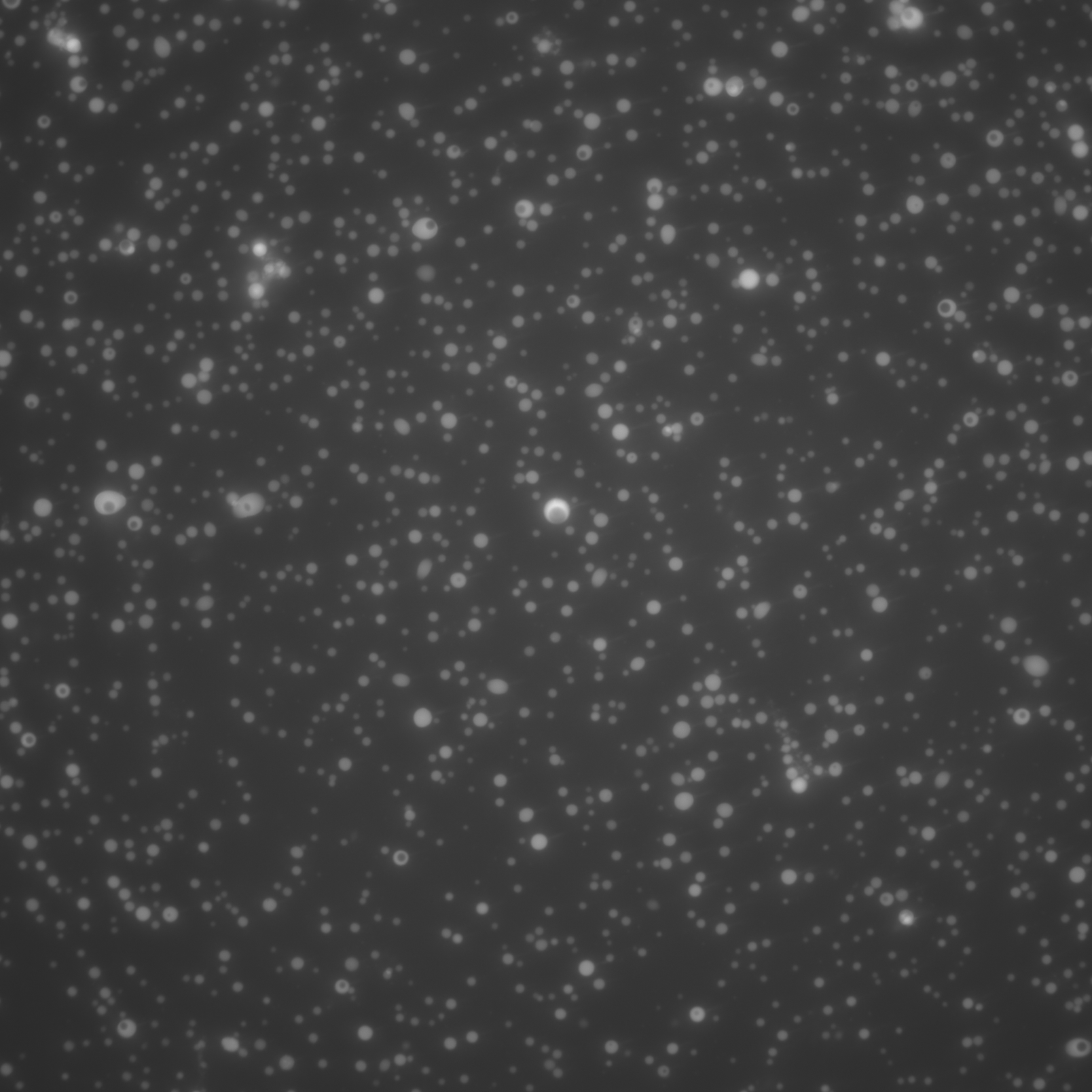

Supplement: Supplementary file 4 — Source data Fig. 2 [file 44318_2025_431_MOESM4_ESM.zip › Figure 2 copy/2A/polyU_RNA/InputData/15PD/1,5-PD 4.tif]
