# Supplementary material for: Molecular insights into the effect of 1,6-hexanediol on FUS phase separation
Source: EMBO J. 2025 Apr 25;44(10):2725–40. doi: 10.1038/s44318-025-00431-2 (PMC12084347; doi:10.1038/s44318-025-00431-2)
Supplement: Supplementary file 4 — Source data Fig. 2 [file 44318_2025_431_MOESM4_ESM.zip › Figure 2 copy/2A/README.rtf]

The data points contained in Figure 2B were obtained by quantifying images using an in-house Matlab script. The script functions by applying a linear baseline correction to the fluorescent images before converting the images to binary based on a pixel intensity value chosen to most accurately match the objects in the image. The raw images are contained in the subfolder ‘InputData’. The images after processing and their respective segmentation masks are contained in the ‘ProcessedDataAndMasks’ folder. The quantification results are logged in the file ‘2B.xlsx’
